# Tracking telomere fusions through crisis reveals conflict between DNA transcription and the DNA damage response

**DOI:** 10.1093/narcan/zcaa044

**Published:** 2021-01-06

**Authors:** Kate Liddiard, Julia W Grimstead, Kez Cleal, Anna Evans, Duncan M Baird

**Affiliations:** Division of Cancer and Genetics, Cardiff University School of Medicine, Heath Park, Cardiff, CF14 4XN, UK; Division of Cancer and Genetics, Cardiff University School of Medicine, Heath Park, Cardiff, CF14 4XN, UK; Division of Cancer and Genetics, Cardiff University School of Medicine, Heath Park, Cardiff, CF14 4XN, UK; Wales Gene Park, Institute of Medical Genetics, Cardiff University School of Medicine, Heath Park, Cardiff, CF14 4XN, UK; Division of Cancer and Genetics, Cardiff University School of Medicine, Heath Park, Cardiff, CF14 4XN, UK

## Abstract

Identifying attributes that distinguish pre-malignant from senescent cells provides opportunities for targeted disease eradication and revival of anti-tumour immunity. We modelled a telomere-driven crisis in four human fibroblast lines, sampling at multiple time points to delineate genomic rearrangements and transcriptome developments that characterize the transition from dynamic proliferation into replicative crisis. Progression through crisis was associated with abundant intra-chromosomal telomere fusions with increasing asymmetry and reduced microhomology usage, suggesting shifts in DNA repair capacity. Eroded telomeres also fused with genomic loci actively engaged in transcription, with particular enrichment in long genes. Both gross copy number alterations and transcriptional responses to crisis likely underpin the elevated frequencies of telomere fusion with chromosomes 9, 16, 17, 19 and most exceptionally, chromosome 12. Juxtaposition of crisis-regulated genes with loci undergoing *de novo* recombination exposes the collusive contributions of cellular stress responses to the evolving cancer genome.

## INTRODUCTION

The finite proliferative capacity of human primary fibroblasts was first documented by Hayflick and Moorhead in 1961 (1) and attributed to intrinsic factors that resulted in a state of cellular senescence. The characteristic irreversible senescent growth arrest can be triggered by telomere attrition to a length at which chromosome ends are exposed as double-stranded DNA breaks (DSB) (2), activating RB1 and TP53 tumour suppressor cell cycle checkpoints (3). The senescent condition is accompanied by transcriptional (4,5) and morphological changes (6) that ultimately culminate in the secretion of inflammatory mediators and growth factors as part of the senescence-associated secretory phenotype (SASP) (7). Whilst representing an effective constraint on the propagation of genomic instability, senescence adversely impacts immune function, tissue ageing and tumour metastasis. In recent years, considerable effort has been exerted to identify means of uncovering and eliminating senescent cells to ameliorate diverse pathologies (8,9). More comprehensive appreciation of the multitudinous signalling pathways (10) that contribute to the pro-inflammatory capacity and apoptotic resistance of senescent cells would undoubtedly expedite viable therapeutic approaches (11,12).

Bypass of replicative senescence can be achieved through the co-expression of human papillomavirus 16 (HPV16) E6 and E7 oncogenes (13–15) that subvert RB1 and TP53 function (16–18), disengaging cell cycle regulation. Human fibroblasts transformed with HPV^E6E7^ expression cassettes display extended proliferative lifespan and manifestations of telomere dysfunction, including fusion precipitated by non-homologous end-joining (NHEJ) DNA repair (19–21). The recombination of sister chromatid or heterologous chromosomal telomeres produces dicentric chromosomes that can engender far-reaching genomic instability through progressive breakage-fusion-breakage cycles during persistent mitosis (22). This predicament of exacerbated DNA damage and genomic rearrangement is referred to as ‘crisis’, driven by the aberrant recognition of deprotected chromosome ends as DSB (23). The appearance of telomere fusions constitutes a valuable diagnostic tool for the determination of crisis, being uncommon in normal mitotic and senescent cells. Although telomere fusions have been distinguished in human malignancy (24,25), additional biomarkers of the crisis state would be beneficial for the timely and unambiguous detection of oncogenic transformation *in vivo*. In experimental fibroblast models, crisis is transient and immortalization is not supported without supplementary mutations that stabilize telomere length (26). The elevated genomic instability fomented by telomere fusion provides substrates and opportunities for malignant mutagenesis; however escape from replicative crisis remains rare and terminal growth arrest or cell death prevails. Thus, these cells undergo significant phenotypic transitions whose delineation may facilitate modulation of crisis and senescent cell fate in health and disease.

Using a novel combination of single-molecule long-range multiplex polymerase chain reaction (PCR) and Illumina paired-end sequencing, we previously discovered an unexpected coincidence of inter-chromosomal telomere fusions with coding sequence (27). This association was verified for both telomere fusions effected by targeted endonucleases and those amplified from leukaemia patients (28). Furthermore, genomic loci with reported copy number alterations (CNA) in leukaemia were also incorporated into telomere fusions, suggesting that eroded telomeres sequester free DNA ends liberated by transcription- and replication-induced DSB (29). These former studies were limited in their reliance on inferred correspondence between transcription, replication and recombination. In our current research, we have undertaken parallel sequencing of telomere fusions, transcriptomes and individual genomes of human fibroblasts transiting a telomere-driven crisis in order to resolve the direct interactions that underpin genomic rearrangements. We have clarified the role of active transcription in creating genomic vulnerabilities that may enable prediction and manipulation of recombination hotspots and tissue-specific consequences in malignant disease. These molecular investigations provide insights into the transcriptional networks that initiate and reinforce replicative crisis and senescence (30,31), extending extant comprehension of processes pivotal to tumourigenesis and metastasis.

## MATERIALS AND METHODS

### Cells

All cells were routinely cultured at 37°C in 5% CO_2_, screened for the absence of mycoplasma and authenticated by morphology and genomic sequence.

The four human normal diploid fibroblast lines used in this study have been described previously (2,21,23,32). IMR90 (33) and WI38 (1) lines were obtained from the Coriell Institute Cell Repository and MRC5 (34) cells from European Collection of Authenticated Cell Cultures. HCA2 fibroblasts were gifted from James Smith, Houston, USA (35).

Supplementary experiments were conducted using human fibroblasts with defined mutations in DNA ligases obtained from the Coriell Institute Cell Repository: GM16088 and GM17523 derived from patients with DNA ligase 4 deficiencies and GM16096 derived from a patient with DNA ligase 1 deficiency.

Briefly, HCA2 were cultured in Dulbecco's modified Eagle medium supplemented with 10% (v/v) foetal calf serum (FCS). MRC5, GM16088 and GM16096 were cultured in Eagle's minimum essential medium (EMEM) supplemented with 1 × non-essential amino acids, 10% (v/v) FCS and buffered with 0.2% NaHCO_3_ solution. IMR90, WI38 and GM17523 were cultured in EMEM supplemented with 2 × non-essential amino acids, 15% (v/v) FCS and buffered with 0.2% NaHCO_3_ solution and 26 mM HEPES buffer.

All cultures were supplemented with 1 × 10^5^ IU/l penicillin, 100 mg/l streptomycin and 2 mM glutamine.

Retroviral gene transfer using amphotropic retroviral vectors containing HPV16 E6E7 (E6E7) or neomycin-resistance alone (NEO) expression cassettes has been outlined in (23) and selection of expressing cells was by culture in the presence of G418 at 0.4 mg/ml.

**Figure 1.**
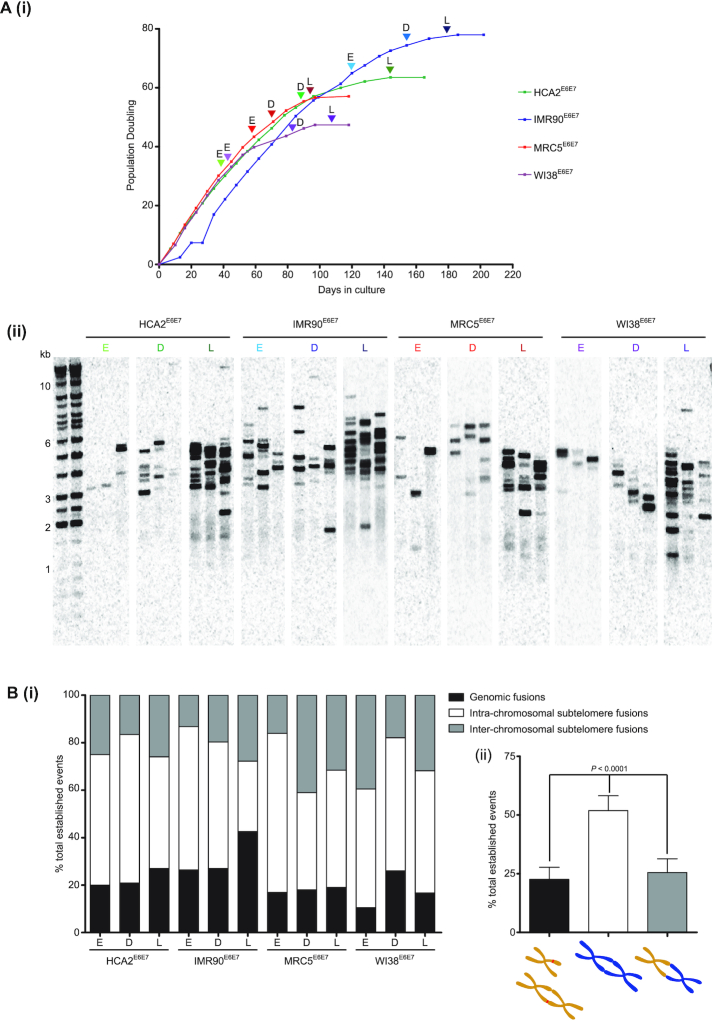
Characterization of a telomere-drive crisis in human fibroblasts retrovirally transduced with HPV16 E6E7. (**A**) (i) Growth curves for the E6E7-transformed fibroblast lines utilized in sequencing are displayed with arrowheads marking the Early (E), Deep (D) and Late (L) crisis sampling time points. (ii) Southern blot depicting telomere fusion amplicons generated using unidirectional primers specific for chr17p, chr21q and chrXpYp subtelomere sequences with fibroblast genomic DNA samples extracted at the crisis time points depicted in (i). Triplicate reaction lanes are presented for each sample time point, with molecular weight (MW) markers (in kb) on the left. Blots were hybridized with a radiolabelled probe consisting of chr17p subtelomere sequence. (**B**) (i) Stacked bar chart depicting proportions of all established fusions for each sample that are classified as genomic (black), intra-chromosomal (white) or inter-chromosomal (grey) at the E, D and L time points. (ii) Summary of proportions of specified fusions (depicted as cartoons below the chart) for all samples displayed with error bars showing 95% CI. Statistical significance was assessed by repeated measures one-way ANOVA with Tukey's multiple comparison post-test.

Cells were maintained at 70–85% confluency, with population doubling (PD) calculated at each passage following cell counts using an NC-3000 image cytometer (Chemometec). Viable stocks were cryopreserved at each passage for RNA and DNA sampling once full lifespan growth curves had been generated and retrospective delineation of Early, Deep and Late crisis points for each E6E7-transformed line had been performed (Figure 1Ai and Supplementary Figure S1A). The onset of telomere-driven crisis was defined by the appearance of telomere fusions (Figure 1Aii), accompanied by a progressive reduction in proliferation rate and morphological changes. All cultures were terminated when replicative senescence was reached after 14–21 days with no population growth.

The HCT116 human colorectal carcinoma cell line was cultured in McCoy's 5A medium supplemented with 10% FCS, 1 × 10^5^ IU/l penicillin, 100 mg/l streptomycin and 2 mM glutamine.

### Flow cytometry of G2 cell cycle phase isolated nuclei

For whole genome amplified sequencing (WGA-seq) of single nuclei from Deep crisis stage E6E7-transformed HCA2, IMR90, MRC5 and WI38 fibroblasts, nuclei were first isolated according to the protocol established by Zeng *et al.* (36).

Concisely, ∼1 × 10^6^ Deep crisis fibroblast cells were pelleted and resuspended in phosphate-buffered saline (PBS) for accurate assessment of mean cell diameter and viability using the Chemometec NC-3000 image cytometer ahead of nuclear isolation. Cells were lysed in buffer containing 10 mM Tris–HCl pH7.4, 10 mM NaCl, 3 mM MgCl_2_ and 0.005% IGEPAL CA-630 on ice for 20 min. Liberated nuclei were pelleted by centrifugation at 500 ×*g* for 20 min at 4°C. Completion and purity of lysis and extraction were verified by determination of expected reduction in particle diameter and viability, assessed by acridine orange loading (80 μg/ml). Purification was repeated by additional lysis or filtration through 20 μm sterile CellTrics filters where necessary. Purified nuclei were pelleted at 500 ×*g* for 20 min at 4°C and resuspended to 1 × 10^6^/ml in PBS supplemented with freshly prepared DAPI (4′,6-diamidino-2-phenylindole) at 125 ng/ml. Nuclei were incubated for 1 h in the dark at room temperature before 4N DNA content (G2 cell cycle phase) cells were sorted into single wells (preloaded with 4 μl PBS) of 96 well skirted plates (4titude 4ti-0740) on ice using a FACSAria III Cell Sorter. A bulk control sample was provided by sorting 500 pooled nuclei into a single well on each plate. Blank wells served as negative controls. Plates were briefly pulsed down before being sealed with solvent- and temperature-resistant adhesive lids (ThermoFisher AB1170), wrapped in parafilm and snap frozen in snap seal bags. Plates were shipped on dry ice to the Oxford Genomics Centre (Wellcome Centre for Human Genetics) for whole genome extraction, amplification and Illumina HiSeq4000 150PE (paired-end) sequencing. Twenty-three single nuclei and one bulk (500 nuclei) sample were sequenced for each fibroblast lineage.

### Genomic DNA extraction

Total genomic DNA was extracted from fibroblasts using standard Tris–HCl lysis buffer in the presence of proteinase K and RNase A, followed by phenol/chloroform purification, precipitation with sodium acetate and solubilization in 10 mM Tris–HCl (pH 7.5). Total genomic DNA was extracted from TALEN-treated HCT116 and fibroblast cells using Sigma GenElute™ Mammalian Genomic DNA Miniprep Kits and eluted in nuclease-free water. DNA was quantified using a NanoDrop One^C^ Spectrophotometer (ThermoFisher) to confirm a consistent quality and DNA yield for use in telomere length and fusion PCR and sequencing reactions.

### Telomere length PCR

Telomere length at chr17p was determined using 250 pg sample input genomic DNA in six replicate reactions following the Single Telomere Length Analysis (STELA) protocol with 17p telomere-adjacent and Teltail primers, as described (2,21,32). Following 0.5% TAE agarose gel electrophoresis, resolved telomere length amplicons were detected by Southern blotting using a random-primed α- (33) P-radiolabelled telomere repeat or telomere adjacent sequence probe in conjunction with a probe to detect the molecular weight markers. Quantification of chr17p telomere length was performed by Dr Kevin Norris (TeloNostiX, Cardiff University) using ImageQuant TL v 8.2 (Cytiva).

### Telomere fusion PCR and amplicon sequencing

Telomere fusion amplicons were generated from fibroblast and HCT116 sample input genomic DNA by multiplex long-range PCR using primers targeting the chr17p, chrXpYp and chr21q family of homologous telomeres (17p6, XpYpM and 21q1 primers, respectively (19,21,32).

For fibroblast fusion PCR employed to generate amplicon sequencing samples, genomic input was set at 25 ng/reaction (except for GM16096 DNA ligase 1 deficient fibroblasts, where 50 ng/reaction was employed), empirically determined to be an optimal balance between unique telomere fusion amplicon yield and limited genomic DNA needing to be subsequently removed from sequencing samples.

For HCT116 cell fusion PCR, genomic input was set at 50–100 ng/reaction and fusion frequency was calculated per diploid genome.

For TALEN-transfected fibroblasts, DNA input was adjusted to relative transfection efficiency (estimated from GFP expression) to control for minor variations in exogenous gene expression that might result from treatments. GFP transfections and carrier control samples were always performed to ensure validity of observations. Fusion frequency was evaluated as the mean number of non-constitutive fusion amplicons revealed by Southern blotting per reaction since input DNA and ploidy were variable amongst samples.

For standard fusion PCR experiments, three individual replicate reactions were typically resolved by 0.5% TAE agarose gel electrophoresis for visualization on Southern blots using radiolabelled telomere-adjacent probes specific for each telomere end.

For fibroblast fusion amplicon sequencing, 100 fusion PCR reactions per sample were prepared for quality control and purification of mixed amplicons ahead of Illumina paired-end sequencing. As described previously (27,37), three random PCR wells were selected per sample for amplification verification prior to the 100 replicates for each sample being pooled and purified using Agencourt AMPure XP magnetic beads with elution in nuclease-free water. Aliquots of each sample were taken pre- and post-purification for a comparative assessment and to check purification efficiency. Purified amplicons from HCA2, IMR90, MRC5 and WI38 fibroblasts were sequenced at the Oxford Genomics Centre (Wellcome Centre for Human Genetics) using HiSeq2500 v4 reagents and 125 bp PE reads. Purified amplicons from GM16088, GM16096 and GM17523 fibroblasts were sequenced at the Wales Gene Park using HiSeq2500 reagents and 150bp PE reads. Two replica lanes of each sample were run and data pooled for downstream analyses.

### Total RNA extraction

For RNA sequencing (RNA-seq), 1 × 10^6^ cells of each sample at the defined crisis time points were PBS washed and pelleted for RNA extraction using the Analytik Jena InnuPrep minikit. Sample quality was evaluated using the Agilent Bioanalyzer to reveal RIN values, 28S:18S ratios and precise concentrations ahead of sending for sequencing. All samples achieved an RIN > 9.9. Illumina HiSeq4000 75PE RNA-seq was conducted by the Oxford Genomics Centre (Wellcome Centre for Human Genetics).

### Data visualization

Indexed BAM files for all fusion amplicon sequence, RNA-Seq and single nuclei WGA-seq data filtered read pairs were prepared for visualization in IGV (38) against Ensembl and Refgene sequence tracks. For determination of coincident fusion, transcription and/or CNA, IGV sample tracks were multiplexed in a single window. All genomic coordinates are appropriate to the December 2013 GRCh38/hg38 human reference assembly.

Custom tracks depicting Repeatmasker (www.repeatmasker.org) repetitive DNA motifs, fragile sites and NFAT transcription factor binding sites were extracted as BED files from the UCSC Genome Browser tool (https://genome.ucsc.edu/cgi-bin/hgTables), the TFmotifView webtool (39) and the HUMCFS database (40).

Sequence-authenticated telomere fusion junctions were prepared as BED files for Ensembl (41) karyotype plots, as well as IGV alignments.

### Ontology and interaction network searches

Lists of unique genes disrupted by telomere fusions (fusion amplicon-seq data) and the top 50 differentially expressed genes for each crisis sample (RNA-seq data) were compiled as text files for ontology searching using the gene ontology (GO) database (release date 21 February 2020) and Gene Set Enrichment Analysis (GSEA) tools (MSigDB 7.1; 2020). Common regulatory networks potentially underpinning these gene lists were identified using the X2K Expression2Kinases webtool (42) and the STRING protein–protein interaction database (43).

### Statistical analyses

All statistical analyses, including one- and two-tailed t-tests, Mann–Whitney U-tests, Kruskal–Wallis and Repeated Measures ANOVA tests, Chi square and Fisher's exact tests were performed using GraphPad Prism 6 and GraphPad QuickCalcs. Comparison of means was computed using Medcalcs webtools. Appropriate tests were selected according to whether data were collected as paired or independent experimental samples, whether data distributions were Gaussian or skewed and whether variance between comparators was equal. Data are presented as means with 95% confidence intervals (CI) except where it was considered important to display the spread of the data by standard deviation (SD). One-tailed *t*- and u-tests were applied where required to specifically assess a pre-defined direction of the biological effect under consideration. For all tests, a *P-*value of <0.05 was considered statistically significant.

### Supplementary methods

Descriptions of additional methods and analyses employed in this study, including sequence alignments and bioinformatics approaches, are provided in the accompanying Supplementary Data.

## RESULTS

### Progression through telomere crisis is accompanied by transitions in DNA repair

We previously observed an elevated frequency of coding sequences incorporated into telomere fusions compared with intergenic loci (27,28). To establish the cause of this association, we employed established fibroblast senescence bypass models to map *de novo* telomere fusions and transcriptional responses in parallel. Four human fibroblast lines (HCA2, IMR90, MRC5 and WI38) were retrovirally transduced with HPV16 E6E7 (to suppress DNA damage checkpoints (15)) and/or neomycin resistance expression cassettes and cultured up to 7 months for serial sampling of populations at Early, Deep and Late time points of telomere-driven crisis or replicative senescence (Figure 1Ai and Supplementary Figure S1A), as defined by their respective growth trajectories. The E6E7-transduced cells underwent a mean of 29.8 (SD 8.39) more PDs more than their NEO control counterparts and demonstrated telomere shortening prior to crisis onset that was ongoing throughout crisis progression (Supplementary Figure S1B and Table S1). The advance through crisis was delineated by progressive incidence of telomere fusions amplified with chr17p, chr21q (family) and chrXpYp subtelomere-specific primers (19,21,32) for all E6E7-transformed fibroblasts (Figure 1Aii and B), but rarely from the NEO controls (Supplementary Figure S1C). Purified RNA and uncharacterized telomere fusion amplicons were subjected to Illumina paired-end sequencing (RNA-seq and Fusion-seq, respectively; Supplementary Table S2). In addition, nuclei were extracted from deep crisis-stage fibroblast populations and subsequently enriched for G2 cell cycle phase following sorting by flow cytometry based on DNA content. Isolated single nuclei underwent DNA extraction and whole genome amplification to determine gross chromosomal recombinations and CNA by Illumina paired-end sequencing (WGA-seq) in corollary with the genomic instability exemplified by telomere fusions.

**Figure 2.**
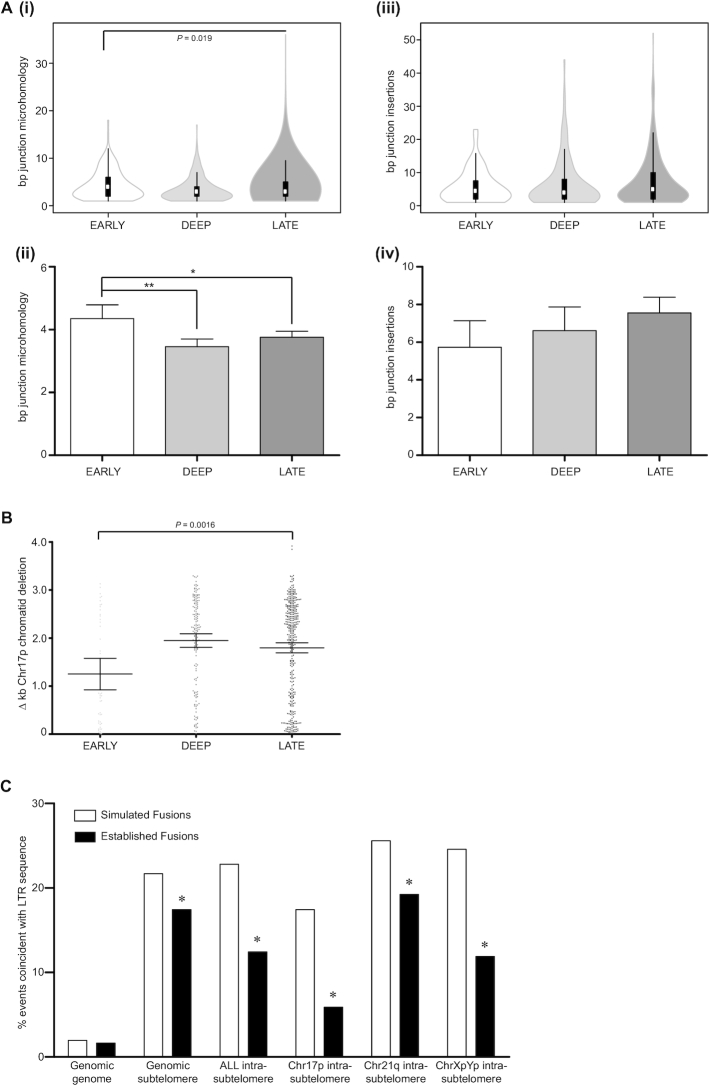
Progressive changes in telomere fusion architecture throughout crisis. (**A**) Violin (114) (density estimate) plot summary of (i) bp MH and (iii) bp INS at all junctions of all fusion categories and samples segregated by crisis time point. For each polygon, white circles represent the median, box limits indicate the 25th and 75th percentiles and whiskers extend 1.5 times the interquartile range. Mean bp junction (ii) MH and (iv) INS for each crisis time point is also displayed in a bar chart with 95% CI. Statistical significance was determined by Kruskal–Wallis one-way ANOVA with Dunn's post-test analysis and graphs annotated with *P*-values or significance indicators (*P< 0.05, **P< 0.01). (**B**) Summary of difference (Δ) kb deletion at fused chromatid pair constituents of chr17p intra-chromosomal fusions for all samples separated by crisis phase (E, D, L). Error bars with means and 95% CI are shown and statistical significance was determined by Kruskal–Wallis one-way ANOVA with Dunn's post-test analysis. (**C**) Reduced incidence of established (black bars) telomere fusion junctions within subtelomere LTR (and not genomic LTR) sequences compared with a control set of >13 000 bioinformatically simulated (white bars) telomere fusions. The statistical significance of differential frequencies of genomic (genome or subtelomere read) and intra-chromosomal fusion junction coincidence with LTR was determined by chi-squared analysis; *P< 0.05.

As previously (27), three distinct categories of telomere fusions could be distinguished by amplicon sequencing (Figure 1B); genomic fusions (between telomeres/subtelomeres and non-telomeric loci), intra-chromosomal (sister chromatid) telomere/subtelomere fusions and inter-chromosomal (disparate chromosome partners) telomere/subtelomere fusions. The reciprocity of chr21q subtelomere family sequences encumbered accurate discrimination of true sister chromatid from potential inter-chromosomal fusions, however, intra-chromosomal fusion status was assigned only where homology with the archetypal 21q subtelomere sequence (and not related subtelomere sequences) was predominant. Intra-chromosomal fusions were 2-fold more prevalent than other classes (*P* < 0.001; Figure 1Bii) and linkages to chr17p and chr21q family telomeres far exceeded linkages to the chrXpYp telomere for all types of fusions (Supplementary Figure S2). Established telomere fusion junctions were examined for evidence of microhomology (MH) usage and presence of nucleotide insertions (INS) (Figure 2A and Supplementary Figure S3) (44,45) that are informative of underlying DNA repair processes (19). Consistent with DNA repair mediated by alternative NHEJ (ANHEJ), more than 60% junctions of each class of event were characterized by the presence of junctional MH (Supplementary Figure S3A–C). Additionally, INS templated from sequence flanking (±100 bp from junction) the fusion junctions comprised ≥50% all INS at genomic fusion junctions (Supplementary Figure S3D), although these were significantly (*P* < 0.0001) more scarce at both intra- (10.3%, SD 10.02) and inter-chromosomal telomere fusions (6.6%, SD 14.17). The average length of MH usage reduced with passage through crisis (1.25-fold early-late crisis; *P* = 0.019) (Figure 2Ai and Aii; Supplementary Figure S3E), whereas there was a trend towards longer INS (Figure 2Aiii and Aiv; Supplementary Figure S3F).

To investigate potential mechanisms for this skew in DNA repair during the progression through crisis, we compared the MH and INS observations from our normal fibroblast dataset with analogous data derived from fibroblasts of patients with DNA ligase 1 and 4 mutations (Supplementary Figure S3G and H). In accordance with our previous findings using gene-targeted cancer cell lines (27,37), we discovered a relative decrease in the fraction of genomic compared with intra-chromosomal fusions recovered from the DNA ligase 4-deficient cells with compromised classical NHEJ (CNHEJ) capacity (Supplementary Figure S3I). Conversely, fusions derived from DNA ligase 1-deficient fibroblasts were prominently depleted of intra-chromosomal events. Genomic telomere fusions amplified from these DNA repair-defective fibroblasts in crisis revealed significantly greater proportions of genomic fusions with junctional MH and lower incidence of events with INS compared with the normal fibroblasts (Supplementary Figure S3J and K). Strikingly, the length of both features was notably reduced (1.23- and 1.62-fold for MH and INS, respectively; *P* = 0.008 and *P* = 0.0102) at genomic fusions amplified from the DNA ligase 1-deficient cells. MH usage (and to a lesser extent, INS magnitude) was similarly contracted at intra- and inter-chromosomal fusions identified in DNA ligase 1-deficient cells (Supplementary Figure S3L and M), intimating a correlation between the decline in DNA replication and MH-mediated repair during crisis.

We have previously documented length asymmetry of sister chromatids comprising intra-chromosomal telomere fusions, suggestive of differential deletion of participating subtelomeres prior to fusion (27). Concordant with the evolving DNA repair profiles presently observed during crisis, we also detected enhanced asymmetric processing of fused sister chromatids in intra-chromosomal fusions (Supplementary Figure S4A and B) that were especially evident at the unique chr17p telomere (Figure 2B; 1.56- and 1.44-fold increases in differential fused chromatid deletion for early-deep and early-late crisis samples, respectively, *P* = 0.0016). To explore whether the distribution of fusion junctions over the reference genome could be explained by simple biases in short-read mapping or variant calling at subtelomeric loci, we compared our biological datasets to a synthetic dataset of simulated telomere fusions (simulants) resolved using identical methodology. Established fibroblast fusion junction coordinates were plotted along their corresponding subtelomeric references and juxtaposed with this substantial dataset of simulated fusion junctions (expressed as fusion junction frequency distributions) to expose regions replete or restricted in fusion events (Supplementary Figure S4C). Telomere-proximal sequences consisting of variant telomere repeats were devoid of fusion junctions, although a subset of fusions between chr21q family sister chromatids did incorporate telomere sequence (Supplementary Figure S4Cii). There was also a striking relative paucity of fibroblast fusion junctions (but not simulant controls) approximately mid-way between the fusion-amplifying primer and the telomere for each chromosome end. Cross-reference with the Repeatmasker database (www.repeatmasker.org) identified coincident or adjacent long terminal repeats (LTR); LTR36 at chr17:114646–115151, LTR60B at chr21:46697034–46697250 and LTR12D at chrX:10042–10446, suggesting possible sequence-dependent constraints over sites of subtelomeric DSB or repair. Scrutiny of the coincidence of all genomic and intra-chromosomal fusions with LTR repeats recapitulated this dearth of established fusion junctions within subtelomere, but not genomic sequences (Figure 2C). Additionally, the number of single nucleotide variations (14) between the fibroblasts and the GRCh38/hg38 reference across the 1.2 kb region of low-coverage within the chr17p subtelomere (chr17: 14400–15600) was identical to that in the 2.2 kb flanking sequence profuse in fusion junctions. These observations dispel concerns that the asymmetric junction distributions issue from alignment artefacts.

In contrast to the normal fibroblasts, mapping intra-chromosomal fusions amplified from DNA ligase-deficient fibroblasts exposed a noticeably homogenous distribution of junctions along the unique chr17p subtelomere sequence for the samples with DNA ligase 1 mutations (Supplementary Figure S4Di). When charted by frequency (Supplementary Figure S4E), intra-chromosomal fusion junctions within the chr17p central subtelomeric region (chr17: 14400–15600) were 1.28-fold (*P* = 0.0243) more abundant for the DNA ligase 1-deficient than the normal fibroblasts. Thus, length asymmetry of fused sister chromatids may be coupled with replication, as well as end-joining, mediated by DNA ligase 1 (37).

### Telomere fusions coincide with actively transcribed loci

**Figure 3.**
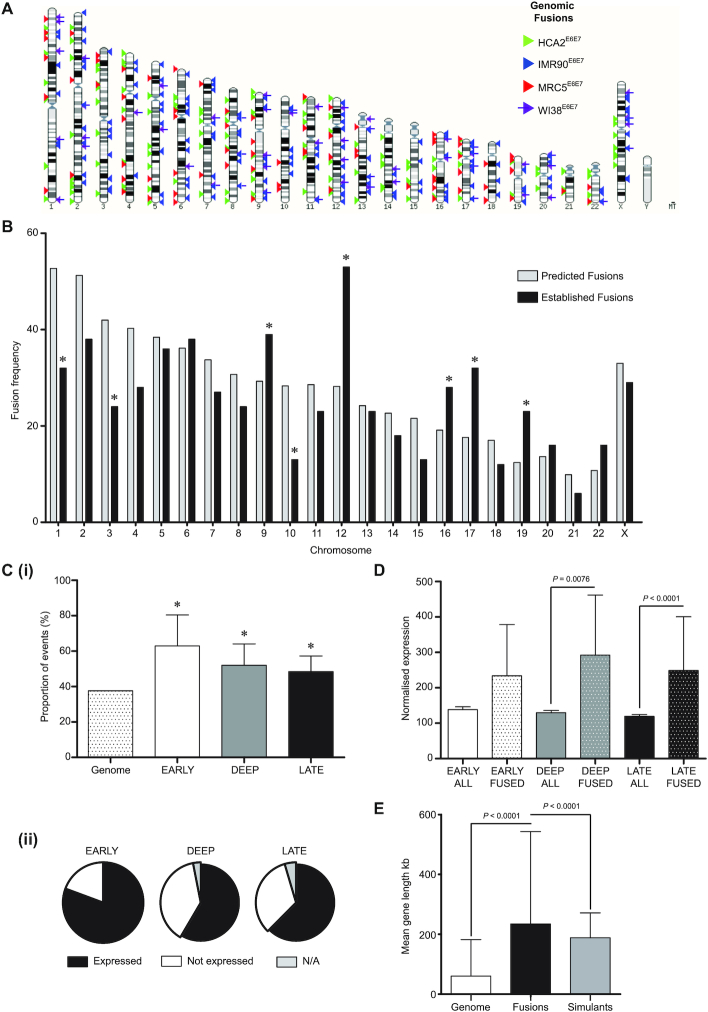
Genomic telomere fusions are associated with long transcribed genes and enriched at chr12. (**A**) The junction coordinates of each established genomic telomere fusion for each crisis fibroblast model were converted into BED files for karyotype display. Arrowheads signify unique fusion events for HCA2 (green), IMR90 (blue), MRC5 (red) and WI38 (purple with shaft) fibroblast lines. (**B**) The numbers of all sample genomic fusions localized to each chromosome and adjusted for chromosome size (in kb) are shown as a bar chart. ChrY was omitted since IMR90 and WI38 are homogametic XX. ChrMT was excluded on the basis of unknown copy number. Predicted (grey bars) fusion frequencies (events/chromosome) were generated using the mean overall fusion frequency (events/kb) multiplied by the specific chromosome size for comparison with the actual (black bars) normalized chromosome frequencies. A chi-squared analysis was used to compare predicted with actual frequencies, revealing statistically significant (*P*< 0.05) variation at chromosomes 1, 3, 9, 10, 12, 16, 17 and 19 (marked with *). (**C**)(i) The proportions of all genomic fusions with junctions in gene sequence are plotted by crisis stage (with error bars showing 95% CI) and compared with the proportion of the whole genome that consists of genes (patterned bar, left) by chi-squared analysis (significant differences marked with *; *P*< 0.05). (ii) Pie charts displaying the proportions of fused genes at distinct crisis phases that were expressed (black) or not (white) or for which data were ambiguous (grey) in crisis fibroblasts, as detected by RNA-seq. (**D**) Mean transcript RNA-seq DESeq2 normalized read counts for genes fused (FUSED) to telomeres at each crisis stage are presented in comparison with mean transcript read counts for unaffected genes (ALL) at the same time points with 95% CI. Statistical significance was assessed using a comparison of means test. (**E**) The mean gene length (60.45 kb; white bar) within the human genome GRCh38 reference was calculated using GeneBase 1.1 and compared with the mean length of all genes captured within telomere fusions in our crisis fibroblast dataset (234.32 kb; black bar) and simulated dataset (233.00 kb; grey bar) using a comparison of means test to evaluate statistical significance. Error bars depict SD.

Telomere fusion junctions dispersed around the genome were investigated for conjunction with coding sequence (Figure 3). We previously reported associations of telomere fusions with genes in both experimental models (27) and samples from patients with chronic lymphocytic leukaemia (CLL (28)), raising the question as to whether these loci were sites of active transcription at the point of DSB or fusion. By conducting RNA-seq in parallel with fusion amplicon sequencing from the same fibroblast populations transiting crisis, we hoped to address this salient point. Established unique telomere-genomic fusion junctions for each fibroblast model displayed as a karyotype plot (Figure 3A) highlight the comprehensive coverage of the genome, whilst also exposing specific chromosomes with greater frequencies of events. Once adjusted for the discrepant chromosome sizes, fusion incidence was re-charted in comparison with a predicted fusion rate per chromosome based on mean genomic fusion frequency (Figure 3B). This analysis revealed a particular enrichment of fusions on human chromosomes 9, 12, 16, 17 and 19, with chromosomes 1, 3 and 10 demonstrating lower fusion frequency than expected. These observations were reinforced by comparisons with our simulated fusion dataset (Supplementary Figure S5A), which paralleled the predicted distribution, underlining the biological origins of the perspicuous divergences.

Dispersing established fusion events according to the cellular models in which they arose unveiled the exceptional abundance of telomere fusions with chr12 in the IMR90 fibroblast line (Supplementary Figure S5B). Notwithstanding the individual fusion signatures of these crisis fibroblasts, an elevated frequency of fusions involving chromosomes 12, 17 and 19 was universally observed. Charting the total (Ensembl; Supplementary Figure S5Ci and ii) and cancer-associated (Cancer Genetics*Web* (46); Supplementary Figure S5Ci and iii) gene densities of each chromosome upheld the postulated relationship between coding sequence and fusion propensity, with chromosomes 17 and 19 scoring highest on all indices. Whilst the gene and cancer-associated gene densities of chr12 exceeded the mean values for all chromosomes (Supplementary Figure S5C), supplementary factors, including gene transcription and replication timing (47) may contribute to the outstanding fusion frequency recorded for the IMR90 fibroblasts at this chromosome (Supplementary Figure S5B).

The proportions of all sample genomic fusion junctions coincident with coding sequence (as defined by Ensembl and Refgene annotation) were significantly greater than the overall proportion of the human genome that is composed of genes (Figure 3Ci and Supplementary Figure S6Ai). Fusion within exon sequence was rare (1.7% total genomic events; Supplementary Figure S6Ai), consistent with the low proportion of the whole genome that is exons recorded in GeneBase 1.1 (48) (1.74%). Manual curation of each telomere recombination with coding sequence (Supplementary Figure S6Aii) revealed fusion with enhancers (4.11%), promoters (5.14%) and promoter-flanking sequence (13.01%) occurred more recurrently than within exons (2.74%), raising the possibility that localized chromatin reorganization by transcriptional regulators precipitates aberrant DNA interactions (49) and repair (50). By correlation with RNA-seq data sampled from the same fibroblast populations, we discerned that 63.5% genes (Supplementary Figure S6Aiii) or 58.3% exons (Supplementary Figure S6Aiv) incorporated into telomere-genomic fusions were actively transcribed at the same crisis stage. This conjunction varied with crisis stage, however; the fraction of fused genes that was actively expressed was lowest (58.5%) at the deep crisis time point (Figure 3Cii), whereas more than 80% of fused genes were transcribed in the early crisis-stage cells.

A profound correspondence between gene expression and fusion incidence was highlighted by comparing normalized RNA-seq expression counts for all transcripts at each crisis stage with those pertaining to genes fused with telomeres at the same experimental time points (Figure 3D). In early, deep and late crisis-stage fibroblasts, the mean expression counts for fused genes were 1.71-(NS), 2.27-(*P* = 0.0076), and 2.11-(*P* < 0.0001) fold higher than the overall stage-specific mean expression levels, respectively, although statistical significance was only returned for the later time points, owing to the substantial variation in read counts from non-transcribed and highly expressed transcripts. Interrogation of chr12 genes fused with telomeres captured explicitly within IMR90 samples (with especially elevated chr12 fusion frequency) delivered a 1.78-fold greater mean transcript expression level than that computed for fused genes on all other chromosomes, but likewise fell short of statistical significance (*P* = 0.4839; Supplementary Figure S6Bi). Gross chromosomal expression analyses broadly complemented associations between fusion frequency and coding sequence, with chromosomes 12 and 17 demonstrating 1.42-(*P* < 0.0001) and 1.36-(*P* < 0.0001) fold elevated mean transcript read counts, respectively, compared with the remaining bulk (Supplementary Figure S6Bii). Chromosome 19 countered these observations with a 1.39-fold (*P* < 0.0001) lower mean transcript expression than the genome (excluding chr19) average, suggesting repressive chromatin interactions may also stimulate recombination with damaged telomeres. Chromosome 8 served as an important negative control, presenting mean transcript expression (Supplementary Figure S6Bii), gene density (Supplementary Figure S5C) and telomere fusion frequency that approximated the genome average and simulated predictions (Figure 3B and Supplementary Figure S5B).

When calculating the length of genes disrupted by telomere-genomic fusions (Figure 3E and Supplementary Figure S6C), we observed an over-representation of genes longer than the mean human gene length of 60.45 kb (SD 121.90 kb; GeneBase 1.1). At 234.22 kb (SD 308.72 kb), the mean length of fused genes in our datasets was 3.87-fold (*P* < 0.0001) greater than the genome average and, importantly, 1.24-fold (*P* < 0.0001) greater than the mean length of genes featured in our simulated dataset (mean length 188.30 kb; SD 83.13 kb). Moreover, the simulated fusion junctions displayed reduced association with both coding (Supplementary Figure S6Di) and repeat (Supplementary Figure S6Dii) sequences compared with the established fibroblast fusions. Long genes co-exist with fragile sites (51), resulting in instability as a consequence of replication and transcriptional clashes (29,52). However, the frequency of telomere-genomic fusion junctions within fragile sites was indistinguishable from both the proportion of the total genome content that is occupied by fragile sites (40) (33.4% compared with 37.5%, respectively, *P* = 0.0504) and the coincidence of the simulated fusions with these loci (31.7%, *P* = 0.3981; Supplementary Figure S6Diii).

### Copy number alterations are foci of telomere fusion

**Figure 4.**
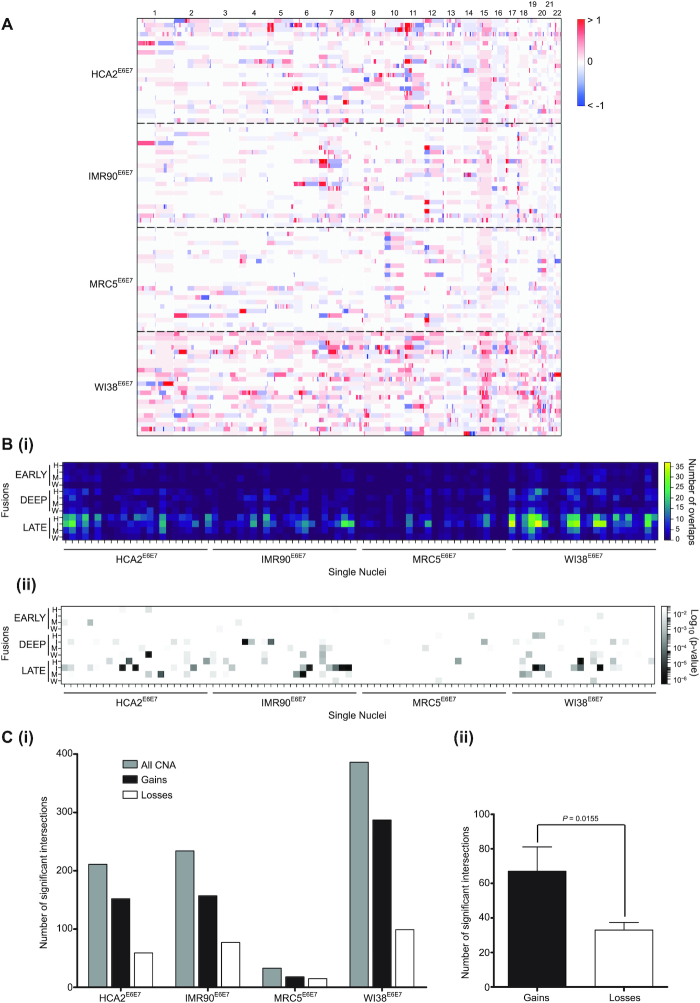
CNA in crisis genomes present significant intersection with telomere fusion loci. (**A**) 23 single 4N DNA content nuclei from each Deep crisis fibroblast sample were FACS-isolated into 96 well plates for whole genome amplification and single nuclei Illumina paired-end sequencing. A baseline reference was generated using a pool of 500 nuclei from the same sample sorted by the same methodology into a single well. A heatmap of genome copy number gains (red) and losses (blue) for each nucleus of each fibroblast line is presented (*y*-axis) with chromosomes ordered left to right (*y*-axis). (**B**)(i) All intersections between genomic loci displaying CNA and genomic telomere fusion junctions are depicted as a heatmap of single nuclei samples along the *x*-axis against fusion samples grouped by crisis phase (Early, Deep, Late) on the y axis; H, I, M and W represent HCA2, IMR90, MRC5 and WI38 fibroblast samples, respectively. The colour-coded frequency of overlap is described in the key shown on the right. (ii) Intersections determined as statistically significant by two-tailed Fisher's Exact test are displayed in the same format as for (i) with the Log_10_ (*P*-value) key on the right. (**C**)(i) The numbers of all statistically significant (*P*< 0.05) CNA intersections with genomic telomere fusions are plotted in a bar chart with for each crisis fibroblast sample (grey bars). Intersections with copy number gains are depicted as black bars and copy number losses as white bars. (ii) The total number of significant genomic telomere fusion intersections with copy number gains (black) and losses (white) is shown with 95% CI and statistical significance determined by one-tailed paired t-test.

To examine the inter-relationship of telomere fusions, gross genomic recombinations and gene expression changes with passage through crisis, we isolated single nuclei from each fibroblast population in deep crisis for whole genome sequencing. The genotypes of 23 4N DNA content deep crisis stage nuclei for each of the four fibroblast lines were normalized to a pool of 500 nuclei from the same sample to reveal regions of gross CNA (Figure 4A) during this phase of extensive genomic instability. Genomic locations occupied by both telomere fusions and CNA were identified (Figure 4Bi) and the significance of intersection was evaluated by a two-tailed Fisher's exact test (Figure 4Bii). Inspection of these data confirmed variable susceptibility inherent to each fibroblast line (Figure 4Ci, Supplementary Figure S7Ai), although a common predominance of copy number gains rather than losses (2-fold higher; *P* = 0.0155, Figure 4Cii) (53). Significant overlaps incremented with crisis transition, so that the enhanced proportions of deep crisis stage CNA intersections with late-stage telomere fusions (Figure 4Bii and Supplementary Figure S7Aii) could signify a causal relationship (54,55). When adjusted for the overall escalation in established telomere fusion frequency, however, the observed relationship between CNA-fusion overlaps with crisis stage was not significantly different from predictions (Supplementary Figure S7Aiii).

Since we had recognized individual chromosomes with telomere fusion densities raised above the mean expected (Figure 3B and Supplementary Figure S5A-B), we separated statistically significant intersections by genomic location (Supplementary Figure S7B). Chromosomes 6, 7, 9, 11, 12 and 17 were found to exceed the mean intersection rate of 39.23 events/chromosome (Supplementary Figure S7Bi). This remained true when accounting for chromosome physical size (Supplementary Figure S7Bii), but the subgroup was expanded to include chromosomes 19 and 22 and the more marginal cases, chromosomes 15, 18 and 20. These results indicate chromosome-specific vulnerabilities to genomic instability. When adjusted by fusion frequency per chromosome (Supplementary Figure S7Biii), these conspicuous enrichments were somewhat moderated, suggesting CNA and fusion incidence are inter-related. Chromosomes 12 and 17 prevailed as those experiencing most elevated frequencies (per Mb) of recombination by telomere fusion (Figure 3B) and CNA (Supplementary Figure S7Bii). Additionally, these two chromosomes demonstrated raised mean transcript expression levels compared with the remaining genome (Supplementary Figure S6Bii), implicating a connection between transcription and genomic instability at both focal and global scales. Since a primer targeting chr17p primer was employed in the initial amplification of telomere fusions for sequence analysis, there is a plausible ascertainment bias in the abundance of chromosome 17 aberrations. Thus, we focused on chromosome 12 as representing an intriguing and unusual focus of genome instability in these fibroblast samples transiting a telomere-driven crisis.

### The cellular transcriptome governs genomic recombinations that impact crisis outcome

**Figure 5.**
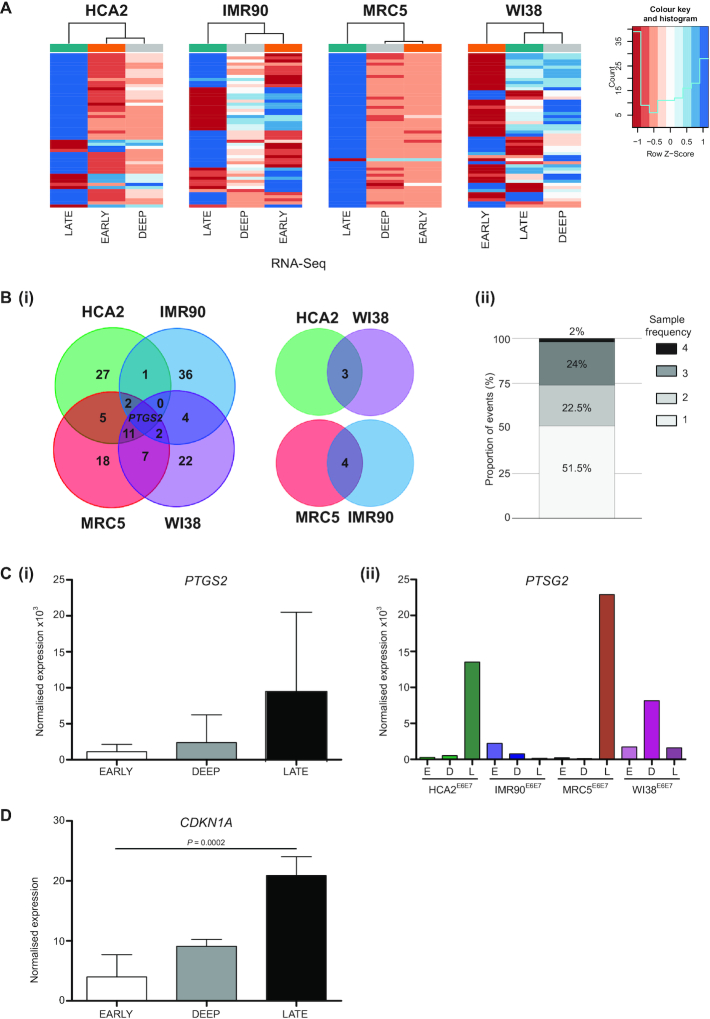
Genes differentially expressed with crisis progression are associated with DDR. (**A**) Heatmaps representing the top 50 differentially expressed genes measured by RNA-seq during crisis transit in HCA2, IMR90, MRC5 and WI38 fibroblasts. Samples from the three crisis time points are displayed as a dendrogram. Refgene gene identities and genome locations are detailed in the Supplementary information. (**B**)(i) A Venn diagram depicts the numbers of overlapping top 50 differentially regulated genes in particular fibroblast samples. Ancillary 2-way Venn charts reveal genes shared by samples non-adjacent in the 4-way Venn presentation. *PTGS2* is the sole gene featured on the gene lists of all four fibroblast samples. (ii) The proportions of top 50 differentially expressed genes measured by RNA-seq during crisis that are common to 1, 2, 3 or all 4 fibroblast samples are represented as a stacked bar chart. (**C**)(i) RNA-seq DESeq2 normalized read counts for *PTGS2* are displayed for pooled crisis fibroblast samples at Early, Deep and Late crisis stages. Results are plotted as means with SD and statistical significance tested by repeated measures one-way ANOVA with Tukey's multiple comparison post-test. (ii) Normalized expression of *PTGS2* plotted for each individual fibroblast sample and crisis time point, Early (E), Deep (D) and Late (L). (**D**) RNA-seq DESeq2 normalized read counts for *CDKN1A* are displayed for pooled crisis fibroblast samples at Early (E), Deep (D) and Late (L) crisis stages. Results are plotted as means with SD and statistical significance tested by repeated measures one-way ANOVA with Tukey's multiple comparison post-test.

The observation that more than 60% genes incorporated into telomere fusions were transcribed in the same sampled populations (Figure 3Cii and Supplementary Figure S6A), combined with the discovery that telomeres fuse with long genes, solicited questions concerning the interaction of DNA replication, repair and transcription (29,56). We, therefore, sought to thoroughly explore our crisis fibroblast RNA-Seq data by correlation across fibroblast samples and with fusion amplicon and whole genome sequencing datasets. Heatmaps of the top 50 regulated genes for each sample are displayed in Figure 5A and in detail in Supplementary Figure S8A–D, revealing mutual expression of inflammatory mediators associated with the SASP (10,11,30). For three of the fibroblast models, the early and deep crisis expression profiles were more closely related by hierarchical clustering than they were to the late crisis stage sets representing crisis resolution. The WI38 samples differ (Supplementary Figure S8D), insofar as the deep and late stage signatures are more similar than the early stage set. This conceivably reflects the relatively compressed cell culture period between the deep and late sampling points for this fibroblast line (Figure 1A) and is complemented by the augmented intersections between deep stage WI38 single nuclei CNA and late crisis stage telomere fusions (Figure 4B).

Nearly 50% of the top 50 regulated genes for each sample (Supplementary Table S3) were shared by at least two lineages (Figure 5B), with MRC5 and WI38 samples displaying the most overlaps (21 genes in common; Figure 5Bi). Prevalent features of late crisis samples include elevated expression of cytokines, chemokines and mediators of Notch and calcium signal pathways, contrasting with diminished levels of structural components including laminin, collagens, claudins and cadherins (Supplementary Table S3). STRING (43) protein–protein interaction network analysis of these common gene lists underscores the pro-inflammatory context that accompanies telomere-driven crisis and reveals potential targets for modulation of malignant transformation (Supplementary Figure S8E). Prostaglandin-endoperoxide synthase (*PTGS2*; also known as *COX2*) was the sole gene differentially regulated by all four fibroblast lines (Figure 5Bi and Ci), exhibiting an 8.6-fold increase in mean expression with progression from early to late crisis. Inter-sample variability was evident, however, with peak expression occurring at disparate stages for the individual samples (Figure 5Cii). Regulated by both TP53 (57) and Notch (58) signalling components, this gene epitomizes the convergence of cellular activation pathways during crisis. Whilst there was no measurable change in *TP53* RNA-Seq read counts with crisis progression (data not shown), escalated expression of prominent target genes including *PTGS2* and *CDKN1A* (5.22-fold upregulated early-late crisis, *P* = 0.0002; Figure 5D) provide indirect evidence of the ongoing DNA damage response (DDR).

Genomic coordinates were extracted for these crisis-regulated gene sets and unique entries were plotted by chromosome juxtaposed with telomere fusion junctions for visualization of correspondence (Supplementary Figure S8Fi). Chromosome frequencies of differentially expressed genes were evidently linked with chromosome size, the acrocentric chromosomes 13, 14, 15, 21 and 22 harbouring the lowest numbers and chromosome 1 the highest. Incidence was subsequently presented as a factor of the gene density of the respective chromosomes (Supplementary Figure S8Fii), unmasking chromosomes 8 and 18 as exceptional in their relative abundance of genes with altered expression during telomere-driven crisis. Of these, chromosome 18 may be delusory since the enumeration of differentially expressed genes is likely inflated by its remarkably low gene density (only 938 genes over 80Mb; GeneBase 1.1). In contrast, the array of genes localized to chromosome 8p, considered a hotspot for copy number polymorphisms and exacerbated mutation rate (59), may emphasize the importance of mutation in the evolution of genes implicated in responses to environmental stresses, including crisis and senescence (60).

The biological functions of genes differentially expressed and fused with telomeres during crisis were inspected to determine whether changes in cellular responses could be associated with genomic vulnerability (Supplementary Tables S4–7). Both GO (http://geneontology.org/) and GSEA (https://www.gsea-msigdb.org/gsea/index.jsp) searches highlighted significant associations between the genes differentially expressed in crisis and inflammatory and senescence pathways (Supplementary Tables S4 and 5). Candidates selected based on consonant regulation across samples were individually corroborated using the RNA-seq data (Supplementary Figure S8G), evidencing the increased expression of inflammatory SASP elements in the late crisis-stage samples (61). Genes captured within telomere fusions did not comprise such a coherent group (Supplementary Tables S6 and 7), with significant enrichments determined only for cytoskeletal organization processes. To determine if these distinct gene sets converged at a higher regulatory level, we employed the X2K eXpression2Kinases (42) webtool to identify putative transcription factors implicated in the regulation of the genes differentially expressed (Supplementary Table S8 and Figure S8Hi) and the genes fused with telomeres (Supplementary Table S9 and Figure S8Hii) during crisis. Mutual transcription factors revealed by these searches included nuclear hormone receptors (AR, ESR1), inflammatory response mediators (NFE2L2) and the DDR master regulator, TP53, demonstrating that genes critical to the cellular responses to DNA damage and stress are also susceptible to the DNA damage and aberrant repair that underpins tumourigenesis and clonal evolution in cancer (Supplementary Figure S8I).

### Chr12 instability impacts cellular response to telomere-driven crisis as a compound but not singular insult

**Figure 6.**
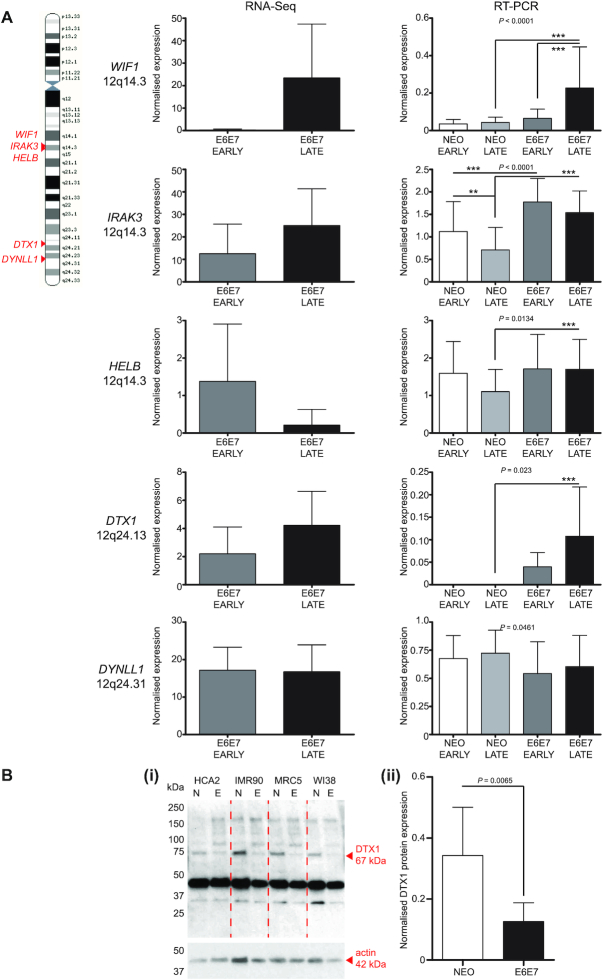
Crisis samples demonstrate elevated instability at chromosome 12. (**A**) Selected genes from clusters located at chr12q14 and chr12q24 (displayed with arrowheads on the chr12 cartoon on the left) that were identified as differentially expressed amongst crisis fibroblasts by RNA-seq were investigated by RT-PCR to validate reported expression changes. RNA was extracted from a replica fibroblast sample set modelling the early and late crisis phases. Comparable early and late empty-vector (NEO) control RNA samples were also prepared, as defined in Supplementary Figure S1A. RNA-seq DESeq2 normalized data for the four fibroblast models at Early (grey) and Late (black) crisis time points is displayed as bar charts relating to individual genes of interest on the left-hand side with SD and statistical significance assessed by two-tailed paired *t*-test. RT-PCR data (with 95% CI) generated using the NEO control and E6E7 crisis fibroblast samples is displayed in the bar charts on the right, with the amplification of the listed target gene normalized to the amplification of *YWHAZ* that was constitutively expressed in these samples. Amplified cDNA levels were determined by calculating background-corrected gene density using ThermoFisher MyImageAnalysis software. Two replica experiments were performed using each of the four fibroblast line NEO and E6E7 matched samples. Statistical significance was assessed by repeated measures one-way ANOVA with Tukey's multiple comparison post-test (*P< 0.05, **P< 0.01, ***P< 0.001). (**B**)(i) Illustrative western blot image displaying detection of DTX1 67 kDa protein in whole cell extracts derived from human fibroblast Late (senescent) NEO (N) and Deep/Late E6E7 (E) crisis cells. MW markers are indicated on the left of the gel and a 42 kDa actin protein (housekeeping gene) western blot for the same samples is shown in the bottom panel to demonstrate sample loading. (ii) Quantification of actin-normalized DTX1 protein expression derived from three replica western blots using human fibroblast Late NEO (N) and Deep/Late E6E7 (E) crisis cells. Error bars show means and 95% CI and statistical significance of differential expression was examined by two-tailed paired *t*-test.

Chromosome 12 demonstrated exceptional frequency of telomere fusions (Figure 3B) and association with CNA (Supplementary Figure S7B), but was not overly represented in the group of genes most differentially expressed during crisis (Supplementary Figure S8F). We distinguished clusters of established fusion junctions (Supplementary Figure S9A) along chr12q that were dissimilar to the uniform spread of the simulated events (Supplementary Figure S9B) and used these to guide our interrogation of the RNA-seq dataset for chr12q genes deregulated by telomere crisis (Supplementary Table S3). Archetypes of pertinent signalling pathways that might prove pivotal for crisis transition were selected for expression analysis (Figure 6). Of these, only the expression patterns of *WIF1* (62) and *DTX1* (63,64) (regulators of Wnt and Notch signalling, respectively) were reproduced by RT-PCR assays, both demonstrating increased mRNA levels in the transformed fibroblasts at the late crisis stage that, importantly, were manifestly higher than those measured in the Early (proliferating) and Late (senescent) NEO control fibroblasts (Supplementary Figure S1A). By RT-PCR, *WIF1* was 3.5-fold increased (*P*<0.0001) between early- and late-crisis E6E7 cells (compared with 99.5-fold by RNA-seq; *P* = NS) and 5.24-fold (*P* < 0.0001) upregulated in the late-stage E6E7 compared with the NEO cells. Similarly, *DTX1* mRNA levels were 2.7-fold higher (*P* = 0.0023) in late- than early-crisis E6E7 cells (compared with 1.92-fold by RNA-seq; *P* = NS) and incalculable in comparison with the NEO cells for which transcripts were barely detectable.

The discrepant expression of *WIF1* and *DTX1* in E6E7-transformed fibroblasts transiting crisis and their NEO counterparts that do not undergo telomere-driven recombinations (Figure 6A) led us to question the involvement of Wnt and Notch signalling in these divergent cellular states. Furthermore, we discovered the fusion of *DTX1* intron 1 with the chr17p subtelomere in the late crisis IMR90 fibroblasts, potentially linking the activation of transcription at this locus with the occurrence of DNA damage that can precipitate telomere fusion. Despite measuring a dichotomy in transcript levels, we were unable to reliably establish differential expression of WIF1 protein between E6E7 and NEO fibroblast pairs. Moreover, culturing these cells in the presence of exogenous WIF1 (since it is a secreted mediator) had no discernible effect on growth or DNA repair of TALEN-induced DSB (data not shown). In contrast, DTX1 protein was reproducibly recognized by western blotting, although contrary to expectations, in greater abundance in senescent NEO controls than the crisis fibroblasts (Figure 6Bi). Quantification of multiple analyses indicated a 2.7-fold (*P* = 0.0065) higher DTX1 expression in the NEO cells (Figure 6Bii), compared with the reduced levels of mRNA transcripts detected in these samples by RT-PCR (Figure 6A and Supplementary Figure S9Ci). DTX1 shares ∼65% identity (at the amino acid level) with its paralogue, DTX4, which has been associated with DNA damage signalling in aging cells (65). To determine whether we were measuring DTX4 protein in dominance to DTX1 by western blotting, we looked to distinguish *DTX1* (Supplementary Figure S9Ci) from *DTX4* (Supplementary Figure S9Cii) mRNA expression in our fibroblast models by RT-PCR. Indeed, we were able to corroborate our earlier assertions of *DTX1* upregulation in E6E7 cells at late crisis stage (Figure 6A), providing a credible interpretation of the discrepant protein data (Figure 6B). *DTX1* is located within 50 kb of the *OAS1*–*3* gene cluster featured in the top 50 genes regulated in crisis for MRC5 and WI38 fibroblasts (Supplementary Figure S8C and D). These genes are activated as part of the immune response to virus (66) (Supplementary Table S5) and, therefore, conceivably induced following cellular sensing of extranuclear DNA (65,67) during the exacerbated genomic instability of crisis, as well as later stages of senescence. *DTX1* transcription may, then, be a by-product of generalized locus activation and/or DSB (68).

Extended investigations of genes at the intersections of chr12q-telomere fusions (Supplementary Figure S9Di) and CNA recognized by single nuclei genome sequencing (Figure 4B) returned no consistent or statistically significant gene expression changes, demonstrating that these genes are not abnormally regulated at the population level (Supplementary Figure S9Dii). Analogously, experimental destabilization of chr12q achieved through transient transfection of primary fibroblasts with bespoke transcription activator-like effector nucleases (TALEN) targeting the chr12q subtelomere (data not shown) had little impact on progression through a telomere-driven crisis, despite inducing fusion of chr12q chromatids. Thus, whilst chr12q instability may be a particular consequence of telomere-driven crisis, it cannot readily be modelled by introducing localized DNA damage or by tracing the contribution of solitary recombinations.

## DISCUSSION

In this study, we have sought to trace genomic and transcription changes that accompany progression through a telomere-driven crisis preceding malignant transformation or terminal growth arrest. We have revealed features and foci of advancing genomic instability and identified a central role for gene transcription in facilitating the long-range telomere-genomic recombinations that contribute to the disordered cancer genome.

### Active transcription precipitates telomere fusions with diverse genomic loci

The principal ambition of this study was to ascertain whether genomic loci incorporated into telomere fusions are transcriptionally active, thus elucidating underlying mechanisms and potential consequences for the individual cell and the evolving population. We confirmed previous observations (27,28) of enrichment of telomere fusion junctions within genomic coding sequence, rather than inter-genic regions (Figure 3C and Supplementary Figure S6A). Whereas HDR dominates in coding sequence, the considerable burden of DSB engendered by telomere attrition and cycles of chromosome fusion and breakage (BFB) may saturate this repair (69), resulting in increased scavenging of non-homologous DSB by the end-agnostic CNHEJ pathway. We performed RNA-seq in parallel, sampling from the same crisis fibroblast populations to yield unprecedented insights into loci undergoing concurrent transcription and telomere fusion (Figure 3D; Supplementary Figures S6B and 8F). A supplementary layer of information highlighting gross chromosomal abnormalities was created through whole genome sequencing of single nuclei isolated from deep crisis fibroblasts undergoing telomere fusion (Figure 4).

A conspicuous connection between gene transcription and fusion was unveiled by these analyses, with mRNA transcripts being detected for more than 50% all fused genes at all time points throughout crisis (Figure 3C and Supplementary Figure S6A). This association was most compelling in the earliest phase of crisis, when over 80% fused genes were expressed. The subsequent dilution of this effect might result from overall increased abundance of telomere fusions, as well as more widespread genomic instability and ectopic DSB. Surprisingly, exons as well as introns were found recombined with telomeres, negating the premise of enhanced surveillance or insulation of coding sequence. Differential DNA repair processes and efficiency at active gene loci compared with heterochromatin have long been recognized (70). Similarly, the occurrence of DSB during transcription have been reported as both necessary (71,72) and non-toxic to cells since it facilitates chromatin unwinding for polymerase access without precipitating a γH2AX damage response (73). In a telomere-driven crisis, the normal spatial and temporal segregation of replication and transcription may be deranged as increased DNA damage stimulates major transcriptome changes in response and cell cycle progression is impaired by the significant challenges of unresolved DNA damage and collapsed replication forks (74). DNA repair is activated when steric or structural conflict between RNA polymerase and the DNA replisome uncouples leading and lagging strand DNA synthesis to expose single-strand DNA (ssDNA) (29,50). Transcriptional arrest is required for DSB resolution, expediting large-scale genome rearrangements if either endures (73,75). Crucially, the conversion of topoisomerase II (TOP2)-induced DSB to persistent lesions is doubled in frequency at transcribed loci (76). This reaffirms the heightened susceptibility of transcribed sequences to instability and provides explanation for the elevated frequency of capture within telomere fusions.

Consistently, we observed that the mean length of genes fused to telomeres during crisis significantly exceeded the genome average (Figure 3E), exposing a correlation between gene length and propensity to break and/or undergo recombination (74,77). This association was not merely a distortion of the fusion frequency as a consequence of the greater genomic space occupied by longer genes since there was no comparable enrichment in inter-genic loci that constitute more than 50% of the human genome. Furthermore, the effect was independent of fragile site coincidence even though fragile sites are renowned harbours of long genes (56,78) and the mean length of established fusions also significantly exceeded that of a simulated dataset produced using the same mapping pipeline (Figure 3E). Operationally, the replication of long genes is limited by their transcription that may be incomplete over more than one round of cell division (79). Where long genes are also poor in initiation sites, delayed replication in concert with the extended distance of fork travel renders these sites vulnerable to instability (78). Multiple unresolved lesions within long genes promotes clustering—particularly in G1 cell cycle phase when chromatin has higher mobility (80) and HDR repair of coding sequence is restricted (81). Amente *et al.* (82) recently reported coincident 8-oxoguanine oxidation and γH2AX DDR foci at long transcribed genes as a result of ssDNA exposure during transcription and replication. Analogous foci at telomeres during crisis (83) may provide a mode of interaction between dysfunctional telomeres and damaged genes, facilitating fusion through common employment of DNA ligases 1 (especially at the replication fork) and 3 in both 8-oxoguanine base excision repair and ANHEJ at sister chromatids. The intrinsically disordered domains of key effectors of transcription (including RNA polymerase II (84) and transcription factors such as ERα (85)) and DNA repair (such as the ANHEJ-mediator, polymerase theta (POLQ) (86)) instigate functional compartmentalization of the genome through liquid–liquid phase separation, establishing an additional mechanism that could appose telomeres with transcribed loci.

Templated INS were far more common (≥50% compared with <20% events) at junctions between genomic loci and telomeres than junctions between telomere or subtelomere sequence (Supplementary Figure S3D). Whilst DNA POLQ has been ascribed the capacity for synthesis-dependent templated INS in ANHEJ-mediated repair (45,87), the mechanism is less well-characterized for the CNHEJ-mediated repair required for these telomere fusions with diverse genomic loci (20,27). γH2AX-dependent CNHEJ introduces INS and short deletions following nucleosome repositioning at DSB (88) and longer-range inter-chromosomal recombinations may be subjected to more extensive enzymatic modification if synapsis is fragile or delayed (89). Incorporation of Okazaki fragments derived from stalled replication forks (37,90) may underlie the increased frequency of templated INS at genomic loci challenged by transcriptional as well as replication stress. Certainly, the extended lengths of the INS determined at all junctions over time in crisis (Supplementary Figure S3F) is coherent with intensified replication stress. In addition, the notable reduction in both frequency and length of INS in genomic fusions derived from fibroblasts deficient in DNA ligase 1 (Supplementary Figure S3K) supports the replicative origin of this feature, whereas the lesser contractions in fibroblasts with DNA ligase 4 mutations may reflect the suppression of CNHEJ-mediated repair. Enhanced incidence of MH usage at genomic fusion junctions with impaired functionality of either DNA ligase (Supplementary Figure S3J) attests to the altered dependency on ANHEJ or distinct MH-mediated repair in these cells. Uncoupled DNA strand replication within subtelomeric sequence conceivably also underpins the length asymmetry of fused sister chromatids detected at deep and late crisis stages (Figure 2Bi and Supplementary Figure S4) (27). In support of this, resolution of precise intra-chromosomal fusion junctions revealed a characteristic nadir in each subtelomere that was coincident or adjacent to LTR motifs (Supplementary Figure S4C). These repetitive sequences present replication challenges, as well as marking ancient sites of chromosomal breakage by retroviral INS. Conversely, the homogenous distribution of chr17p intra-chromosomal fusions derived from DNA ligase 1-deficient samples implicates this replicative ligase as a key constituent of this effect.

### Inter-relationship of telomere fusions and gross genomic vulnerabilities

Chromosome 12 was replete in sites of recombination with telomeres in our four fibroblast crisis models (Figure 3B; Supplementary Figure S5A and B) that coincided with larger-scale CNA (Supplementary Figure S7B), representing a particular focus of genome instability. Mitotic chromosome segregation errors triggering cycles of BFB and break-induced replication (54,91,92) generate somatic copy number changes in cancer that manifest as genomic structural variants. Excision and amplification of nuclear material and extra-chromosomal DNA can substantially augment copy number, as well as releasing genes from endogenous regulatory features (93). In our deep crisis fibroblasts, copy number gains were twice as frequent as losses (Figure 4C), indicating both a relative increase in substrate available for capture within telomere fusions and the potential upregulation of oncogenes.

Neochromosomes derived by chromothripsis of chr12 are recurrent features of liposarcomas (94,95), evidencing the inherent susceptibility of this chromosome for gross structural transformations. Duplication of this entire chromosome is patently compatible with cell viability and malignancy as trisomy 12 is also diagnosed in ∼20% cases of CLL (96). Opportunely, whilst we were preparing this manuscript, Dewhurst and Yao *et al.* (97) released pre-print data indicating a propensity for structural variation at chromosome 12p in MRC5 fibroblasts that escaped telomere-driven crisis, corroborating our observations of the excessive instability of this chromosome in four independent fibroblast lines. Selection for chr12 aberrations is associated with amplification of the *CDK4-MDM2* locus at chr12q14.3 and *CCND2* at Chr12p13.32. Whilst we ascertained telomere fusions in our crisis fibroblasts (and in our former dataset of nuclease-induced telomere fusions (27)) at chr12q14.3 (Supplementary Figure S9A), intersections with CNA occurred only for the telomere-proximal chr12q24 chromosome band. Furthermore, we did not observe enhanced expression of these candidate oncogenes and, instead, measured heightened transcription of the intervening *WIF1* gene (Figure 6A) that is associated with suppression, rather than support of oncogenic signalling (98). Instability and CNA at the terminus of chr12q may result from continuous BFB of the telomere eroded to within the fusogenic length range (99), promoting both long and short-range intra-chromosomal fusions that may facilitate escape from crisis (20). Indeed, chr12q telomere fusions were readily amplified from our crisis fibroblast samples (data not shown) that could contribute to the destabilization of this chromosome arm.

### The cellular stress responses that establish and sustain crisis provide substrates for telomere fusions

The global transcriptomic programs that impress the extensive phenotypic changes associated with telomere-driven crisis and senescence are coordinated by physical interactions between chromatin and the nucleolus (100,101) that may engender telomere fusions (102,103). In our fibroblast models, immortality was not achieved and the curtailment of proliferation in terminal crisis was accompanied by the enhanced expression of genes associated with the DDR and inflammaging (Supplementary Figure S8E, G and I). Heightened expression of *DTX1* (64) and *WIF1* (104) in late-crisis samples (Figure 6A) is also symptomatic of this ultimate growth arrest that may resemble immunological anergy (105). Accordingly, whilst *WIF1* is frequently silenced in cancer, indolent CLL cells only rarely display *WIF1* methylation (106) and trisomy chr12 CLL (96) is particularly associated with an anergic state that can be subverted to provoke cellular reactivation and apoptosis (105). A resurgence of *DTX1* transcription may coincide with renewed stimulation of the DDR *OAS1–3* (107) gene cluster (Supplementary Figure S8F and Table S3) within 50 kb of this gene, as well as functionally promoting terminal crisis concurrent with upregulation of *IL6*, *CDKN1A*and *PTSG2* (10) (Figure 5C and D; Supplementary Figure S8G).

Regulation of inter-chromosomal interactions is essential for the integration and systematization of cellular responses to diverse challenges (108). Pertinently, we identified mutual transcription factor regulatory networks associated with both genes incorporated into telomere fusions and genes differentially expressed during crisis (Supplementary Figure S8H). Nuclear hormone receptors, exemplified by AR (androgen receptor) and ESR1 (oestrogen receptor 1) in both our datasets, are recognized mediators of long-range interactions (71,109) and epigenetic remodelling (110). Liganded ERα interplays with cohesin to instigate chromatin looping (111,112) and chromothripsis in breast cancer cell lines (113). Analogously, liganded AR expedites chromosomal translocations by nucleating distant loci that are ligated by NHEJ following targeted nuclease activity (109). Reciprocally regulated genes may, therefore, become juxtaposed and aberrantly recombined where DSB occur during a telomere-driven crisis. Notably, we resolved multiple telomere fusions with gene promoter and enhancer sequences (Supplementary Figure S6A), suggesting regulatory sequences may integrate mediators of transcription and DNA repair. In addition, gene density (Supplementary Figure S5C) and transcriptional output (Supplementary Figure S6B) were both associated with telomere fusion frequency, propelling concurrent evolution of the cancer transcriptome and rearranged genome.

## CONCLUSION

Tracking four human fibroblast models through telomere crisis has revealed global genome stability and transcriptome development in response to exacerbated DNA damage combined with persistent proliferation. Eroded telomeres fuse with genomic loci challenged by conflicts between replication and transcription, conceivably nucleated by the DNA binding factors that respond to cellular distress and DSB. Enduring genomic insult and telomere attrition ultimately drive a coordinated transition into terminal crisis, licenced by chromatin rearrangement and accompanied by upregulation of cellular stress response genes and apoptotic mediators. These studies expose a pivotal role for active transcription in precipitating long-range inter-chromosomal recombinations and identify commonly deregulated inflammatory modulators that may be strategically targeted to suppress tumour growth, clonal evolution and metastasis.

## DATA AVAILABILITY

Fusion-seq, RNA-seq and single nuclei WGA-seq sequence data have been submitted to the NCBI SRA database under the BioProject accession number PRJNA659174.

## Supplementary Material

zcaa044_Supplemental_Files
