# Supplementary material for: Tracking telomere fusions through crisis reveals conflict between DNA transcription and the DNA damage response
Source: NAR Cancer. 2021 Jan 6;3(1):zcaa044. doi: 10.1093/narcan/zcaa044 (PMC7787266; doi:10.1093/narcan/zcaa044)

Supplementary Figure 1

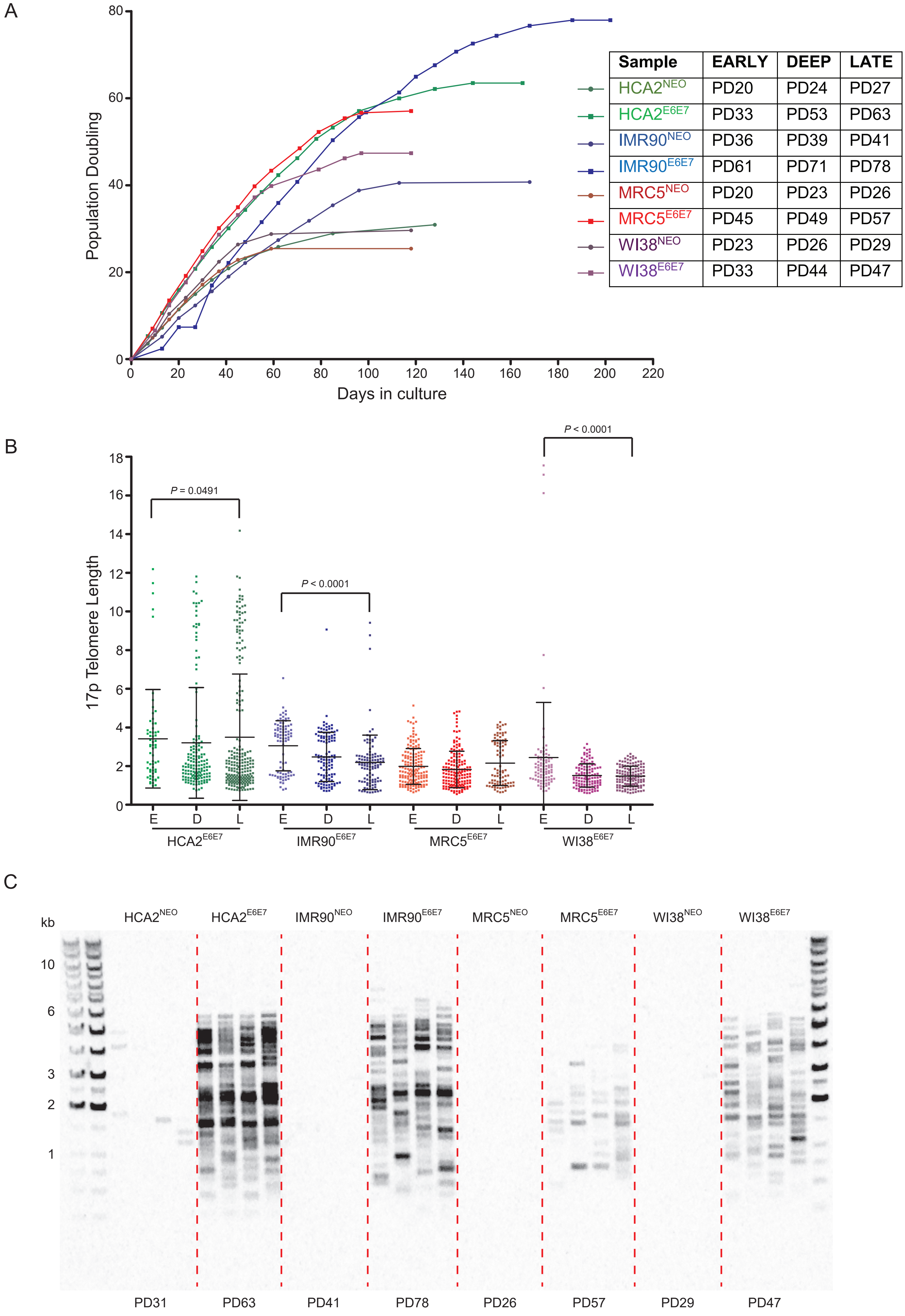

Sample	EARLY	DEEP	LATE
HCA2^NEO	PD20	PD24	PD27
HCA2^E6E7	PD33	PD53	PD63
IMR90^NEO	PD36	PD39	PD41
IMR90^E6E7	PD61	PD71	PD78
MRC5^NEO	PD20	PD23	PD26
MRC5^E6E7	PD45	PD49	PD57
WI38^NEO	PD23	PD26	PD29
WI38^E6E7	PD33	PD44	PD47

Supplementary Figure 2

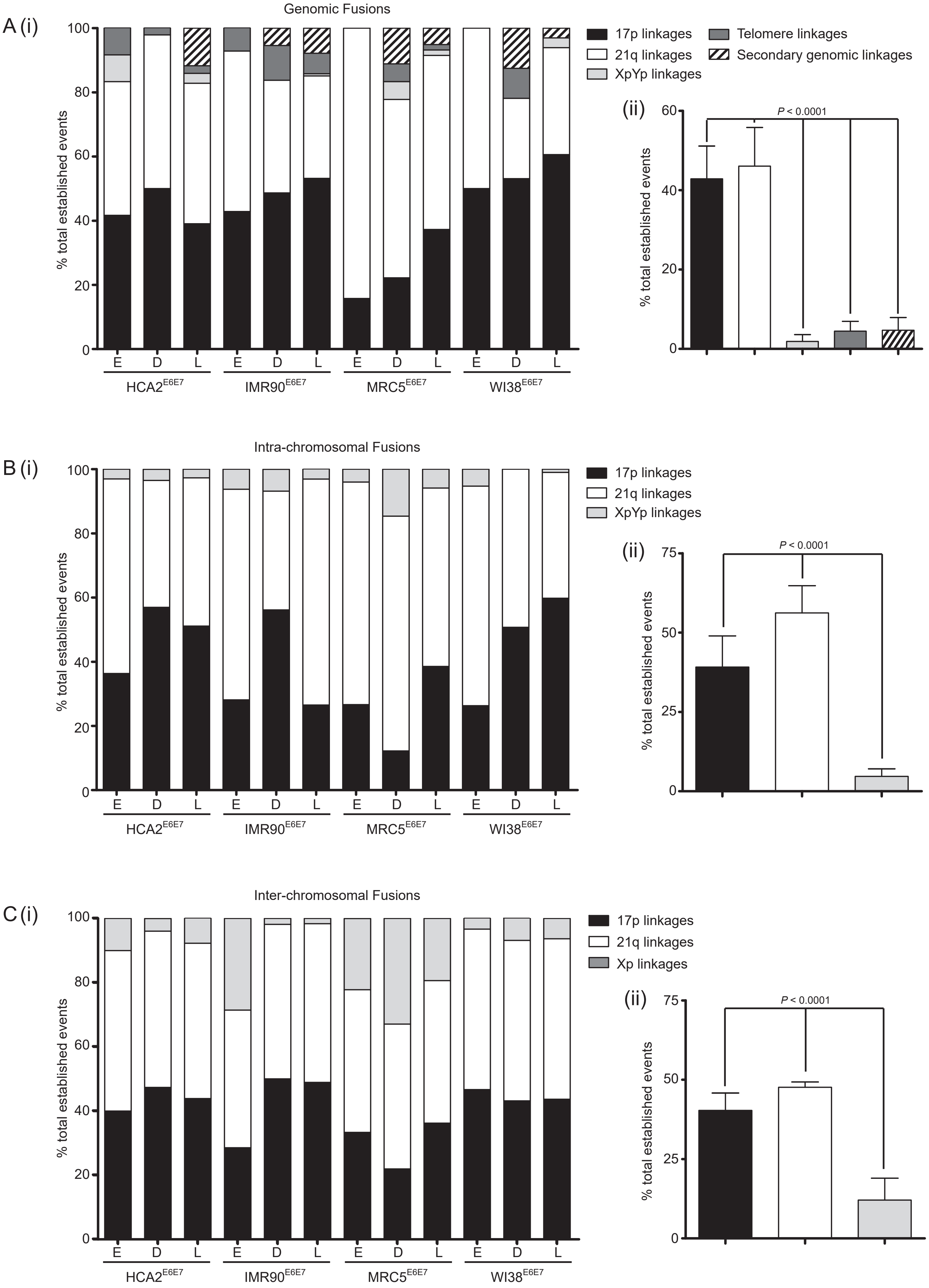

Supplementary Figure 3

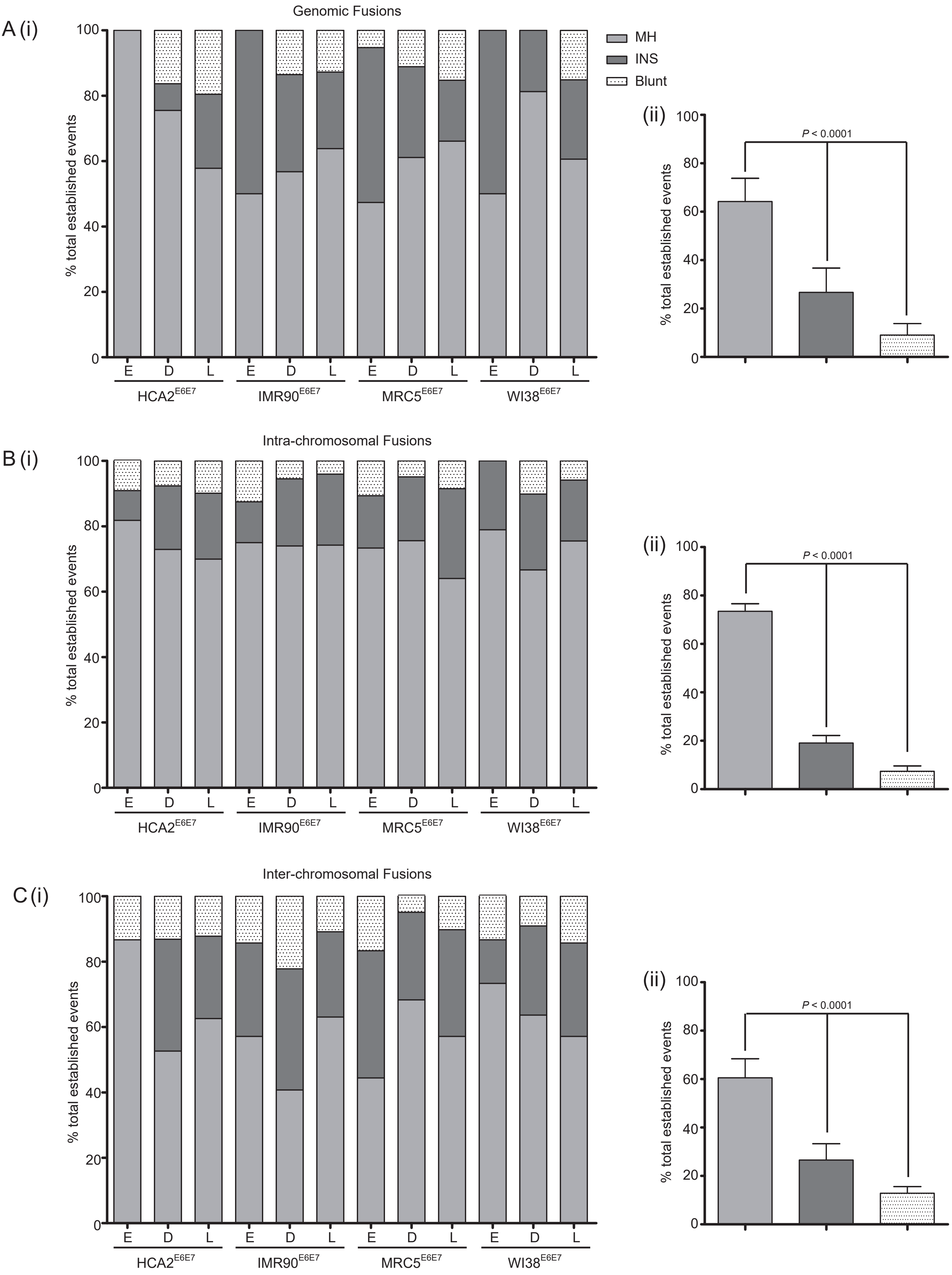

Supplementary Figure 3

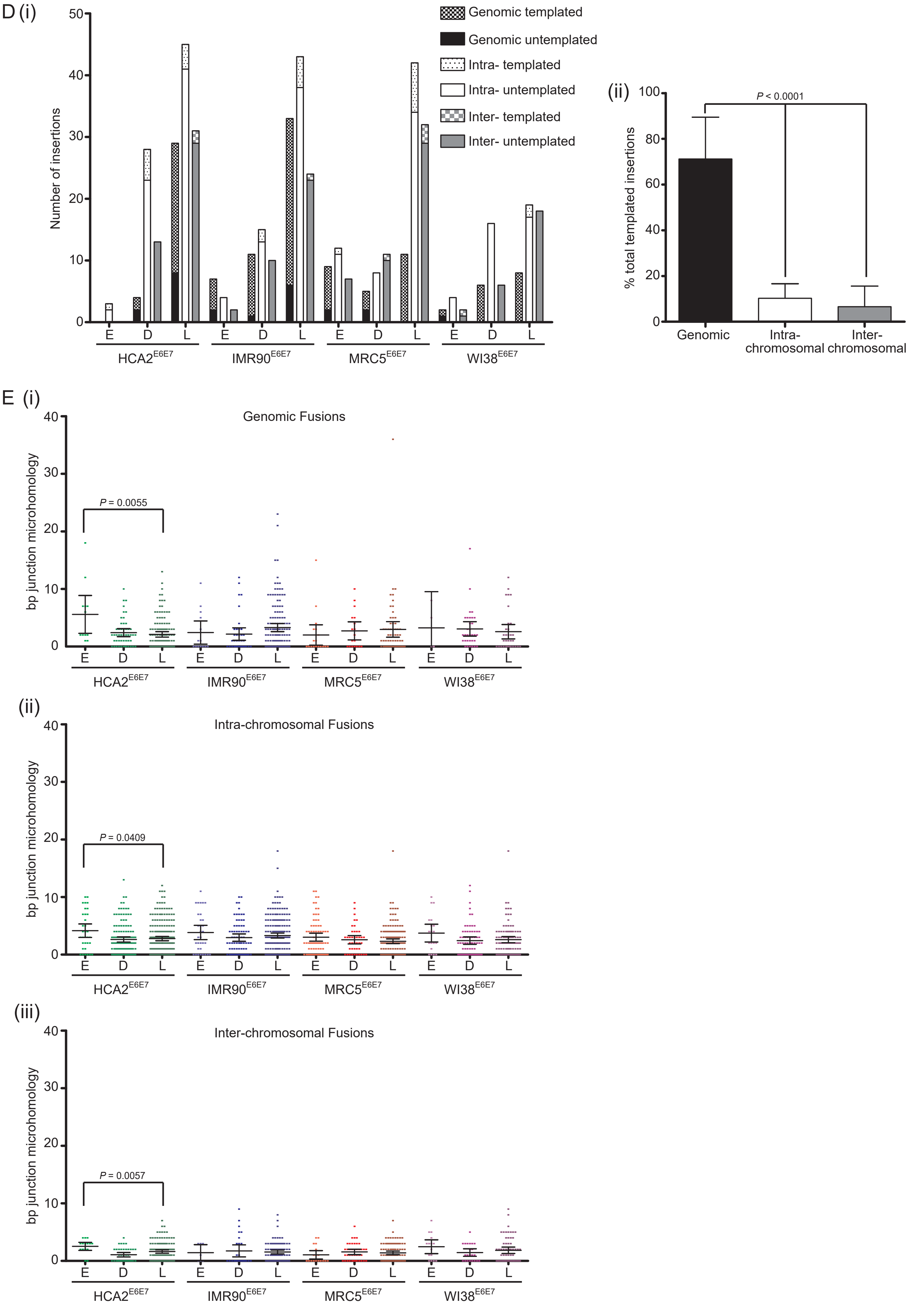

Figure 3

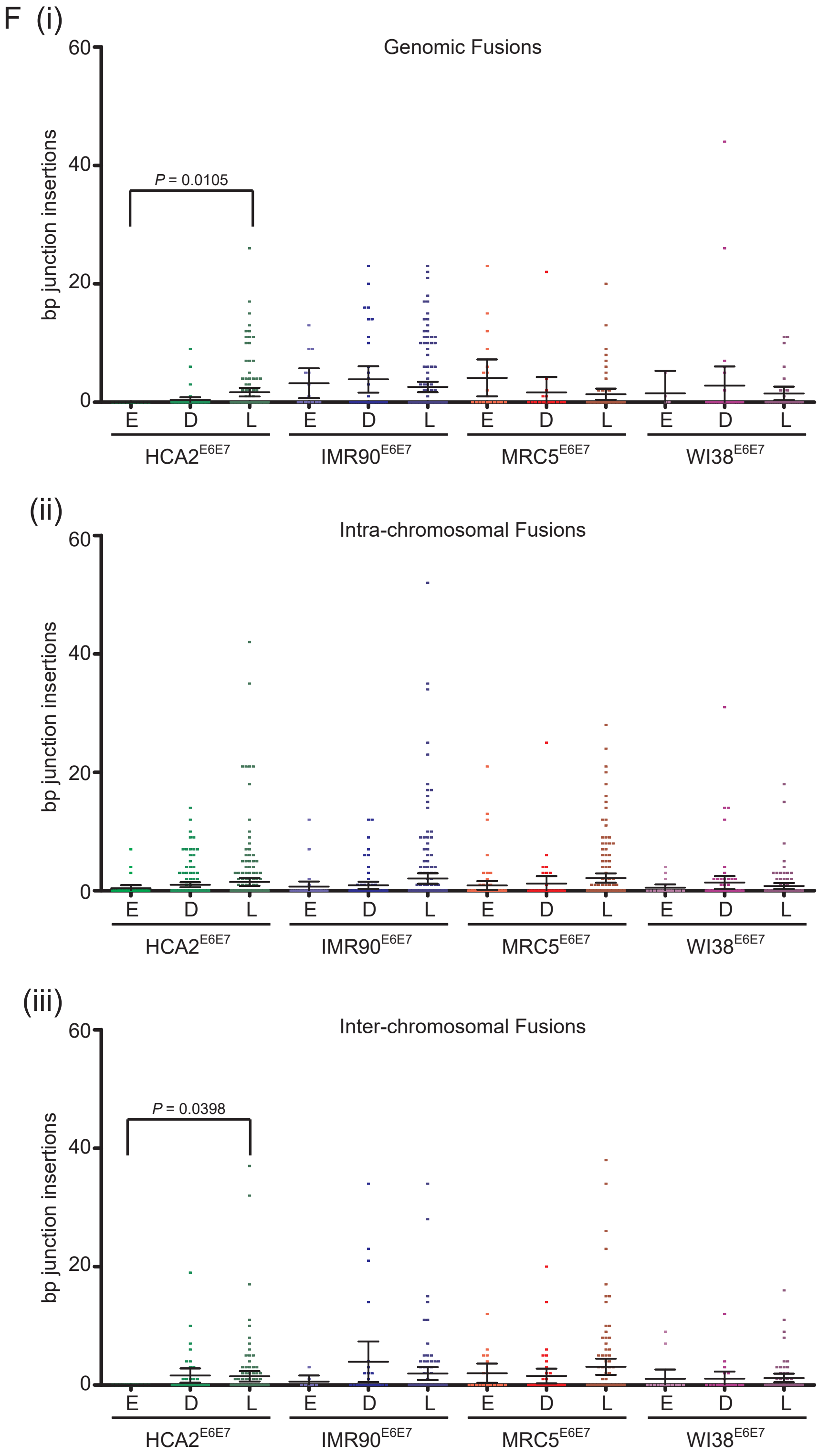
F (i)
Genomic Fusions
bp junction insertions
P = 0.0105
60
40
20
0
E D L E D L E D L E D L
HCA2E6E7
IMR90E6E7
MRC5E6E7
WI38E6E7
(ii)
Intra-chromosomal Fusions
bp junction insertions
60
40
20
0
E D L E D L E D L E D L
HCA2E6E7
IMR90E6E7
MRC5E6E7
WI38E6E7
(iii)
Inter-chromosomal Fusions
bp junction insertions
P = 0.0398
60
40
20
0
E D L E D L E D L E D L
HCA2E6E7
IMR90E6E7
MRC5E6E7
WI38E6E7

Supplementary Figure 3

G

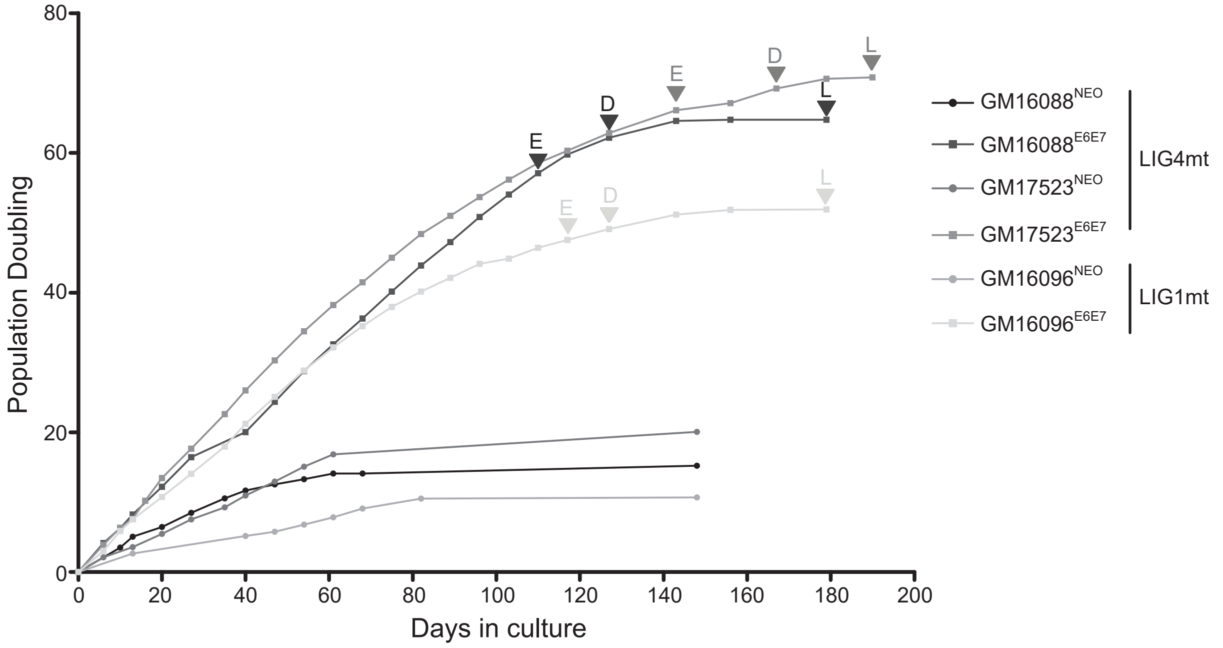

H

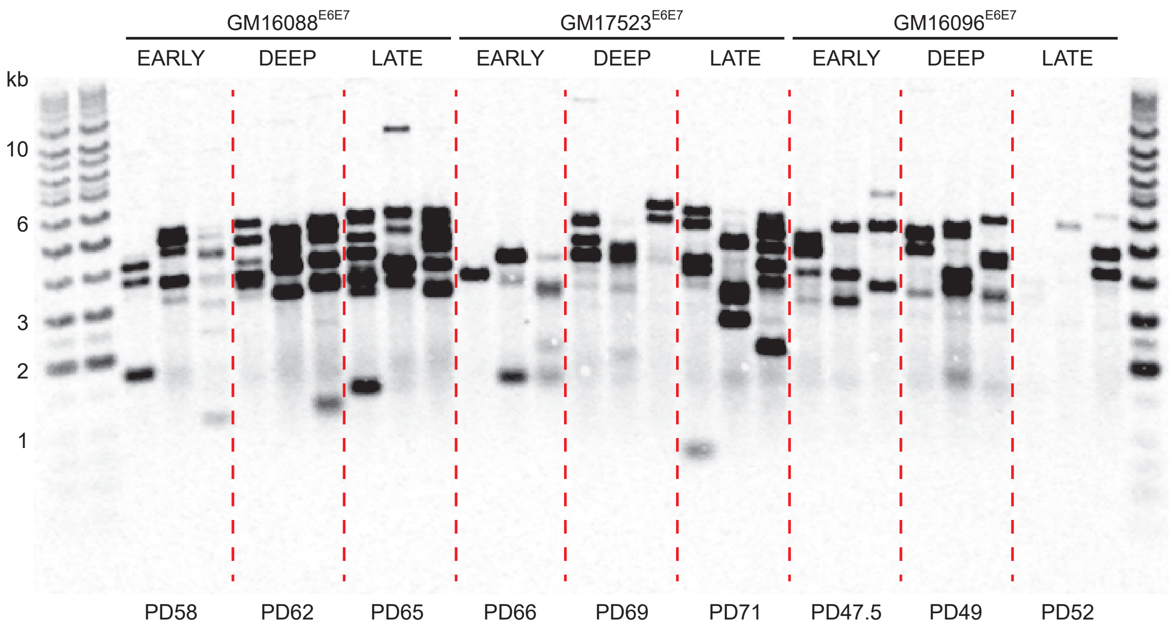

I

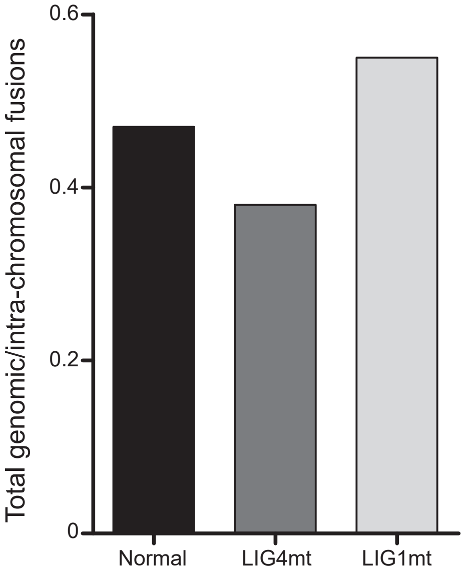

Supplementary Figure 3

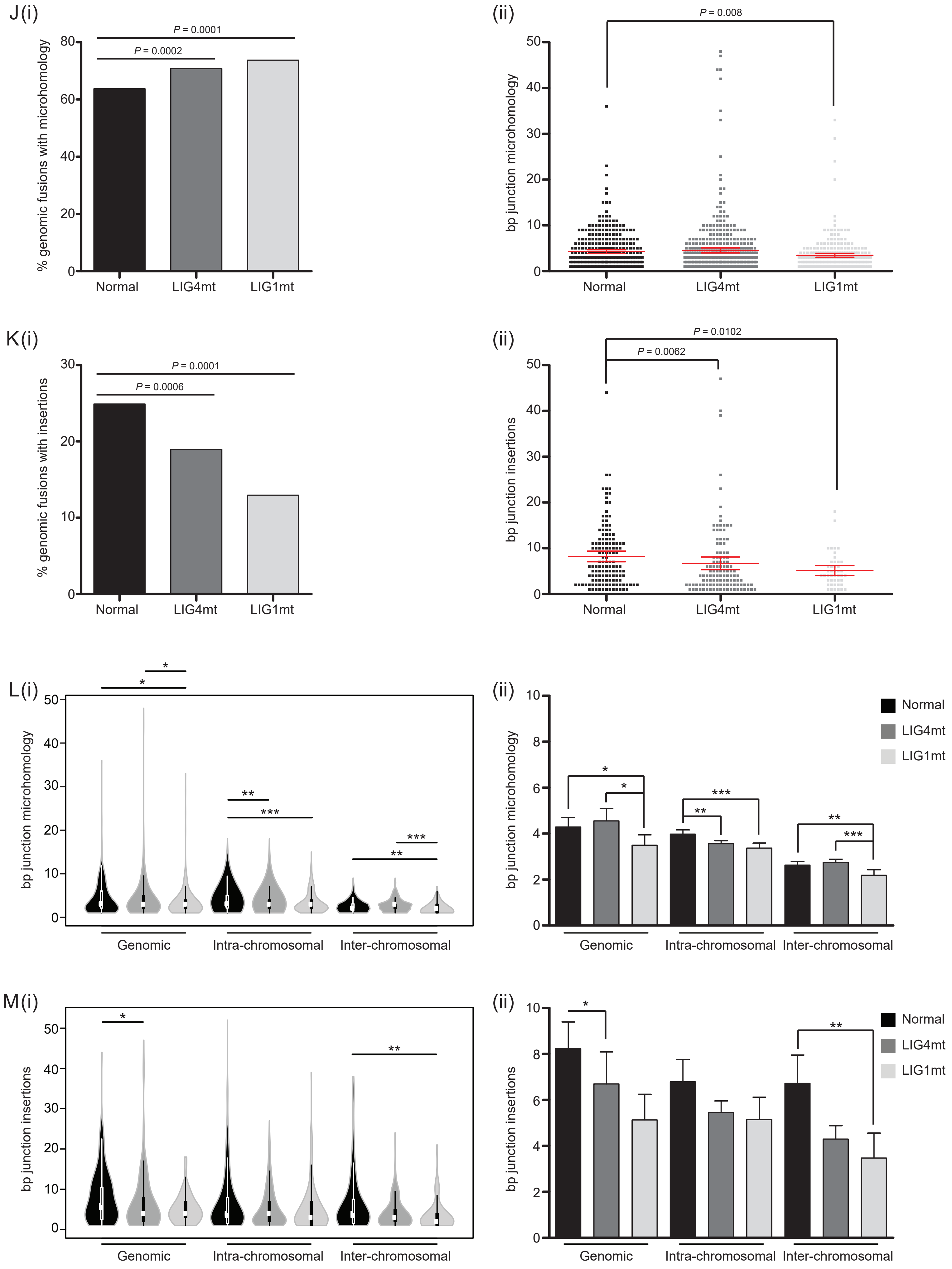

Supplementary Figure 4

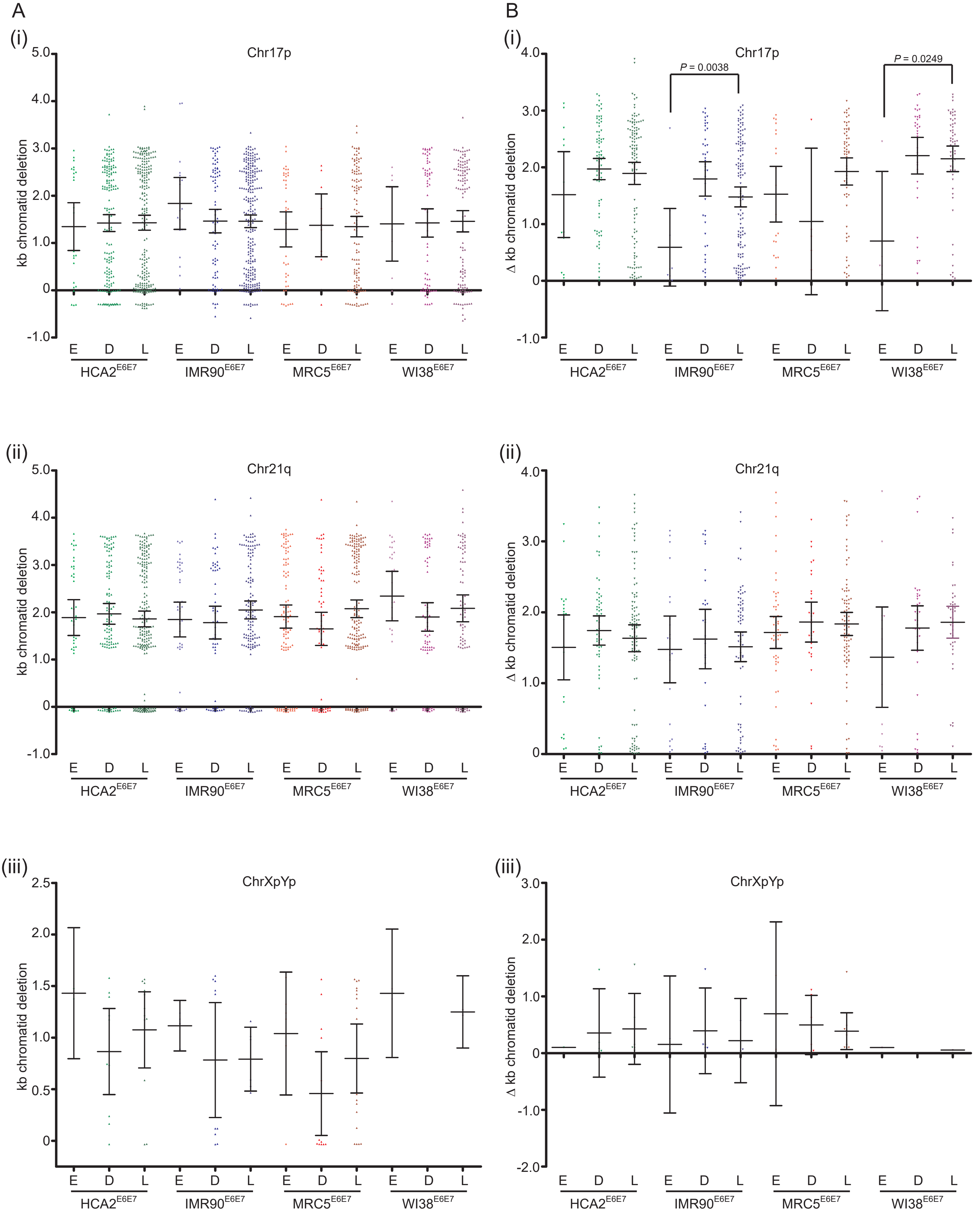

Supplementary Figure 4

C

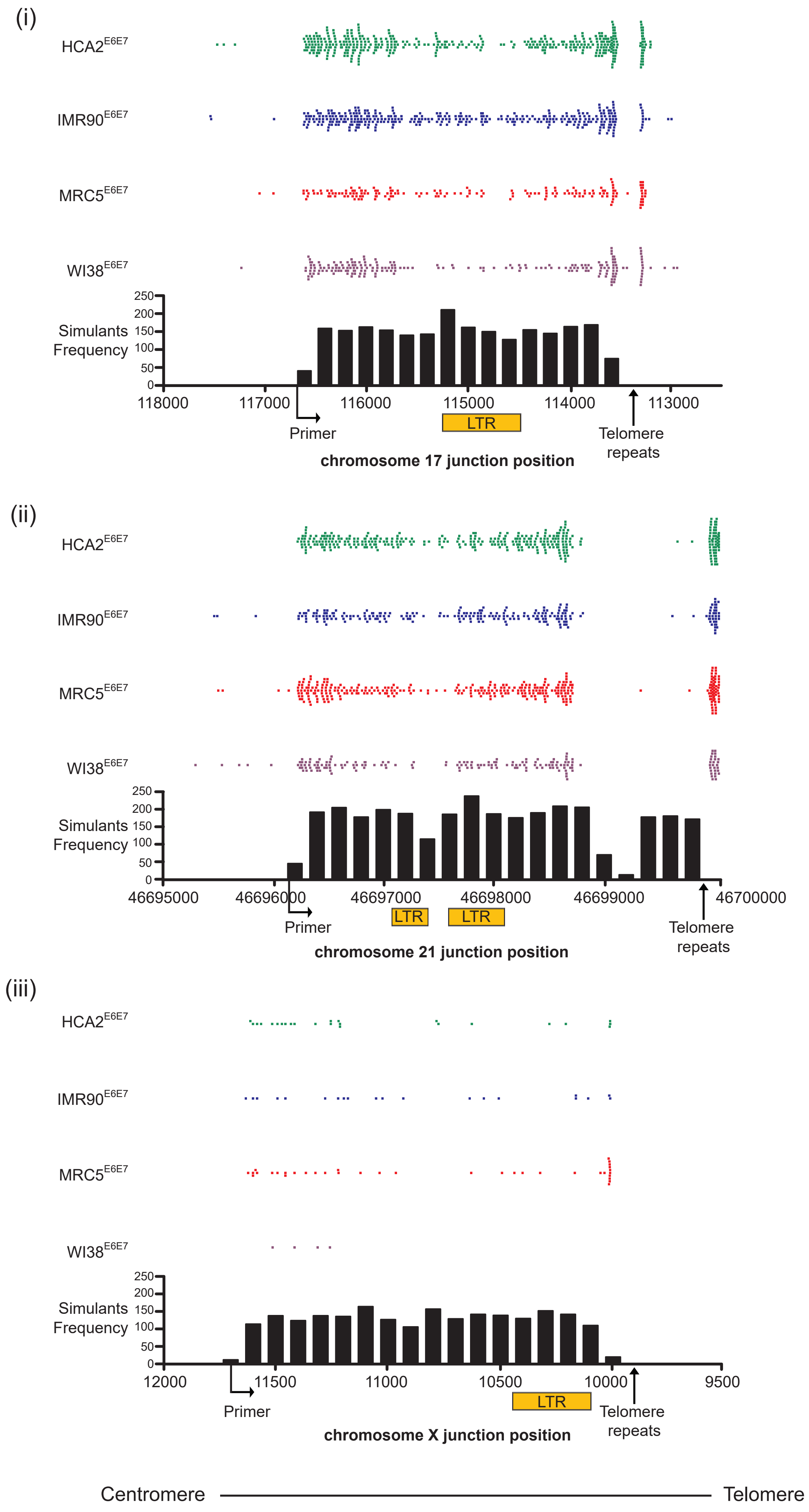

Supplementary Figure 4

D

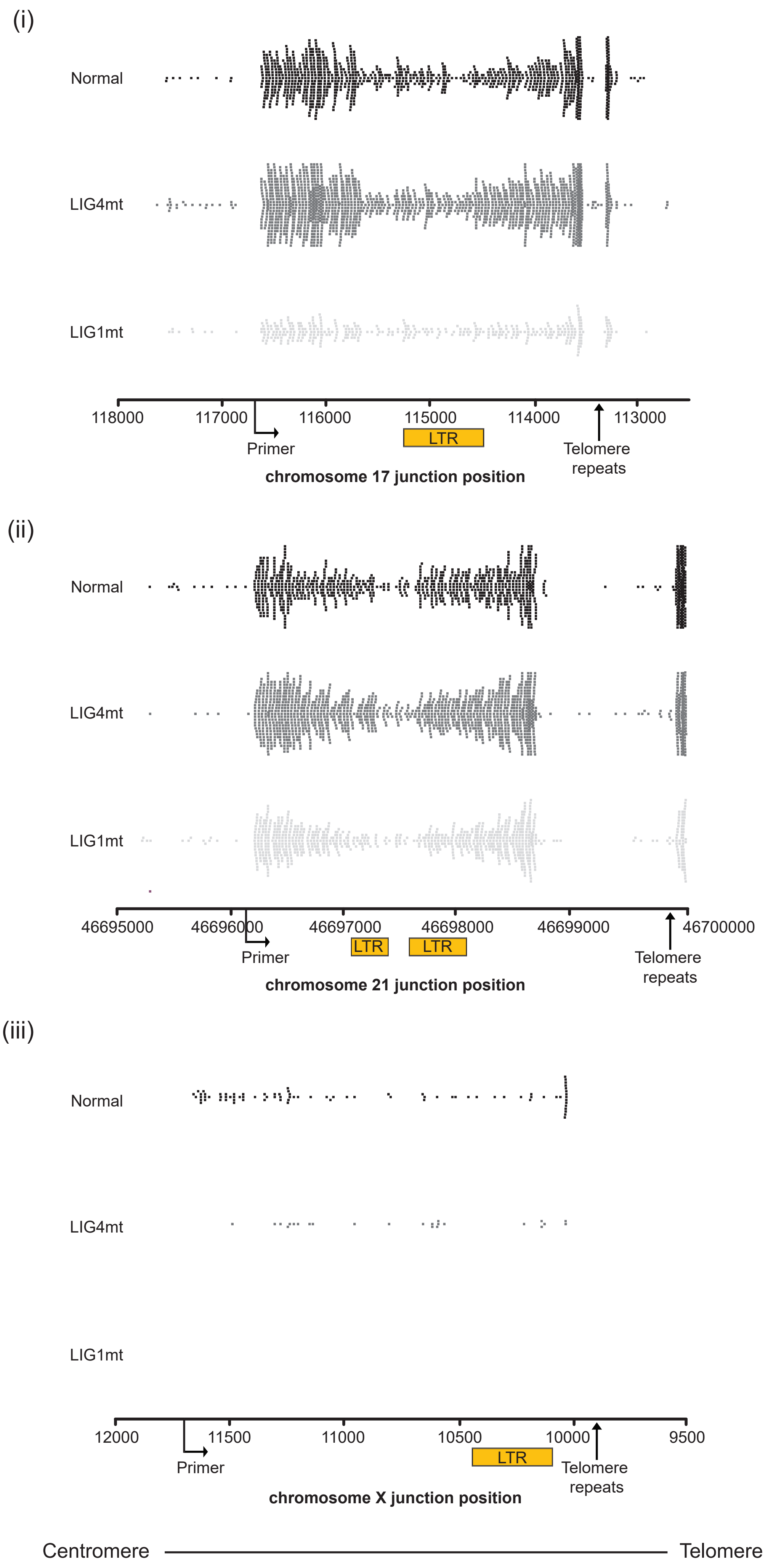

Supplementary Figure 4

E (i)

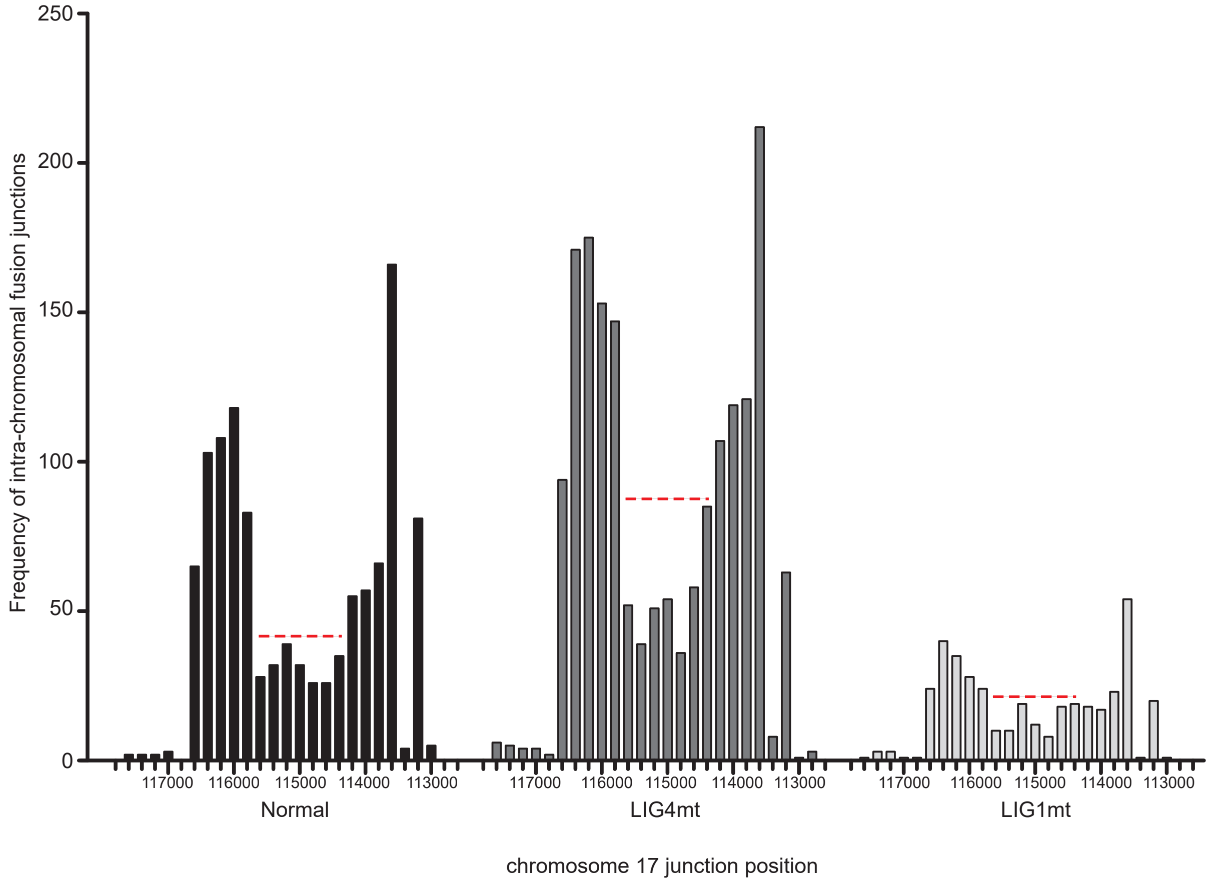

(ii)

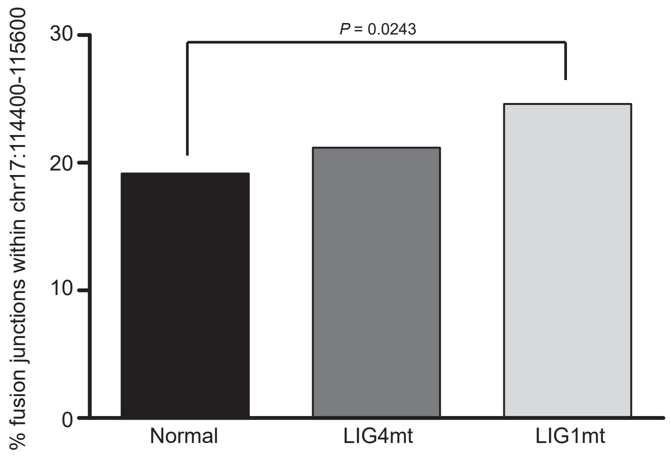

Supplementary Figure 5

A

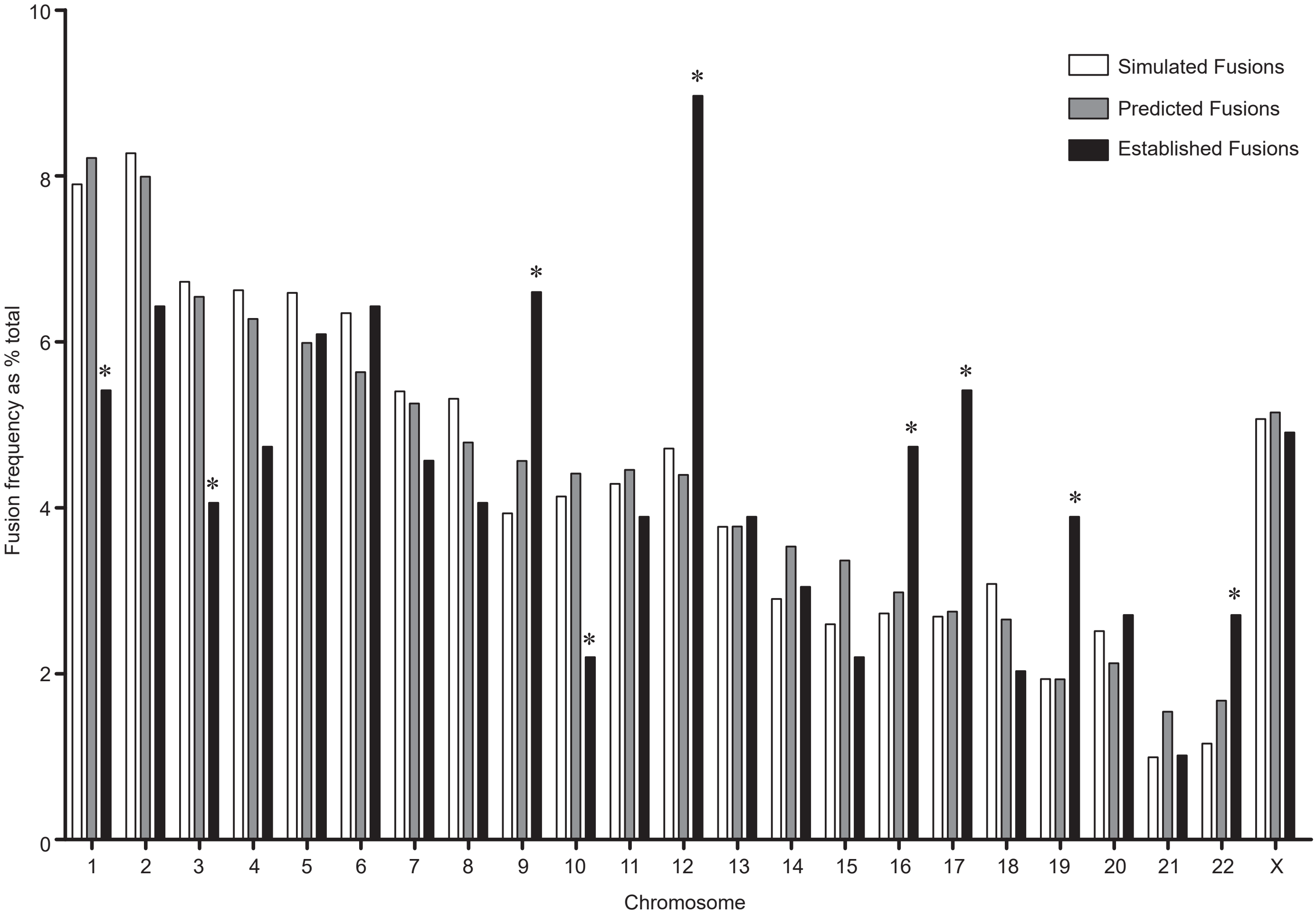

Supplementary Figure 5

B

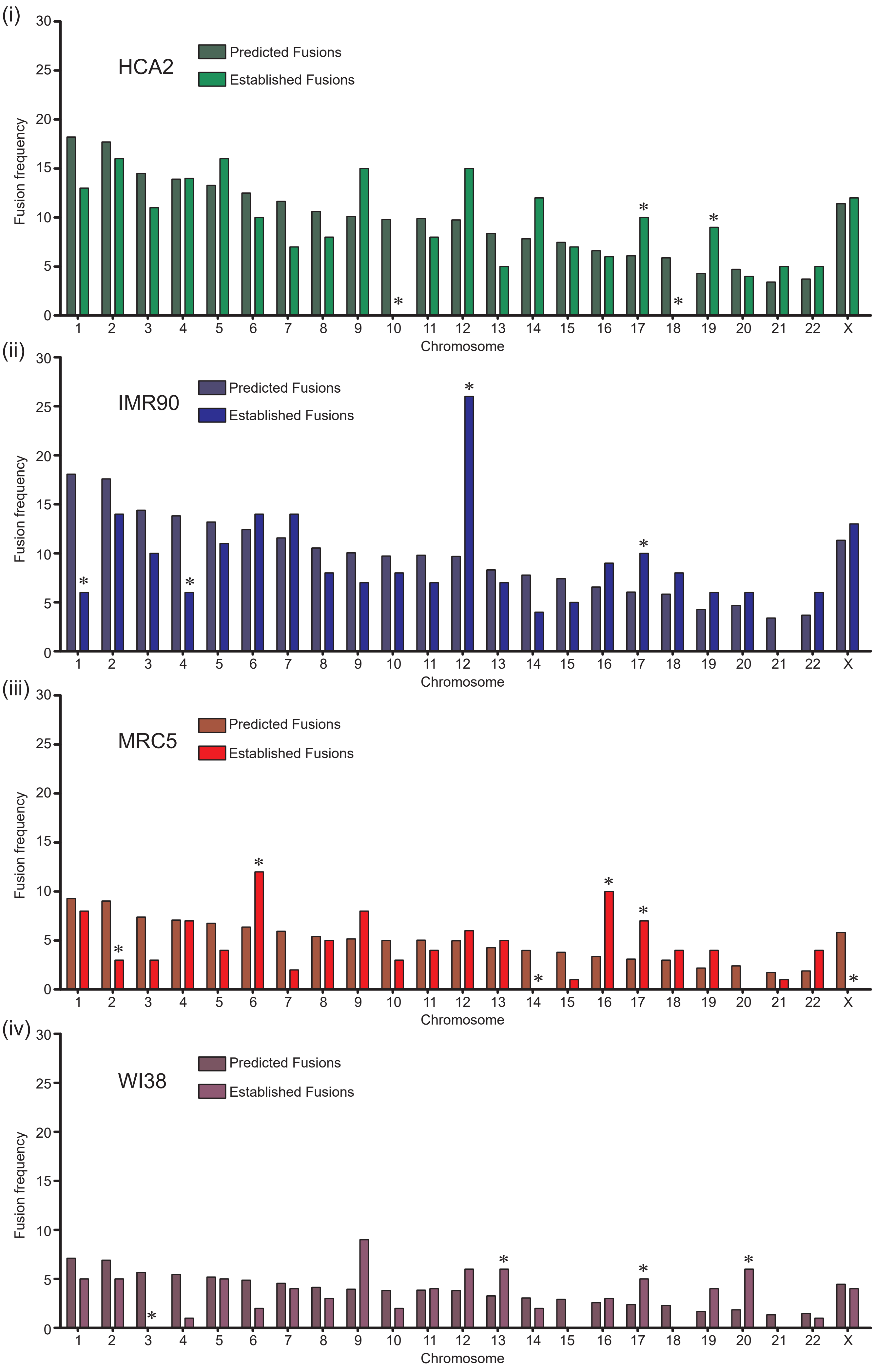

Supplementary Figure 5

C

(i)

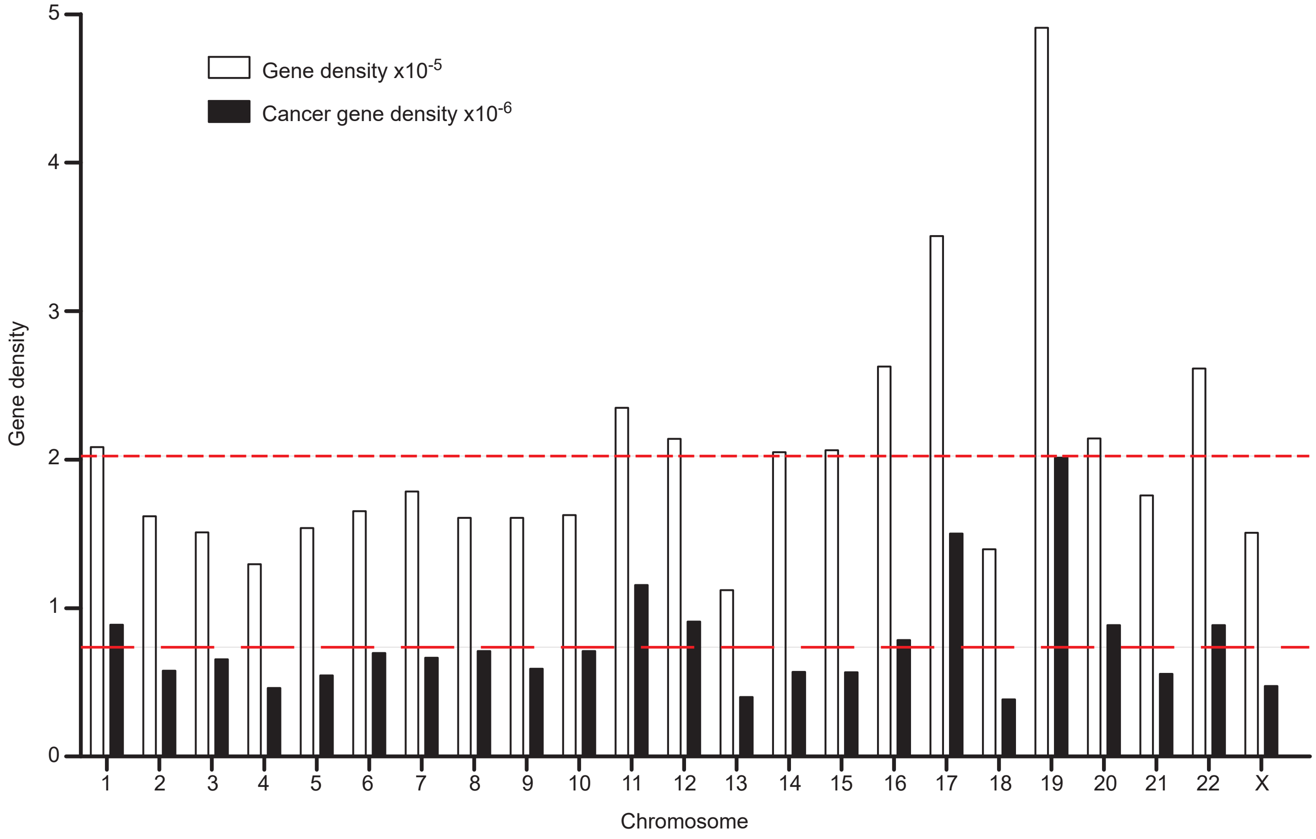

(ii)

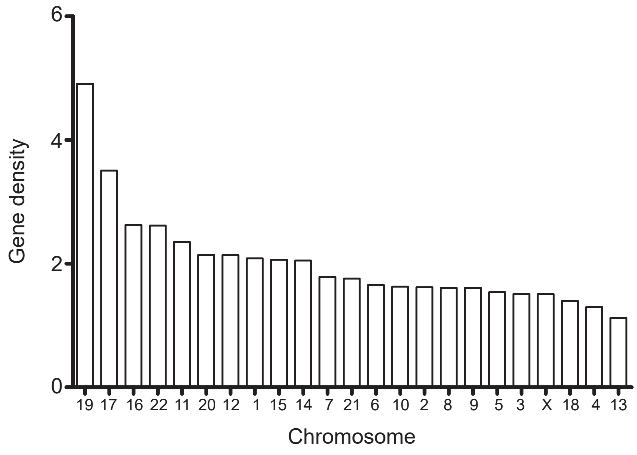

(ii)

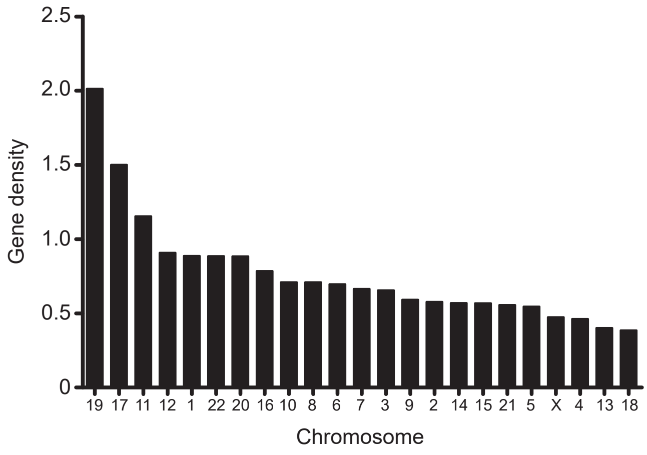

Supplementary Figure 6

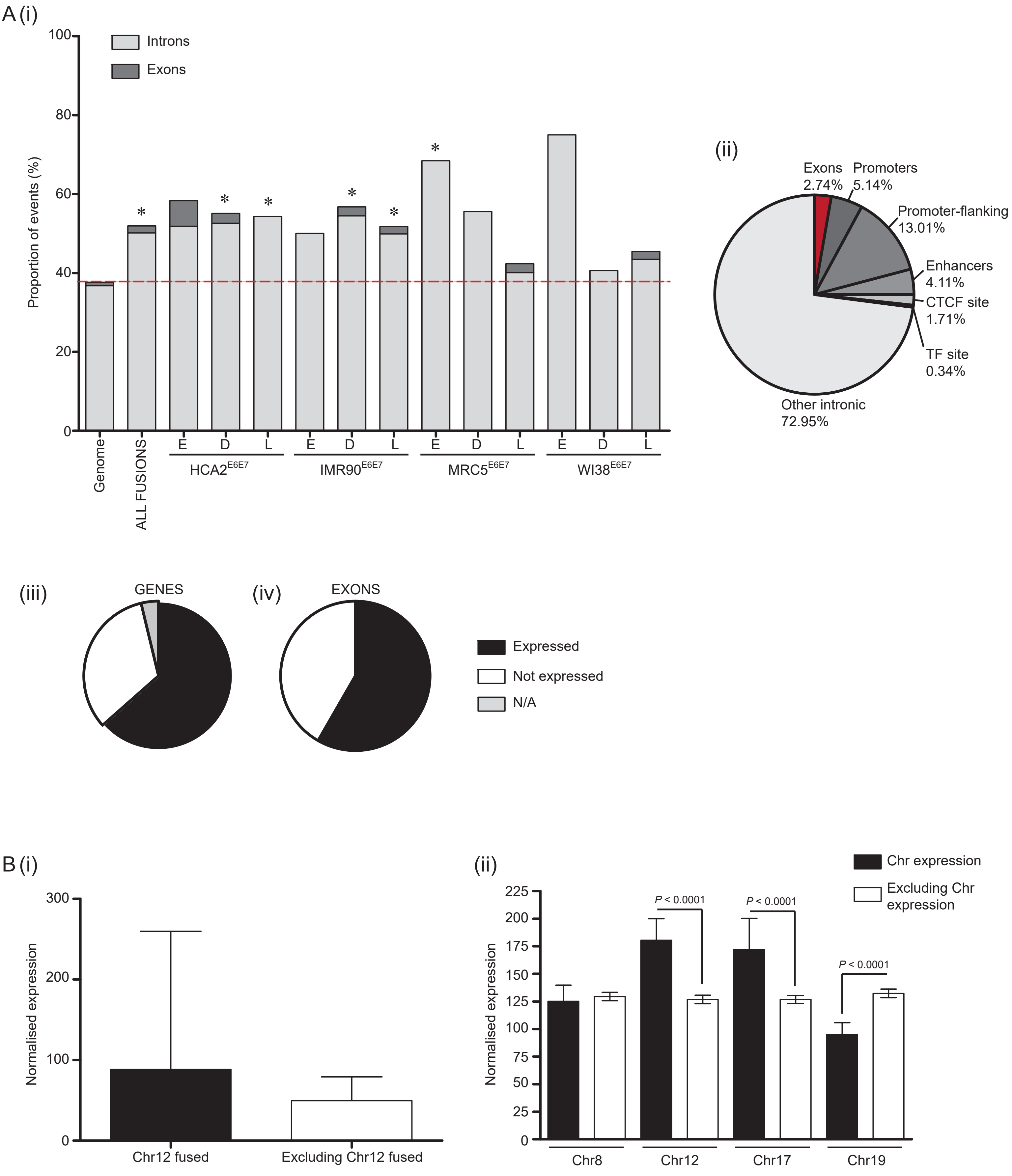

Supplementary Figure 6

C

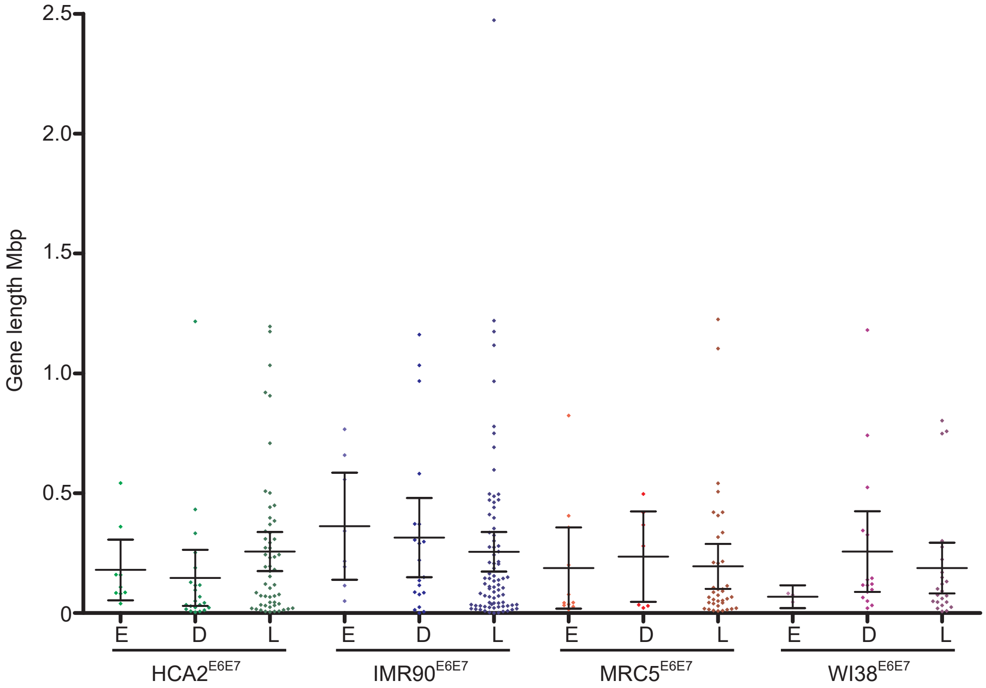

Supplementary Figure 6

D

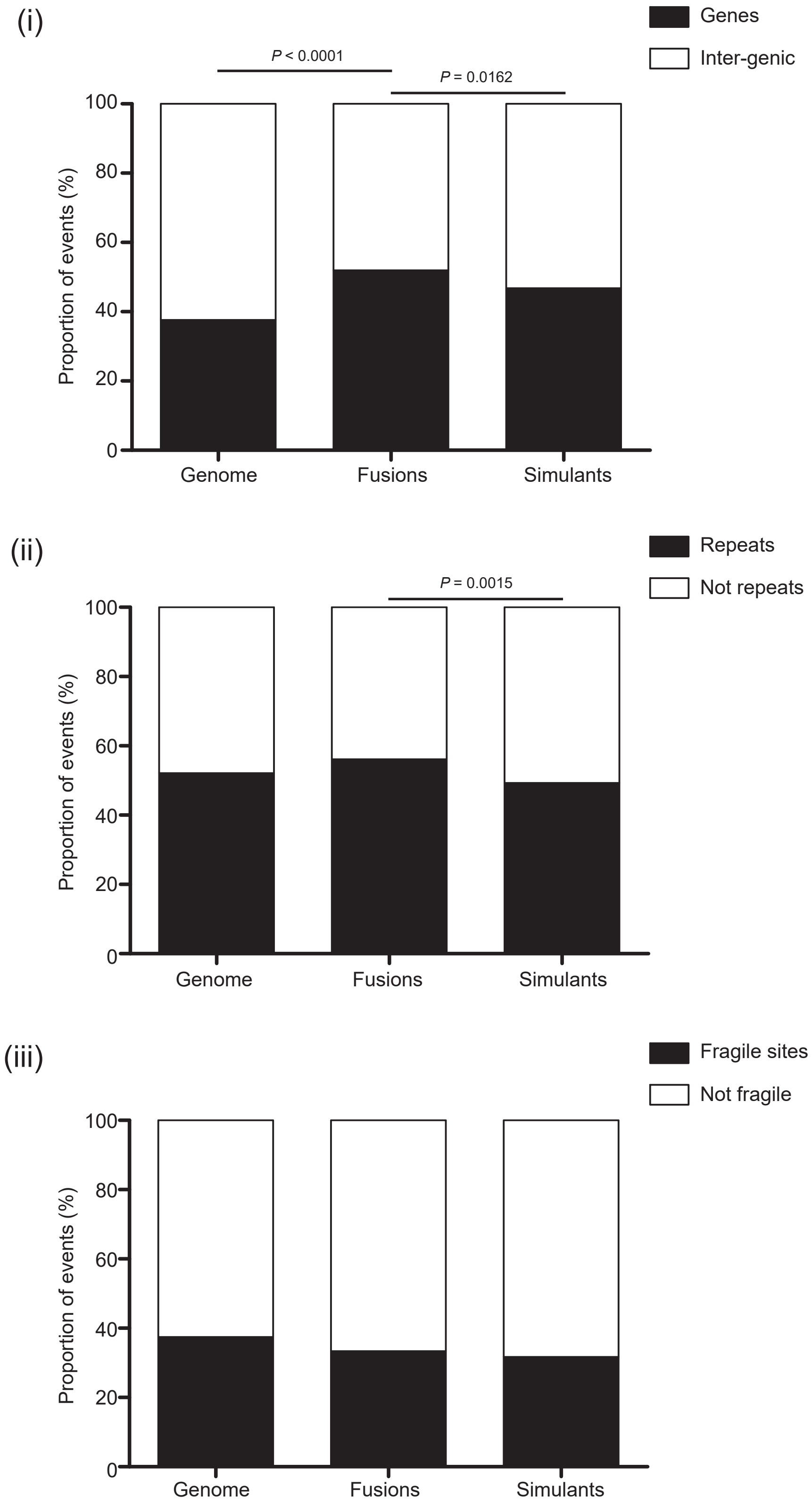

Supplementary Figure 7

A (i)

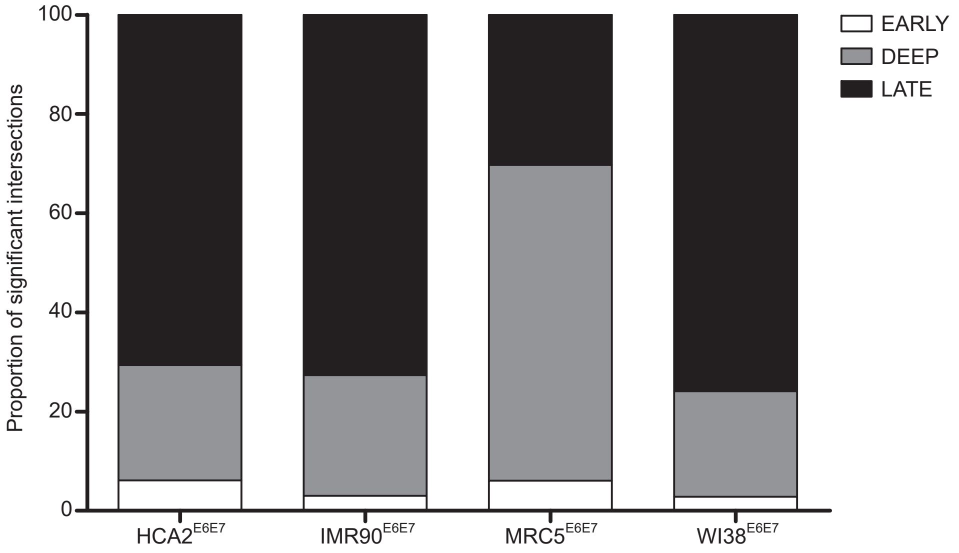

(ii)

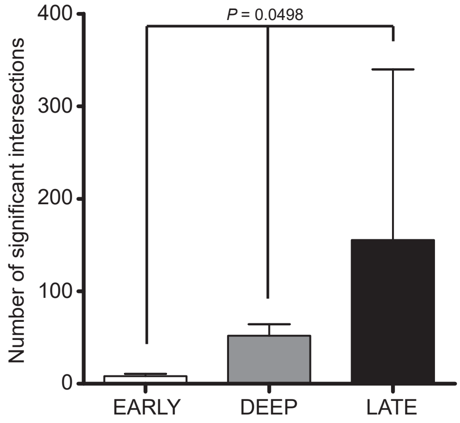

(iii)

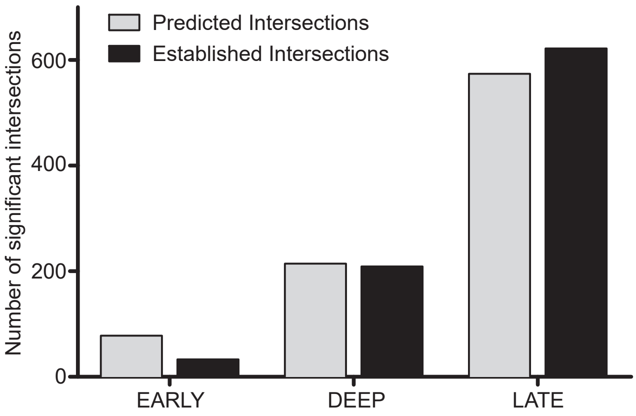

Supplementary Figure 7

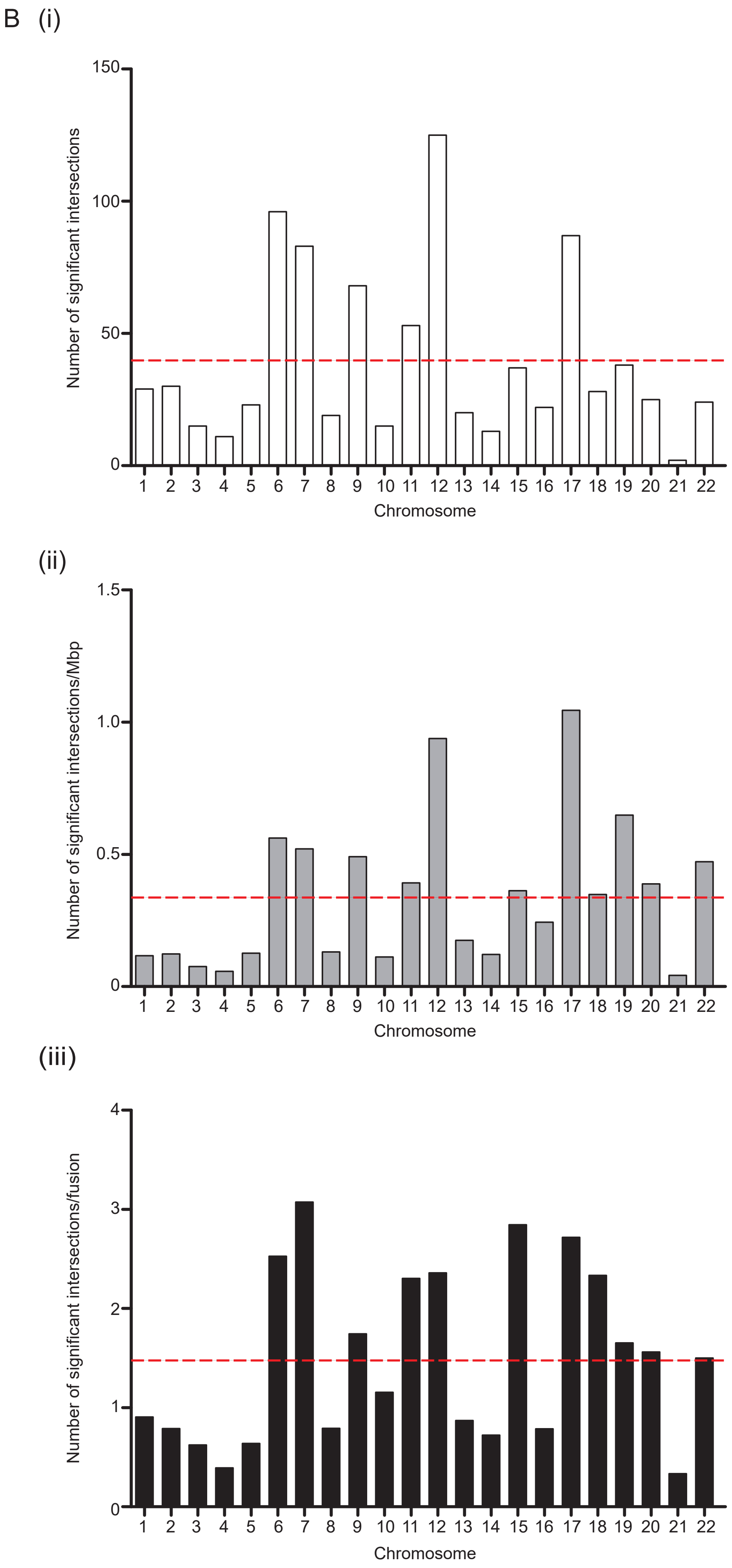

Supplementary Figure 8

A

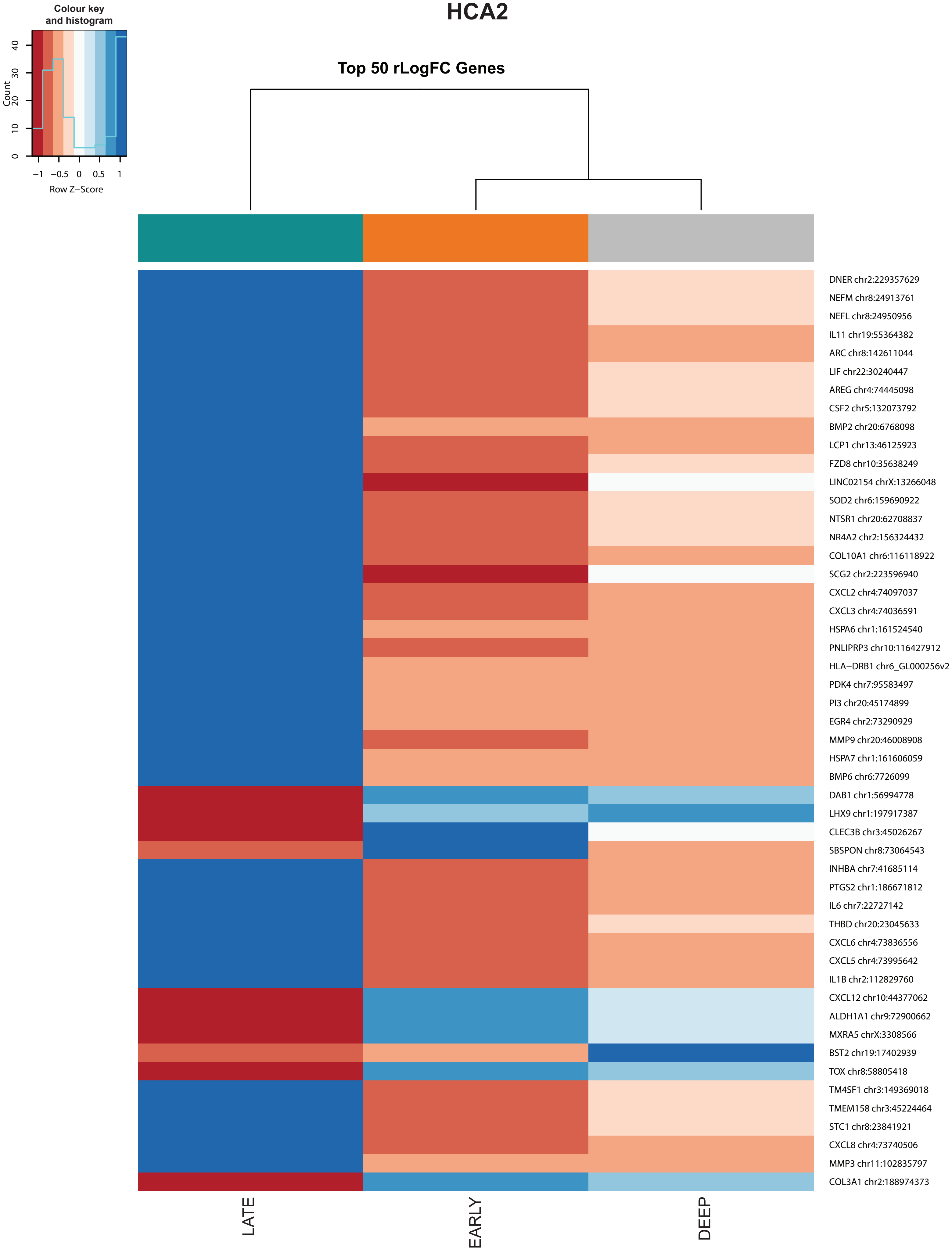

Supplementary Figure 8

B

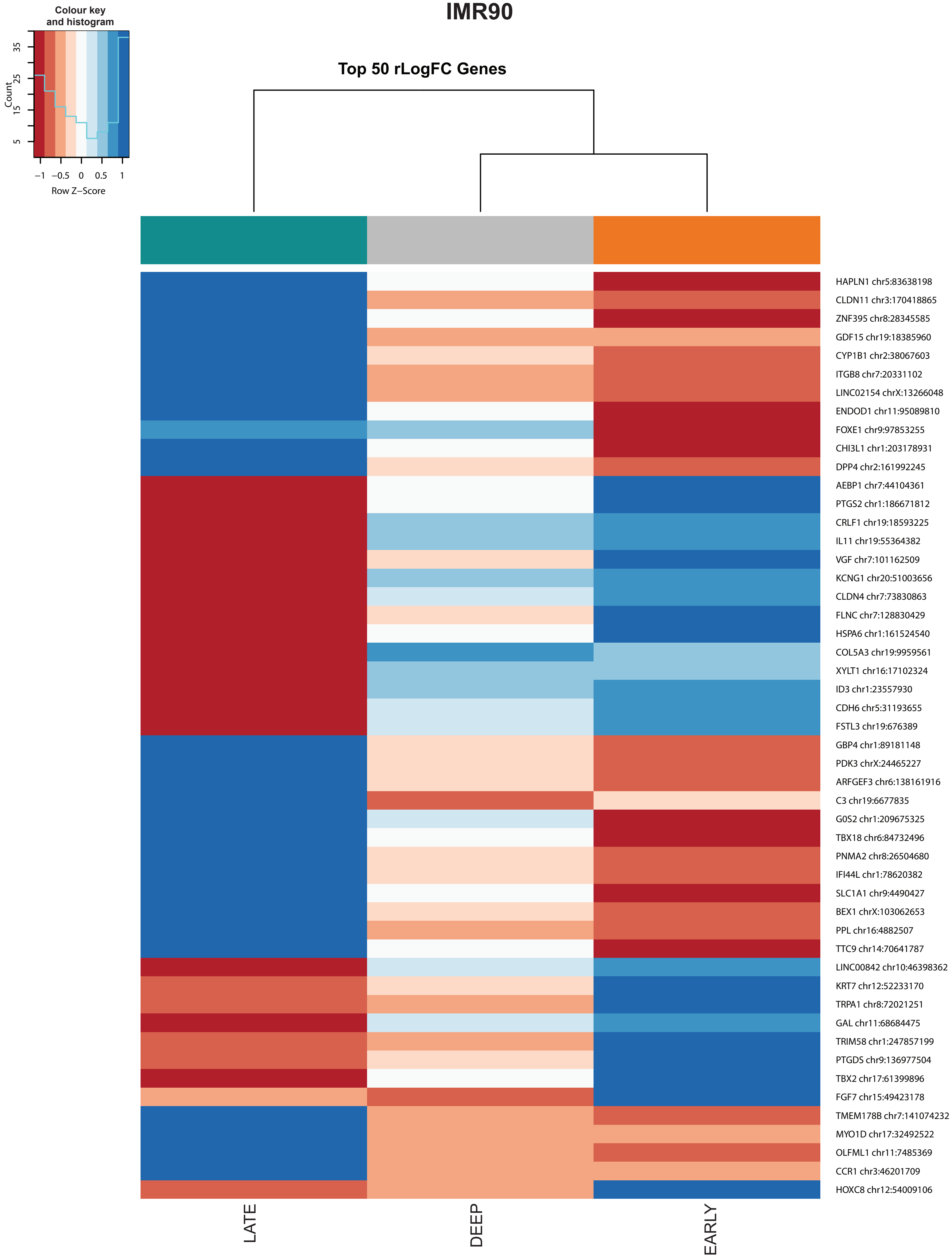

Supplementary Figure 8

C

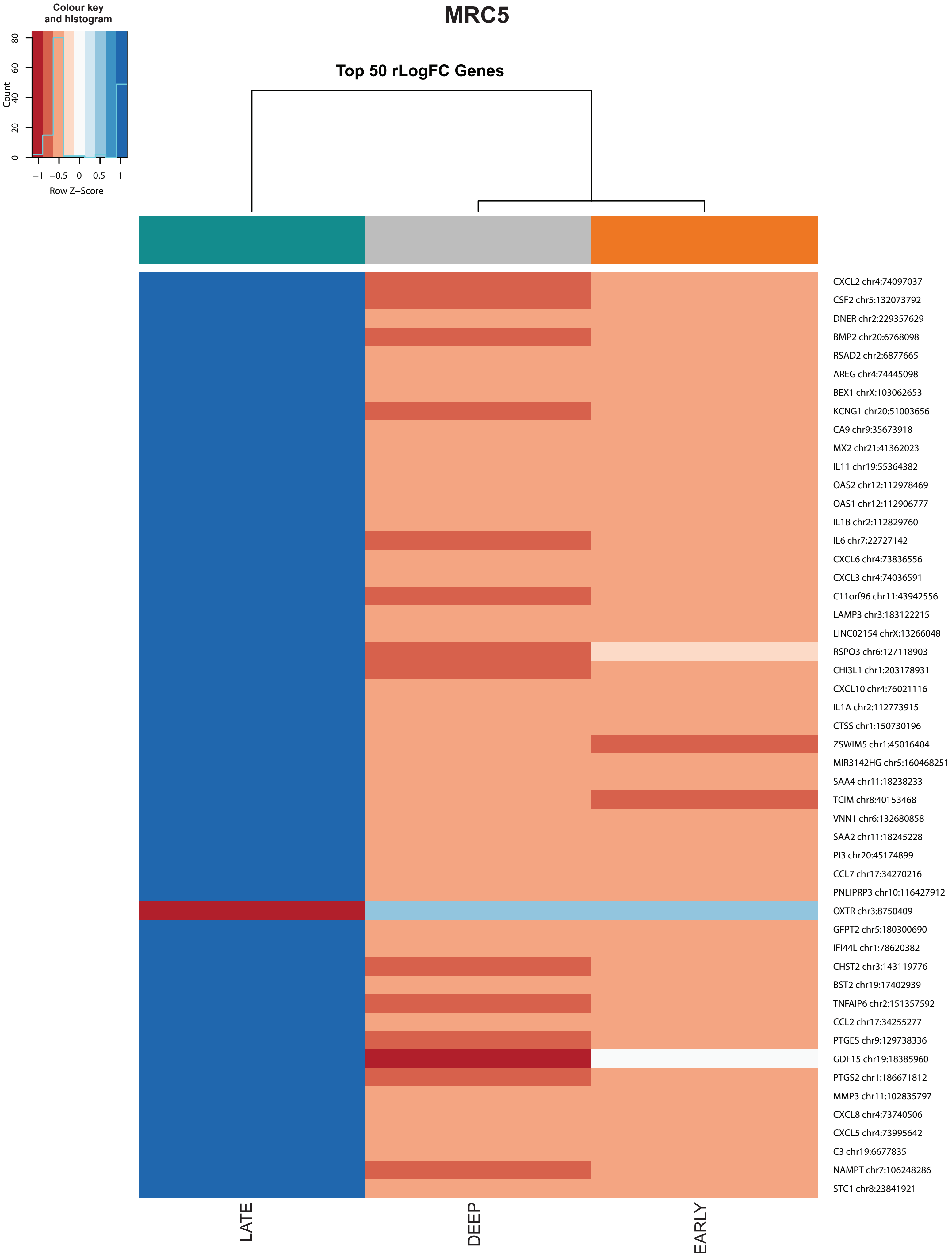

Supplementary Figure 8

D

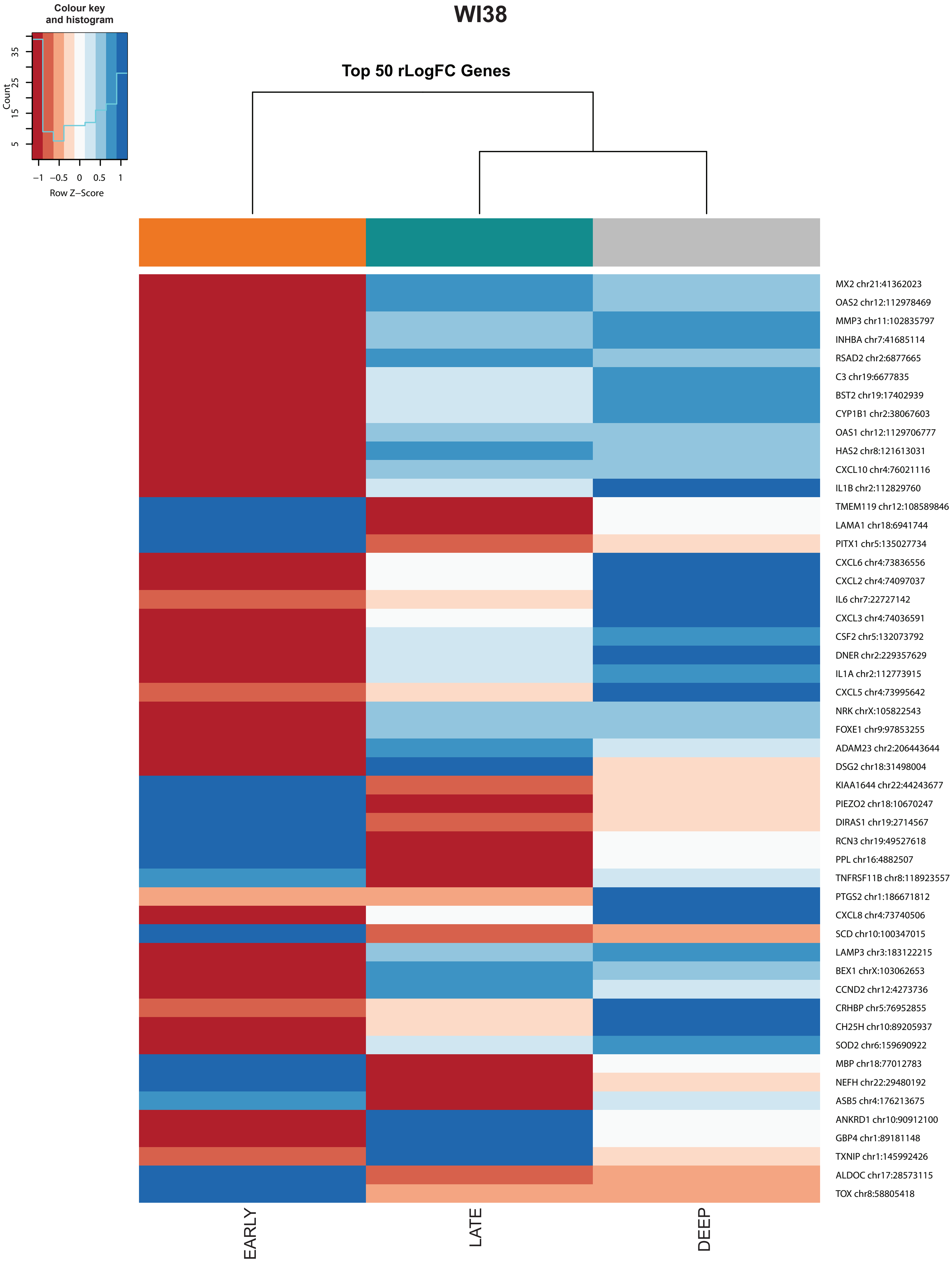

Supplementary Figure 8

E

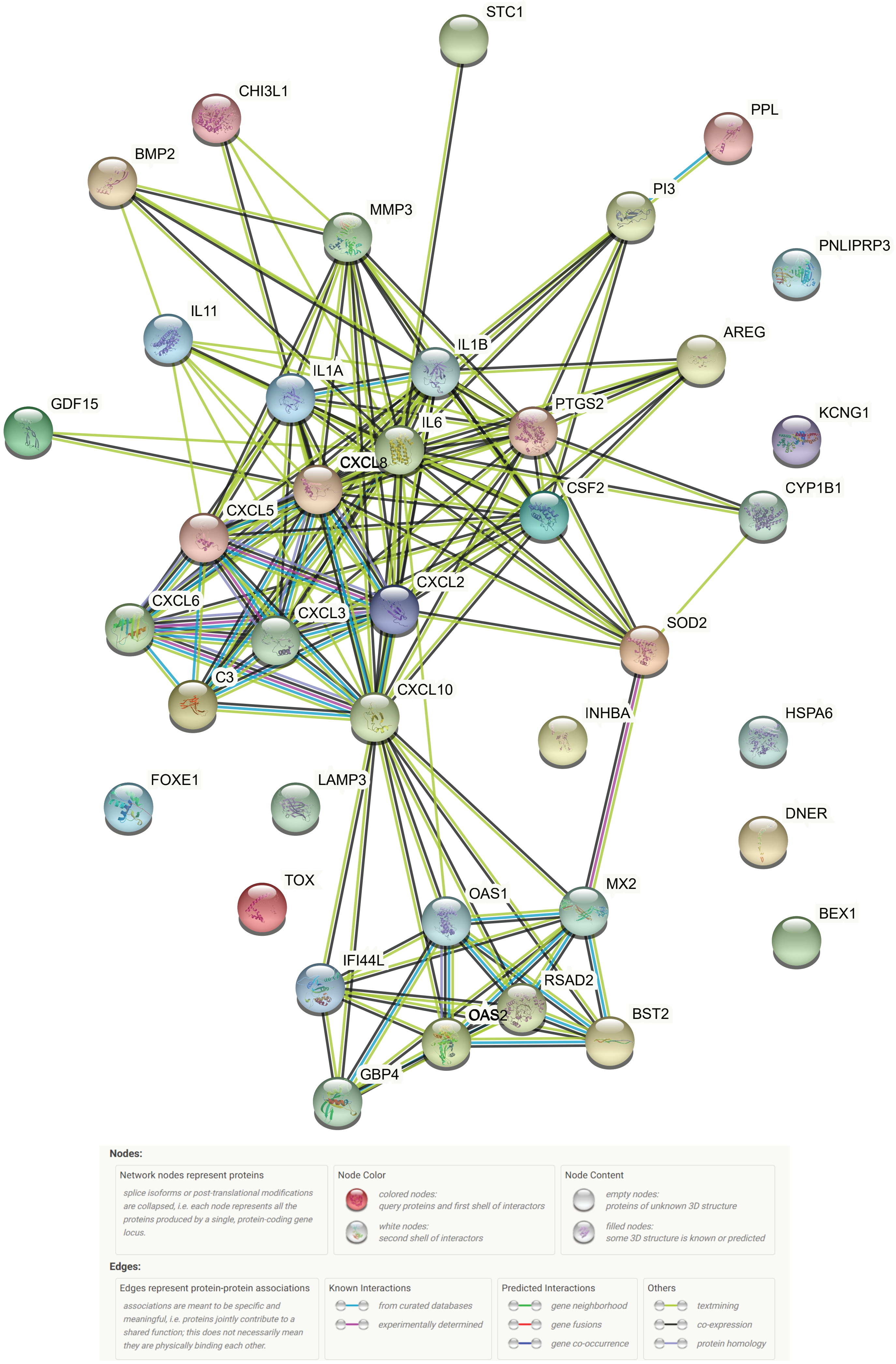

Supplementary Figure 8

F (i)

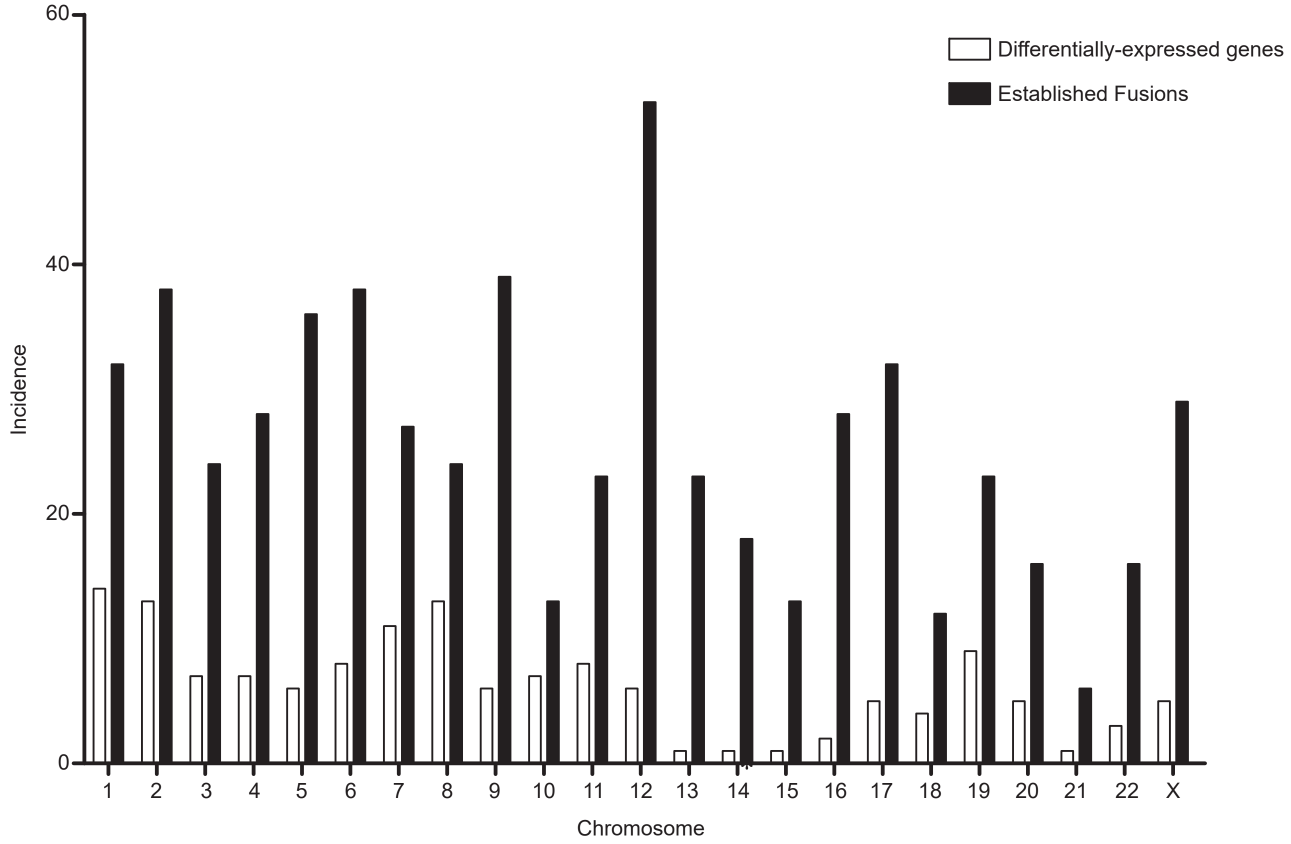

(ii)

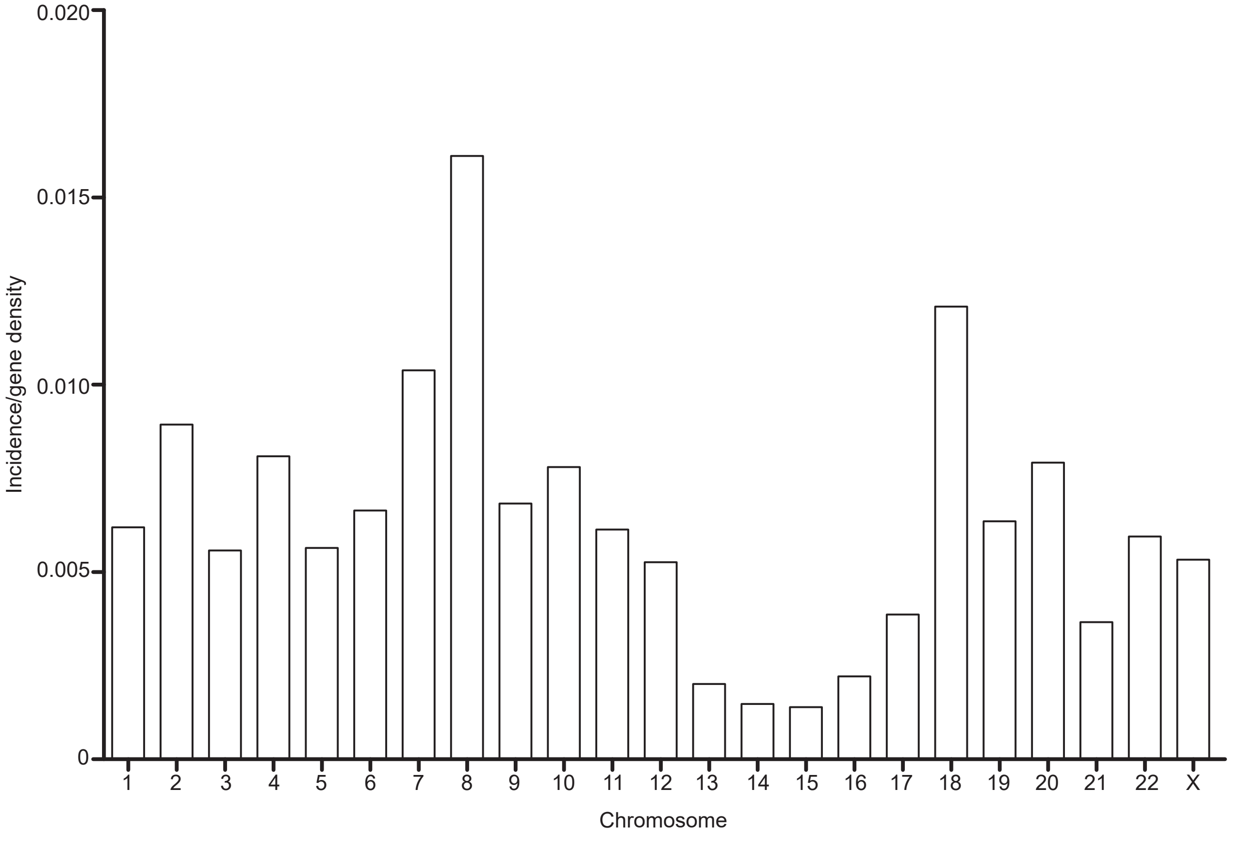

Supplementary Figure 8

G

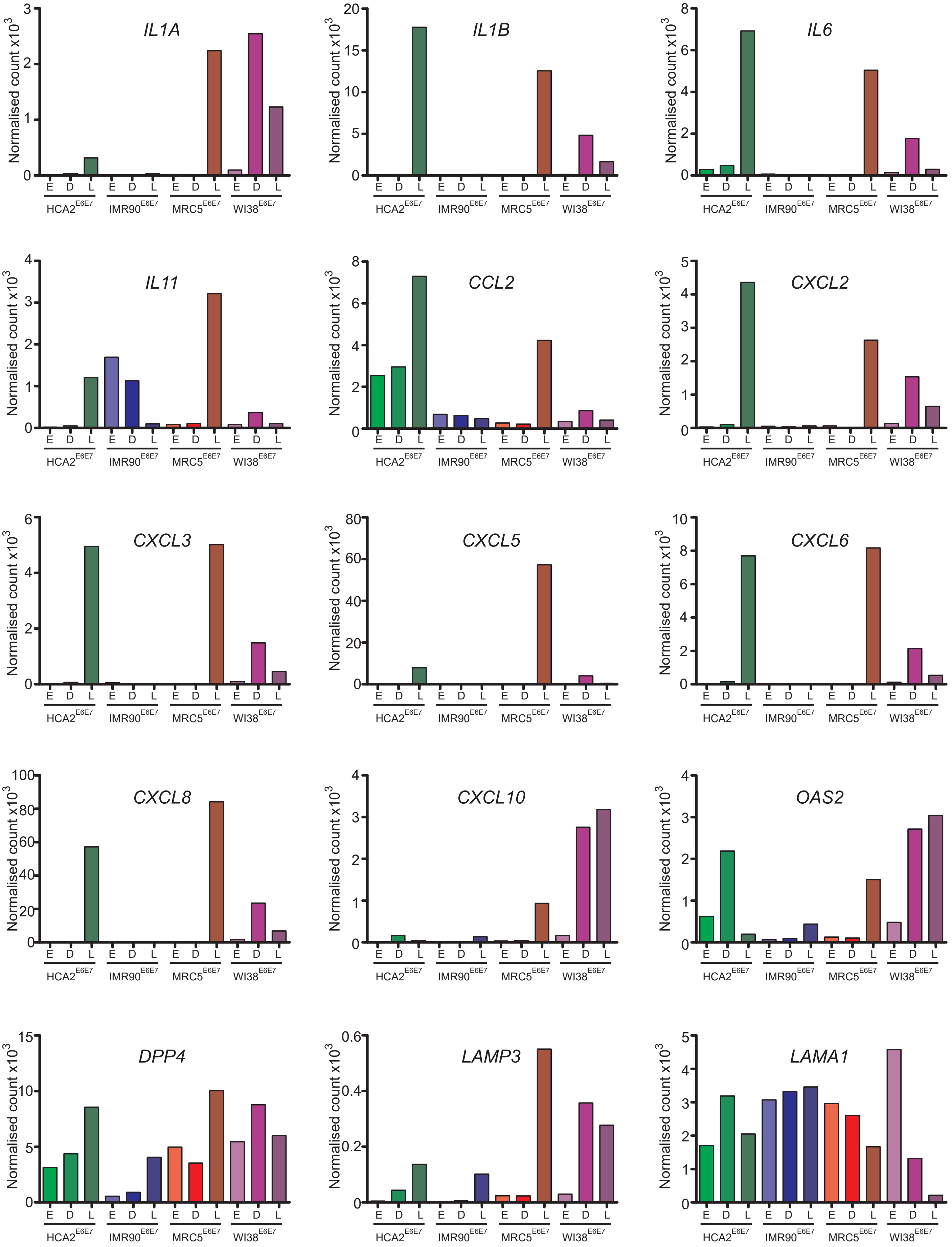

Supplementary Figure 8

H (i)

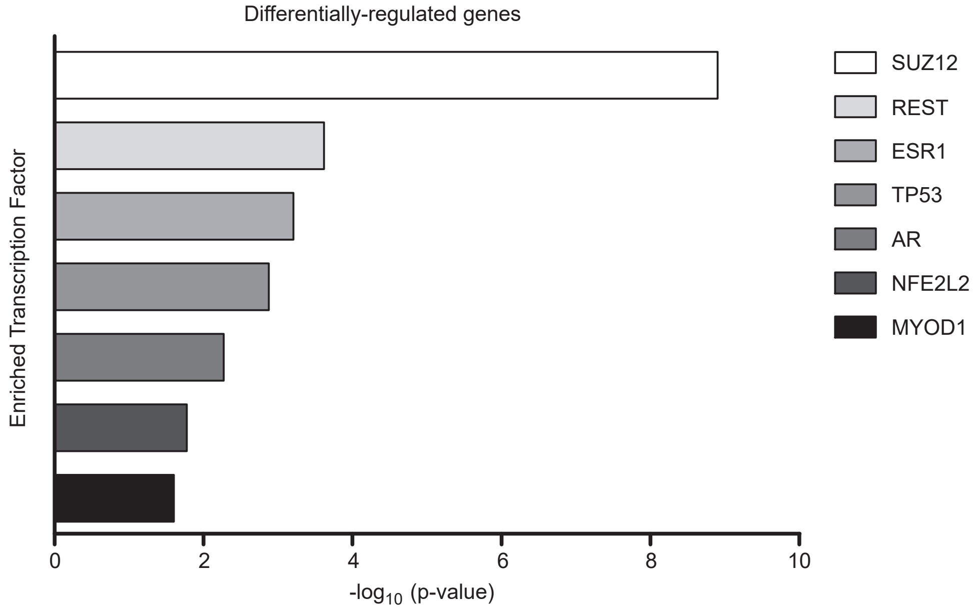

(ii)

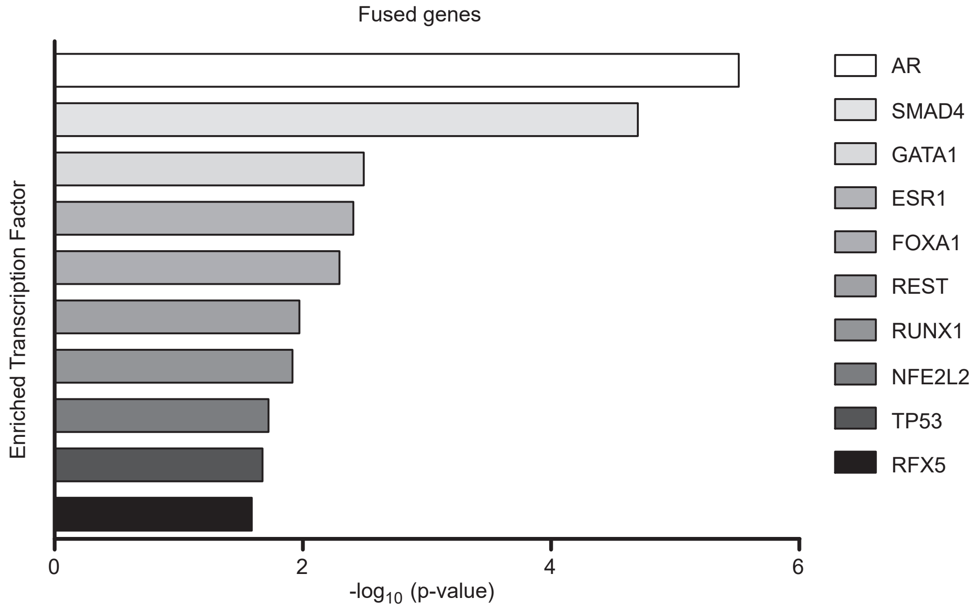

Supplementary Figure 8

I

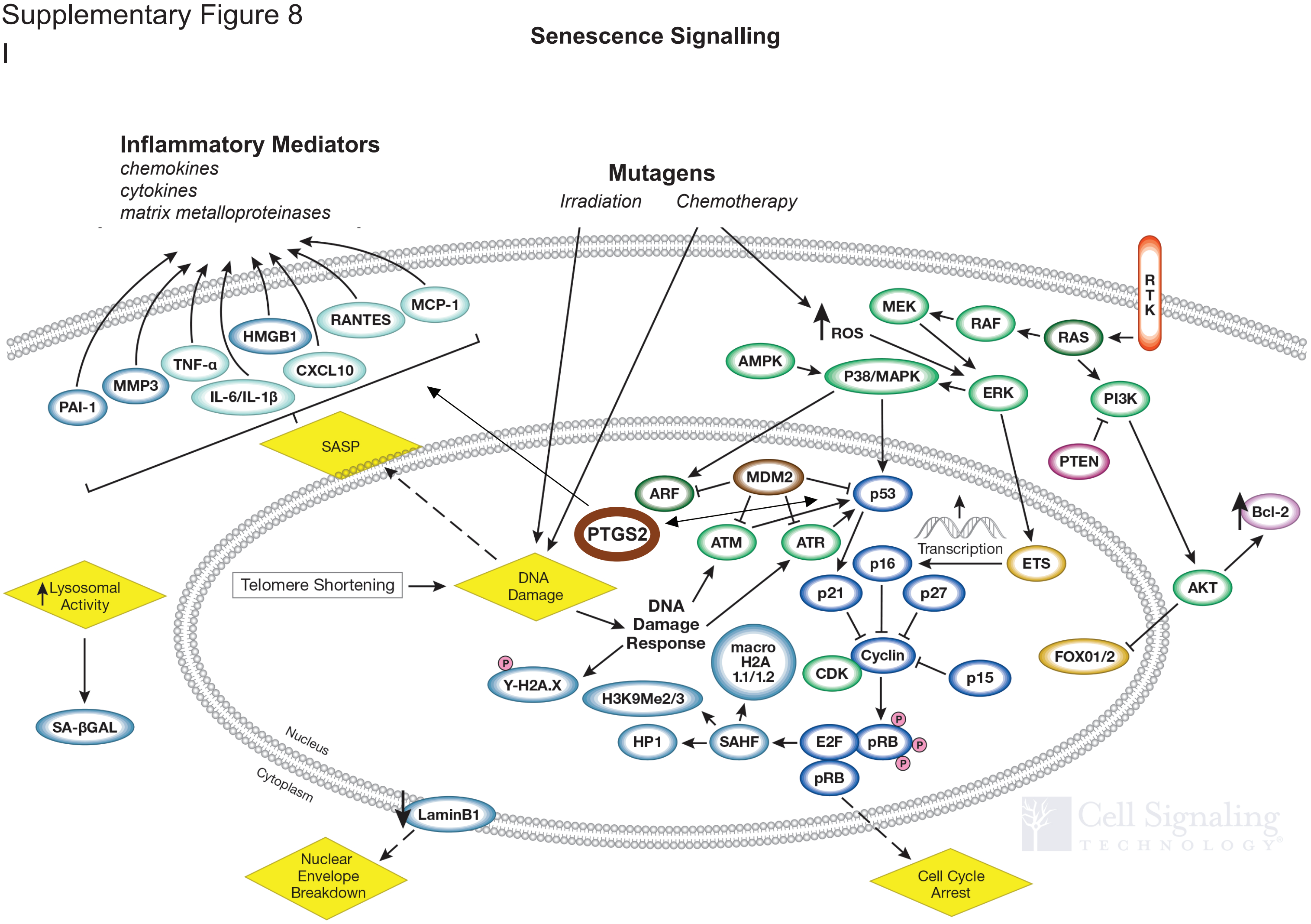

Illustration adapted courtesy of Cell Signaling Technology, Inc. 2020

Supplementary Figure 9

A

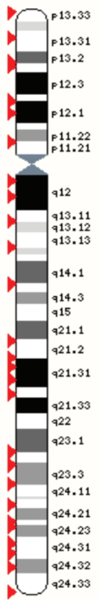

B

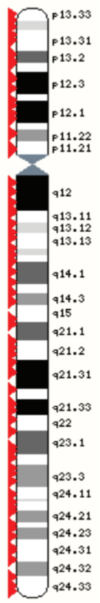

Supplementary Figure 9

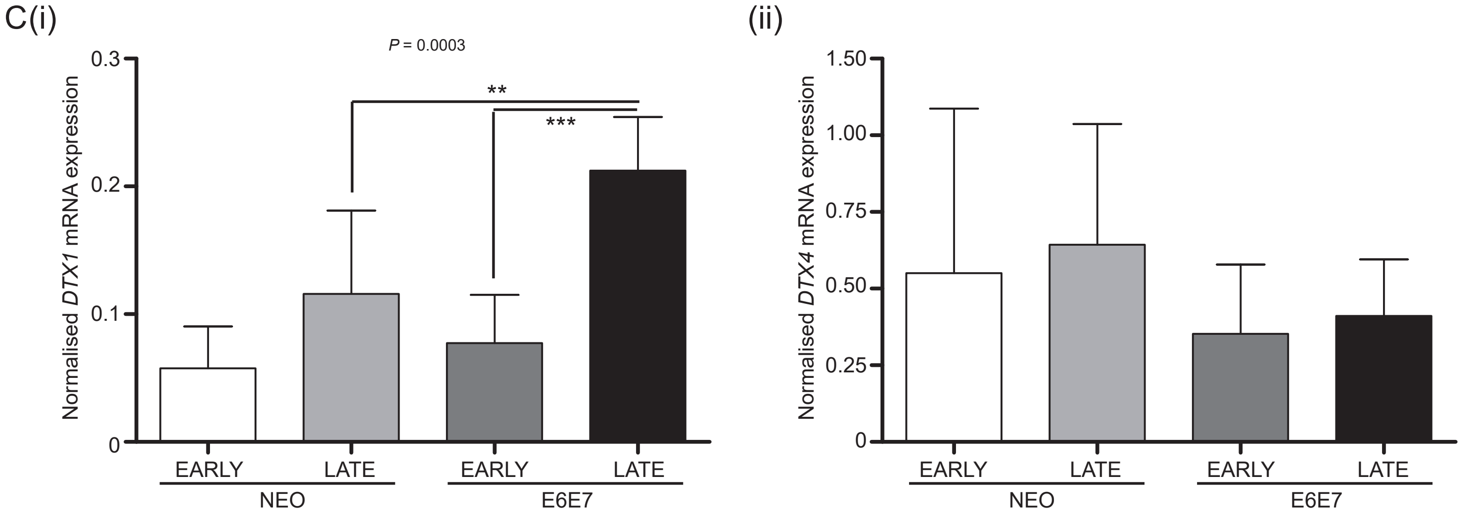

Supplementary Figure 9

D(i)

12:127239859 12:127240137

DENR TGTACATATAGGTTTAACATTTCAA**CAG**TGAAGTCAGTGAAGGCT....ATTCCTTGATTCTAATGAGG**GTCCCC**GGCGGCGCAGAGACGAGTGGAA

12:122762137 12:122762161 21:46698621 21:46698594

12:124343916 12:124343952

NCOR2 CCATGCCTGGCCGGAAGACAATTTTAAAATGT**CACAG**ATCTGAACAGACACTTCACCAAAGAAGATCT

21:46696604 21:46696532

12:132833948 12:132833991

CHFR TGCGTGACAAGGAGGACCAATGCACGCACAGATCTGGGAGAAG**A**AACAGCCTAAGAGTCCTGTGCAGGGATTTTTATTTTGGACTTCTTCACA

21:46697959 21:46697910

(ii)

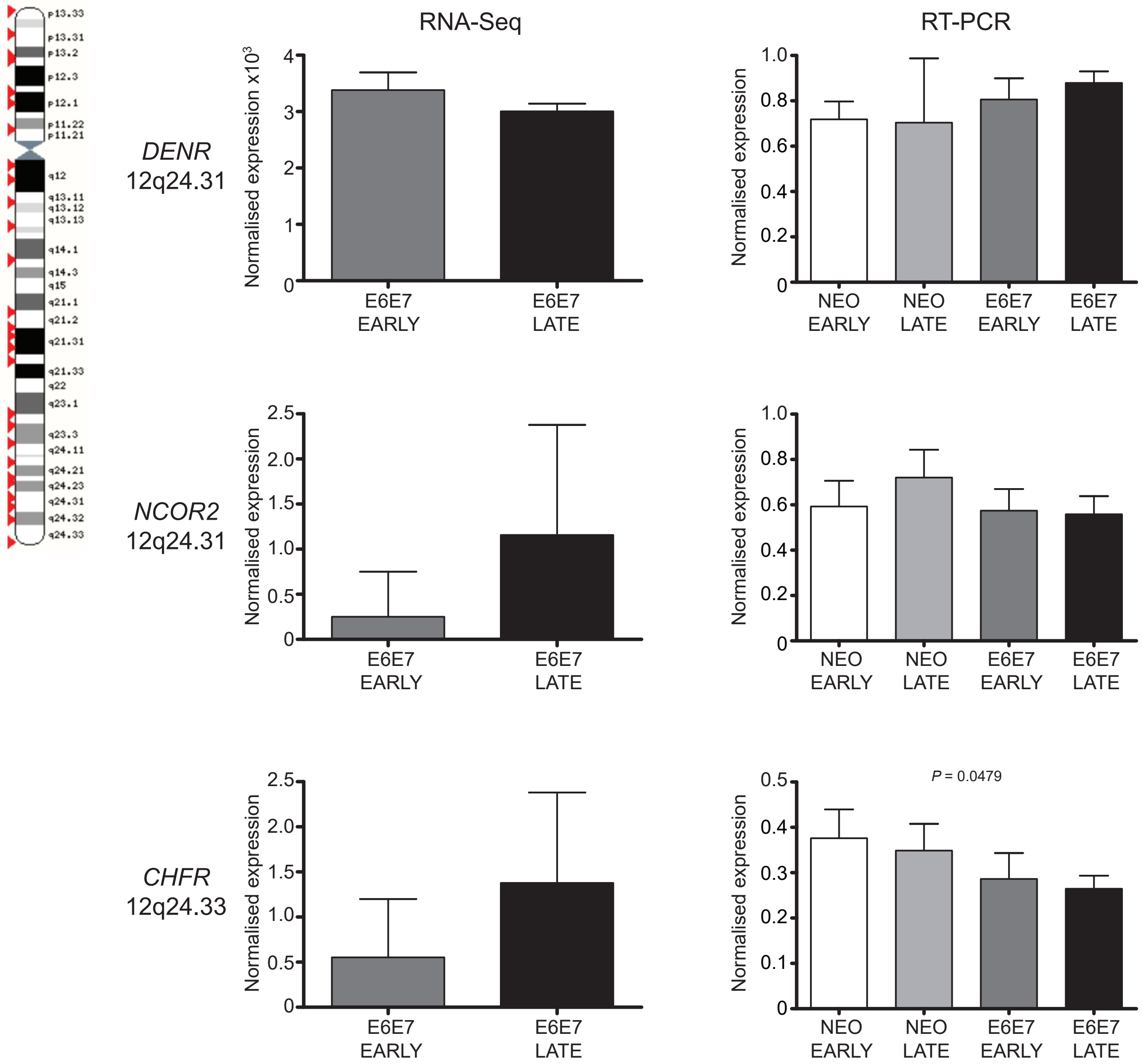

Supplement: zcaa044_Supplemental_Files [file zcaa044_supplemental_files.zip › Liddiard et al Supplementary Figures 021220.pdf]
