# Supplementary material for: Tracking telomere fusions through crisis reveals conflict between DNA transcription and the DNA damage response
Source: NAR Cancer. 2021 Jan 6;3(1):zcaa044. doi: 10.1093/narcan/zcaa044 (PMC7787266; doi:10.1093/narcan/zcaa044)
Supplement: zcaa044_Supplemental_Files [file zcaa044_supplemental_files.zip › Liddiard et al Supplementary Methods 151220.docx]

**Supplementary Materials and Methods**

**RT-PCR analyses of gene expression**

For RT-PCR validation experiments, RNA was extracted from PBS-washed fibroblast and HCT116 cell pellets using the Machery-Nagel Nucleospin RNA Plus kit, followed by removal of genomic DNA using the Ambion DNA-*free*™ DNA Removal Kit. RNA was quantified and purity-checked using the NanoDrop One^C^ Spectrophotometer (ThermoFisher) and 1 μg each reverse transcribed for RT-PCR using the Applied Biosystems™ High-Capacity cDNA Reverse Transcription Kit.

Typically, 1 μL each cDNA was used in 10 μL RT-PCR reactions using the following primer pairs:

| Gene target | Forward primer 5′-3′ sequence | Reverse primer 5′-3′ sequence | Amplicon sizes |
| --- | --- | --- | --- |
| *YWHAZ* | TTCTTGATCCCCAATGCTTC | AGTTAAGGGCCAGACCCAGT | cDNA 211bp  gDNA 828bp |
| *WIF1* | GGTGGCAGCATTTGAAGTGG | CACAGTGAGGTCCGTGGAAC | cDNA 191bp  gDNA 1164bp |
| *DTX1* | CCACATGTACCACCTGCTGT | GGGAATCCTCTTGCGGTGAA | cDNA 264bp  gDNA 479bp |
| *DTX4* | TGCACCATCTGTATGGAACG | GGGGGATGCTGTAGATGAT | cDNA 469bp  gDNA 3258bp |
| *IRAK3* | GTCCCACCTAGAACATCAGAGT | GAGAAATTCCGAGGGCAGGG | cDNA 291bp  gDNA 641bp |
| *HELB* | GCAAACACTACACAGGCCAC | AACTGTTTGCTCCTCGGACC | cDNA 422bp  gDNA 8419bp |
| *DYNLL1* | GGTTTCGGTAGCGACGGTAT | CACCGAGTCCTGTTGCATCT | cDNA 165bp  gDNA 364bp |
| *DENR* | GTGCCTCAGTAACAGGGGAG | CCTCTCTCCCTTTGGCCATA | cDNA 198bp  gDNA 310bp |
| *NCOR2* | CCTCTACTCCTTCCCTGGGG | ACGTCTTGTTTGGCTCTGGA | cDNA 152bp  gDNA 1296bp |
| *CHFR* | GCCTTTCTGCCACCTGTACT | GAGGCTCTCGGTCAACATGT | cDNA 193bp  gDNA 1632bp |

Amplicons were resolved by agarose (1% weight/volume) gel electrophoresis with ethidium bromide DNA staining and images were captured using a Vilbert Lourmat Ebox VX2 gel documenter. 1 kb and 100 bp DNA ladders were routinely run for sizing of amplicons. Quantification of amplicon signal intensity with local background correction was performed using ThermoFisher MyImageAnalysis software and normalised to values calculated for the constitutively-expressed Tyrosine 3-Monooxygenase/Tryptophan 5-Monooxygenase Activation Protein Zeta (*YWHAZ*) control gene within the same samples. Normalised gene expression values are presented as tester/*YWHAZ* signal intensity.

**Protein extraction and Western blotting**

Whole cell lysates were typically prepared from 3x10^5^ PBS-washed and snap-frozen fibroblast cells using 100 μL RIPA buffer supplemented with fresh protease inhibitors. Phosphatase inhibitors were included where it was required to assess stability of phosphorylated proteins. Proteins were extracted by centrifugation and single use 20 μL aliquots were stored at -80°C. Lysates were quantified using the Pierce Coomassie Protein Assay Kit with a BSA protein standard curve read at 595 nm on a BioTek Cytation 3 plate reader. Lysate equivalent cell volumes (approximately 20 μg) were boiled in 6X Laemmli's loading dye with β-mercaptoethanol and run on a 4-12% Tris-glycine gradient gel for 80 min at 100V. Transfer to PVDF membranes was at 100V for 60 min with ice packs. Membranes were blocked in 5% milk-TBST for 1 hour at room temperature before incubation with primary antibodies diluted in 5% milk-TBST at 4◦C overnight with agitation.

***Primary antibodies used in this study were:***

GeneTex anti-DTX1 [C1C3] central domain rabbit IgG polyclonal (GTX112367)

GeneTex anti-DTX4 N-terminal rabbit IgG polyclonal (GTX80971)

Sigma anti-C-terminal actin fragment rabbit IgG polyclonal (A2066)

Santa-Cruz anti-vinculin [H10] 1-300aa mouse monoclonal IgG_2a_ (sc-25336)

Amersham ECL secondary HRP-conjugated anti-rabbit (NA934) or anti-mouse (NA931) antibodies were used, as appropriate, at 1/10000 dilution in 5% milk-TBST with incubation for 1 hr at room temperature. Pierce™ ECL Plus Western Blotting Substrate was used for signal detection via exposure to X-ray film. Membranes were stripped using a mild stripping glycine-SDS protocol at room temperature before repeat blocking and immunoprobing. Quantification of signal volume (intensity) from scanned images was performed using ThermoFisher MyImageAnalysis software. Sample protein equivalence was assessed by pre-lysis cell counts, lysate protein quantification and comparison with actin and vinculin housekeeping signal determined by immunoprobing.

**Subtelomere TAL effector nucleases (TALENs)**

The chr17p and chr21q TALEN pairs are described in^1, 2^ and were custom-synthesised by LabOmics S.A. In summary, the chr17p TALEN pair was designed to target the unique human chr17p subtelomere sequence at 17:113612, 32 bp centromeric of start of the telomere repeat array. The chr21q TALEN pair was designed to target sequence 1.5 kb from the start of the telomere repeat arrays of the homologous chr16p- and chr21q-related telomere families, at 21:46698352.

**TALEN transfections**

Subconfluent (50 to 80%) HCT116 cells were nucleofected with 2.5 μg of each left and right TALEN plasmid in batches of 1x10^6^ cells/cuvette in 100 μL supplemented nucleofection mix (Lonza SE Cell Line Kit L) using the Amaxa 4D nucleofector program DS-138.

Subconfluent (60 to 80%) NEO and E6E7 HCA2 and IMR90 fibroblasts were nucleofected with 1.25 μg of each left and right TALEN plasmid in batches of 1.5x10^5^ cells/cuvette in 100 μL supplemented nucleofection mix (Lonza SE Cell Line Kit L) using the Amaxa 4D nucleofector programs CA-137 (HCA2) and CM-120 (IMR90).

All cells were recovered for 10 min post-nucleofection in unsupplemented RPMI at 25°C before transfection replicates were pooled and plated in media appropriate to the cell type. Control (no DNA) or GFP plasmid transfections were performed in each experiment to determine transfection efficiency as well as the background level of DNA damage and spontaneous telomere fusion (particularly in E6E7 samples). Carrier control treatments were likewise included for normalisation of all experimentally-induced effects.

**Determination of transfection efficiency**

Transfection efficiency was estimated from plasmid-driven GFP (Lonza pmaxGFP) expression in parallel transfections performed in each experiment. Single cell suspensions were supplemented with Hoechst 33342 at a final concentration of 10 μg/mL. Samples were incubated for 15 min at 37°C before propidium iodide was added to a final concentration of 10 μg/mL. GFP expression in viable cell populations was measured using the NC-3000 image cytometer and extrapolated to the sample bulk as a transfection efficiency percentage.

**RNA-Seq bioinformatics**

Preliminary mapping, count generation and exploratory analyses of RNA-Seq data obtained from 4 fibroblast (HCA2, IMR90, MRC5 and WI38) cultures undergoing a telomere-driven crisis sampled at 3 different time points was performed by Dr Anna Evans (Wales Gene Park).

***Mapping***

In short, RNA-Seq reads were trimmed of adaptor^3^ and low-quality reads using Trim Galore version 0.4.141 (Babraham Institute), using default parameters in paired-end mode. Trimmed paired-end reads were aligned to the human GRCh38/hg38 reference genome using STAR (v2.5.1b^4^), following the 2-pass method^5^ with the addition of MultimapNMax=1 flag, whereby reads that map to more than one genomic location are treated as being unmapped so as to exclude them from downstream analysis. QC was performed using FastQC version 0.11.2 (Babraham Institute).

Bespoke scripts were used to calculate Reads Per Kilobase Per Million (RPKM) scores for both exons and transcripts defined by the UCSC Genome Browser sourced GRCh38/hg38 human reference RefSeq (NCBI) gene model. Counting was restricted to reads achieving a minimum of 20 mapped reads per exon in the same orientation as the exon to be counted.

***Gene Expression Analyses***

Raw counts were calculated for all samples for both (i) exons and (ii) genes using Subread featureCounts version 1.5.1^6^. Counts were generated for paired-end read fragments summarized at exon level and then aggregated at gene level. Read fragments overlapping more than one feature were excluded from count summaries in order to provide stringent, but robust count data. This featureCounts was run with the following parameters:

- Exon-level: featureCounts –p –B –t “exon” –g “exon” –a RefGene
- Gene-level: featureCounts –p –B –t “exon” –g “gene_id”

featureCounts was used to unambiguously assign paired-end fragments to exons within the hg38 RefSeq model, before summarizing counts at gene level, i.e. each gene is considered as the union of all its exons.

Exploratory differential expression analysis between time points (within fibroblast line series) was performed using DESeq2 (Love 2014), applying regularized log transformation to raw counts, prior to calculation of regularized log2 fold change for pairs of samples (Early vs. Deep, Deep vs. Late, Early vs. Late).

*Where:*

avgLogExpr = (Sample_1_rlog_counts + Sample_2_rlog_counts)/2

rLogFC = Sample_1_rlog_counts – Sample_2_rlog_counts

Features (genes, isoforms, exons) from each pairwise analysis were ranked by sorting regularized log2 fold change values and heatmaps of the rLogFC top 50 differentially-expressed genes were produced to visualize clustering and key expression changes between samples from the same lineage.

***Gene length***

For gene length evaluations, Biomart Ensembl gene annotation was used and gene length was calculated as chromosome (start – end) +1, where all negative values were converted to positive.

**Fusion amplicon-Seq and whole genome amplification (WGA)-Seq bioinformatics**

Dr Kez Cleal (Cardiff University) undertook preliminary mapping and analyses pertaining to the 12 telomere fusion amplicon samples amplified from the 4 (HCA2, IMR90, MRC5 and WI38) fibroblast cultures undergoing a telomere-driven crisis sampled at 3 different time points, as well as the 92 single nuclei and 4 bulk 500 nuclei samples generated for all 4 fibroblast lines at the Deep crisis time point. Analogous mapping of fusion amplicon sequence data was also conducted for the DNA ligase-deficient fibroblast (GM16088, GM16096 and GM17523) cells undergoing a telomere-driven crisis sampled at 3 time points comparable with the normal fibroblast dataset. All programming and analysis was performed using Python3 and the SciPy ecosystem^7^.

***Mapping paired-end reads***

For telomere fusion data, reads were adapter trimmed using cutadapt (v1.13), while trimgalore (v0.4.3) was used for single cell sequencing data^3^. Reads were mapped to the human GRCh38/hg38 reference genome using bwa mem (v0.7.8) with “-M” flag to mark secondary mappings^8^. Picard tools (v2.7.1; Broad Institute) was used to mark duplicates, whilst sorting and indexing were achieved using sambamba (v0.6.6)^9^. Read-pairs of interest were then isolated (including discordant read-pairs, reads with a supplementary mapping, and reads with a soft-clip > 21 bp in length, but excluding duplicate reads) and converted to FASTQ format. For the fusion amplicon-Seq, a further round of adapter trimming was performed using the original subtelomere amplification primers as input, supplied with the –b option in cutadapt.

***Fusion amplicon-Seq: mapping telomere fusion events***

To obviate mismapping of short-read telomere fusion data to homologous sequences around the genome that are not flanked by the fusion PCR primers used in this study, a novel bioinformatics tool called dodi (available online at https://github.com/kcleal/dodi) was developed. The overall goal of dodi is to generate candidate alignments that are consistent with the experimental design, thus placing telomere fusion amplicon alignments at the expected loci, rather than at a competing homologous region elsewhere in the genome. Candidate alignments to the reference were produced with bwa mem, using the ‘-a’ flag before analysis using dodi. To selectively map reads to expected genomic loci without utilizing a custom reference genome, dodi implements a read-pairing algorithm similar to bwa mem^8^, but read alignments that overlap target genomic loci temporarily receive a small bias in their alignment scores, increasing the alignment score by 15 % by default. Thus, during read-pairing, such reads are chosen over competing alignments with near-identical alignment scores. In the output sam file, dodi retains the original alignment and mapping quality scores calculated by bwa mem. Experimental subtelomere loci in the GRCh38 reference were mapped to the reference genome using Last (http://last.cbrc.jp) with parameters -q4, and selecting the highest scoring alignment^10^. The locations identified for the subtelomere sequences were as follows, chr17p = chr17: 113573-116638, chrXpYp = chrX: 10028-11687, chr21q = chr21:46696195-46699882. The final sets of alignments selected using dodi were marked for duplicates using samblaster before sorting and indexing using samtools^11, 12^.

## *Telomere fusion event-calling*

To identify telomere fusions, dysgu-SV was utilized (found online at https://github.com/kcleal/dysgu), using parameters –min-mapq 0, --min-support 1. Dysgu-SV analyses discordant reads, split reads, read depth and contig assembly to identify SVs (Structural Variants). SV events were categorized as ‘genomic’ if one of the two fusion junction components occurred outside the subtelomere regions of interest, ‘intra-chromosomal’ if both junction points were within the same subtelomere, or ‘inter-chromosomal’ if junctions derived from different subtelomere loci. Machine learning models were trained using scikit-learn (https://scikit-learn.org/stable/) for further refinement of the datasets, with separate models generated for the ‘genomic’ versus the ‘intra/inter-chromosomal’ events. For ‘genomic’ events an ExtraTreesClassifier model was used with n_estimaters=200 and max_depth=40. Training data consisted of 20,000 manually-curated fusion events that were initially called using dysgu-SV and model features corresponded to the SV annotations provided in the output of dysgu-SV. Using 10 times K-fold cross validation, the genomic model achieved a precision of 0.77 and recall 0.73. For ‘intra/inter-chromosomal’ events, an ExtraTreesClassifier model was used with n_estimators=100, and training data consisted of 246 manually-labelled fusion events called with dysgu-SV that were randomly sampled from the same dataset. The ‘intra/inter-chromosomal’ model achieved a cross-validated precision of 0.91 and recall 0.96.

***Telomere fusion junction annotation***

Using a custom script, calls from dysgu-SV were annotated for telomere fusion junction microhomology, insertions and templated insertions. Microhomology and insertions were identified by first aligning the two contigs generated by dysgu-SV, referred to as ‘contigA’ and ‘contigB’ in the dysgu-SV output file. These correspond to the consensus sequences generated from each SV junction. Alignment was performed using scikit-bio using striped Smith-Waterman with parameters (match_score=1, mismatch_score=-3). The reverse-complement alignment was also assessed in case of inversion rearrangements, keeping the best alignment for further filtering. Filtering was performed to ensure only high-quality alignments were used for annotation. Alignments were discarded if insertion or deletions were identified in the alignment or an alignment score of < 41 was returned. The alignment was also required to be composed of reference-aligned bases and soft-clipped bases for both contigA and contigB. Microhomology was identified by the overlap of reference-aligned bases in the contigA-contigB alignment, whereas insertions were identified as stretches of soft-clipped bases from both contigA and contigB that align to one another.

To identify templated insertions, any identified insertions were aligned to the reference sequence upstream and downstream of the fusion junction. Specifically, insertions were aligned to three different sized windows centred over each junction, with flanking window sizes ranging from 50 (+/- 25 bp), 100 and 200 bp. If neighbouring windows overlapped due to the proximity of junctions, overlapping windows were divided at the midpoint of the intersection to ensure the same sequence was not analysed twice. The highest scoring alignment from each window was then assessed further using a Monte Carlo simulation to determine the probability of obtaining an alignment with score equal to or greater than the observed score. Random sequences of length equal to the search windows of 50, 100 or 200 bp were generated, then for each candidate insertion, 1000 random sequences of length equivalent to the insertion, were generated and aligned to the randomized search windows, generating a probability distribution of alignment scores for random sequences. The probability of an observed alignment score was then determined by using the cumulative density function: $P(X \geq a)=1-CDF (s)$, where s is the proportion of simulated alignments with score less than the observed value. The alignment with the lowest probability value was chosen as the unique templated insertion alignment in the output.

***Simulated telomere fusion dataset***

To test the validity of key observations from the experimentally-established telomere fusion datasets, collections of simulated genomic and intra-chromosomal fusions were prepared.

Genomic intervals of 1 kb in length were randomly sampled from the GRCh38 reference genome, keeping intervals from the ‘normal’ chromosome set [chrM, chr1 … chrY], and excluding intervals that overlapped gaps in the reference. The reference sequence from each genomic interval was then joined with another sequence derived from one of the subtelomere regions to create a small genomic fusion sequence. For the subtelomere sequence, the minimum length was 100 bp if the interval extended beyond the primer site and the maximum length was 1 kb otherwise. The genomic sequence and subtelomere sequence were joined with the genomic sequence extending from the telomere array side.

Paired-end reads were then randomly sampled from the fusion sequences using NEAT^13^, with parameters (-p 1 –pe 210 175 –R 125). Read coverage for each fusion was randomly chosen from a minimum of 2 to a maximum of 15 X. For each simulated sample, 100 fusions per subtelomere region were generated, and 40 samples were generated in total. Each simulated sample was then processed using the same procedure as the real data and called fusion events were categorised as True or False positives based on the *P*-value threshold of 0.5. In total, there were 9484 true positive genomic fusion events and 3810 true positive intra-chromosomal fusion events. Simulated read pairs were intersected with Ensembl and Repeatfinder annotation tracks to curate gene and repeat motif coincidence with simulated fusion gene junctions.

***WGA-Seq single nuclei Copy Number analysis***

Genome Copy Number (CN) profiles were determined according to^14^. Briefly, sequencing depth was analysed using non-overlapping bins of 50 kb in length. Genomic bins were discarded if they had a mean coverage value of > 50. To identify relative CN changes, bins were normalized against the genomic median before performing a background subtraction using the 500 nuclei bulk sample as background. CN profiles were then smoothed using a Haar wavelet transform using Scipy and PyWavelets^7^ and profiles were segmented using the R package copyNumber with parameters gamma=100 And kmin=3^15^. Heatmaps were generated using the copyNumber package.

***Intersections of fusions and single nuclei Copy Number Alterations (CNA)***

Intersections between telomere fusion junctions and CNA were performed using BedTools, with two-tailed *P*-values determined using the Fisher’s exact function from BedTools^16^. CN segments with mean relative CN changes between -0.5 and 0.5 were not analysed for intersections. Heatmaps were generated with matplotlib^17^.

**References**

1. Liddiard K, Ruis B, Takasugi T, et al. Sister chromatid telomere fusions, but not NHEJ-mediated inter-chromosomal telomere fusions, occur independently of DNA ligases 3 and 4. *Genome Res*. 05 2016;26(5):588-600. doi:10.1101/gr.200840.115

2. Liddiard K, Ruis B, Kan Y, et al. DNA Ligase 1 is an essential mediator of sister chromatid telomere fusions in G2 cell cycle phase. *Nucleic Acids Research*. 2018:gky1279-gky1279.

3. Martin M. Cutadapt removes adapter sequences from high-throughput sequencing reads. next generation sequencing; small RNA; microRNA; adapter removal. *2011*. 2011-05-02 2011;17(1):3. doi:10.14806/ej.17.1.200

4. Dobin A, Davis CA, Schlesinger F, et al. STAR: ultrafast universal RNA-seq aligner. *Bioinformatics*. Jan 1 2013;29(1):15-21. doi:10.1093/bioinformatics/bts635

5. Dobin A, Gingeras TR. Mapping RNA-seq Reads with STAR. *Curr Protoc Bioinformatics*. Sep 2015;51:11.14.1-11.14.19. doi:10.1002/0471250953.bi1114s51

6. Liao Y, Smyth GK, Shi W. featureCounts: an efficient general purpose program for assigning sequence reads to genomic features. *Bioinformatics*. 2013;30(7):923-930. doi:10.1093/bioinformatics/btt656

7. Virtanen P, Gommers R, Oliphant TE, et al. SciPy 1.0: fundamental algorithms for scientific computing in Python.

8. Li H, Durbin R. Fast and accurate short read alignment with Burrows-Wheeler transform. *Bioinformatics*. Jul 15 2009;25(14):1754-60. doi:10.1093/bioinformatics/btp324

9. Tarasov A, Vilella AJ, Cuppen E, Nijman IJ, Prins P. Sambamba: fast processing of NGS alignment formats. *Bioinformatics*. Jun 15 2015;31(12):2032-4. doi:10.1093/bioinformatics/btv098

10. Kiełbasa SM, Wan R, Sato K, Horton P, Frith MC. Adaptive seeds tame genomic sequence comparison. *Genome Res*. Mar 2011;21(3):487-93. doi:10.1101/gr.113985.110

11. Li H, Handsaker B, Wysoker A, et al. The Sequence Alignment/Map format and SAMtools. *Bioinformatics*. Aug 2009;25(16):2078-9. doi:10.1093/bioinformatics/btp352

12. Faust GG, Hall IM. SAMBLASTER: fast duplicate marking and structural variant read extraction. *Bioinformatics*. Sep 2014;30(17):2503-5. doi:10.1093/bioinformatics/btu314

13. Stephens ZD, Hudson ME, Mainzer LS, Taschuk M, Weber MR, Iyer RK. Simulating Next-Generation Sequencing Datasets from Empirical Mutation and Sequencing Models. *Plos One*. 2016;11(11):e0167047. doi:10.1371/journal.pone.0167047

14. Cleal K, Jones RE, Grimstead JW, Hendrickson EA, Baird DM. Chromothripsis during telomere crisis is independent of NHEJ, and consistent with a replicative origin. *Genome Res*. 05 2019;29(5):737-749. doi:10.1101/gr.240705.118

15. Nilsen G, Liestøl K, Van Loo P, et al. Copynumber: Efficient algorithms for single- and multi-track copy number segmentation.

16. Quinlan AR, Hall IM. BEDTools: a flexible suite of utilities for comparing genomic features. *Bioinformatics*. Mar 2010;26(6):841-2. doi:10.1093/bioinformatics/btq033

17. Hunter JD. Matplotlib: A 2D Graphics Environment. *Computing in Science & Engineering*. 2007;9(3):90-95. doi:10.1109/MCSE.2007.55
