# Supplementary material for: Tracking telomere fusions through crisis reveals conflict between DNA transcription and the DNA damage response
Source: NAR Cancer. 2021 Jan 6;3(1):zcaa044. doi: 10.1093/narcan/zcaa044 (PMC7787266; doi:10.1093/narcan/zcaa044)
Supplement: zcaa044_Supplemental_Files [file zcaa044_supplemental_files.zip › Liddiard et al Supplementary Tables 1-3 021220.pdf]

## Supplementary Table 1

| Sample | Time point | Mean 17p TL (kb) | 95% CI | Mean short allele 17p TL (kb) | 95% CI | Shortest 17p TL (kb) | Longest 17p TL (kb) |
|---|---|---|---|---|---|---|---|
| HCA2$^{E6E7}$ | EARLY | 3.413 | 2.778-4.048 | 2.757 | 2.430-3.085 | 0.982 | 12.189 |
| | DEEP | 3.203 | 2.754-3.652 | 1.972 | 1.846-2.097 | 0.750 | 11.806 |
| | LATE | 3.493 | 3.069-3.917 | 1.677 | 1.584-1.769 | 0.744 | 14.177 |
| IMR90$^{E6E7}$ | EARLY | 3.053 | 2.777-3.329 | 1.530 | 1.395-1.666 | 0.784 | 6.550 |
| | DEEP | 2.473 | 2.239-2.707 | 1.658 | 1.514-1.801 | 0.714 | 9.066 |
| | LATE | 2.191 | 1.923-2.459 | 1.415 | 1.281-1.548 | 0.637 | 9.416 |
| MRC5$^{E6E7}$ | EARLY | 1.982 | 1.839-2.125 | 1.339 | 1.268-1.411 | 0.647 | 5.136 |
| | DEEP | 1.825 | 1.677-1.972 | 1.29 | 1.217-1.363 | 0.554 | 4.829 |
| | LATE | 2.156 | 1.910-2.403 | 1.209 | 1.109-1.309 | 0.642 | 4.268 |
| WI38$^{E6E7}$ | EARLY | 2.440 | 1.857-3.022 | 1.751 | 1.629-1.873 | 0.705 | 17.553 |
| | DEEP | 1.510 | 1.401-1.620 | 1.075 | 1.013-1.137 | 0.608 | 3.147 |
| | LATE | 1.479 | 1.392-1.566 | 1.063 | 1.005-1.120 | 0.604 | 2.632 |

## Supplementary Table 2

| Sequencing assay | Sample | Time point | Sample code | Illumina HiSeq paired-end read count lane 1 | Illumina HiSeq paired-end read count lane 2 |
|---|---|---|---|---|---|
| Fusion-Seq | $HCA2^{E6E7}$ | EARLY | DB119 | 10811956 | 10593845 |
| | | DEEP | DB120 | 14200585 | 14027681 |
| | | LATE | DB121 | 12713704 | 12479558 |
| | $IMR90^{E6E7}$ | EARLY | DB122 | 12631677 | 12373366 |
| | | DEEP | DB123 | 15675596 | 15469219 |
| | | LATE | DB124 | 14807844 | 14526952 |
| | $MRC5^{E6E7}$ | EARLY | DB125 | 17010664 | 16708790 |
| | | DEEP | DB126 | 12022055 | 11616128 |
| | | LATE | DB127 | 14153043 | 13754052 |
| | $WI38^{E6E7}$ | EARLY | DB128 | 9023780 | 8746618 |
| | | DEEP | DB129 | 12050285 | 11700862 |
| | | LATE | DB130 | 12634172 | 12208997 |
| RNA-Seq | $HCA2^{E6E7}$ | EARLY | RDB119 | 28918364 | |
| | | DEEP | RDB120 | 33209046 | |
| | | LATE | RDB121 | 34732793 | |
| | $IMR90^{E6E7}$ | EARLY | RDB122 | 30110627 | |
| | | DEEP | RDB123 | 28709555 | |
| | | LATE | RDB124 | 27108329 | |
| | $MRC5^{E6E7}$ | EARLY | RDB125 | 35512954 | |
| | | DEEP | RDB126 | 28713329 | |
| | | LATE | RDB127 | 31818187 | |
| | $WI38^{E6E7}$ | EARLY | RDB128 | 24648830 | |
| | | DEEP | RDB129 | 28790747 | |
| | | LATE | RDB130 | 30025336 | |
| WGA-Seq | $HCA2^{E6E7}$ | DEEP | SDB120.25 | 3905420 | |
| | | DEEP | SDB120.26 | 4153549 | |
| | | DEEP | SDB120.27 | 3607762 | |
| | | DEEP | SDB120.28 | 4094199 | |
| | | DEEP | SDB120.29 | 4035118 | |
| | | DEEP | SDB120.30 | 3623421 | |
| | | DEEP | SDB120.31 | 3690697 | |
| | | DEEP | SDB120.32 | 3531779 | |
| | | DEEP | SDB120.33 | 4019560 | |
| | | DEEP | SDB120.34 | 4501637 | |
| | | DEEP | SDB120.35 | 3613827 | |
| | | DEEP | SDB120.36 | 3988032 | |
| | | DEEP | SDB120.37 | 4044297 | |
| | | DEEP | SDB120.38 | 4331464 | |
| | | DEEP | SDB120.39 | 3856988 | |
| | | DEEP | SDB120.40 | 3533832 | |
| | | DEEP | SDB120.41 | 3563627 | |
| | | DEEP | SDB120.42 | 3901137 | |
| | | DEEP | SDB120.43 | 3363878 | |
| | | DEEP | SDB120.44 | 3541474 | |
| | | DEEP | SDB120.45 | 3442517 | |
| | | DEEP | SDB120.46 | 4056516 | |

## Supplementary Table 2

| | | | | | |
|---|---|---|---|---|---|
| | | DEEP | SDB120.48 | 3169607 | |
| | 500 nuclei | DEEP | SDB120.47 | 3507543 | |
| | IMR90$^{E6E7}$ | DEEP | SDB123.49 | 4121869 | |
| | | DEEP | SDB123.50 | 2938439 | |
| | | DEEP | SDB123.51 | 3455725 | |
| | | DEEP | SDB123.52 | 4159564 | |
| | | DEEP | SDB123.53 | 3991192 | |
| | | DEEP | SDB123.54 | 4126754 | |
| | | DEEP | SDB123.55 | 3983036 | |
| | | DEEP | SDB123.56 | 3562367 | |
| | | DEEP | SDB123.57 | 4156299 | |
| | | DEEP | SDB123.58 | 4316807 | |
| | | DEEP | SDB123.59 | 3859058 | |
| | | DEEP | SDB123.60 | 4108793 | |
| | | DEEP | SDB123.61 | 4418967 | |
| | | DEEP | SDB123.62 | 4242268 | |
| | | DEEP | SDB123.63 | 4200496 | |
| | | DEEP | SDB123.64 | 3716151 | |
| | | DEEP | SDB123.65 | 4298192 | |
| | | DEEP | SDB123.66 | 4197848 | |
| | | DEEP | SDB123.67 | 3524326 | |
| | | DEEP | SDB123.68 | 3918393 | |
| | | DEEP | SDB123.69 | 3893951 | |
| | | DEEP | SDB123.70 | 4188989 | |
| | | DEEP | SDB123.72 | 3689153 | |
| | 500 nuclei | DEEP | SDB123.71 | 3554590 | |
| | MRC5$^{E6E7}$ | DEEP | SDB126.73 | 3756652 | |
| | | DEEP | SDB126.74 | 4242237 | |
| | | DEEP | SDB126.75 | 3344935 | |
| | | DEEP | SDB126.76 | 3473251 | |
| | | DEEP | SDB126.77 | 3571947 | |
| | | DEEP | SDB126.78 | 3475690 | |
| | | DEEP | SDB126.79 | 3397397 | |
| | | DEEP | SDB126.80 | 3189294 | |
| | | DEEP | SDB126.81 | 2994182 | |
| | | DEEP | SDB126.82 | 2738181 | |
| | | DEEP | SDB126.83 | 3220880 | |
| | | DEEP | SDB126.84 | 4189142 | |
| | | DEEP | SDB126.85 | 2586068 | |
| | | DEEP | SDB126.86 | 2892365 | |
| | | DEEP | SDB126.87 | 3916818 | |
| | | DEEP | SDB126.88 | 3863920 | |
| | | DEEP | SDB126.89 | 3295036 | |
| | | DEEP | SDB126.90 | 4644982 | |
| | | DEEP | SDB126.91 | 3751963 | |
| | | DEEP | SDB126.92 | 2888086 | |
| | | DEEP | SDB126.93 | 3907961 | |
| | | DEEP | SDB126.94 | 4286209 | |
| | | DEEP | SDB126.96 | 3837241 | |

## Supplementary Table 2

| | | | | | |
|---|---|---|---|---|---|
| | 500 nuclei | DEEP | SDB126.95 | 3914531 | |
| | WI38$^{E6E7}$ | DEEP | SDB129.01 | 3565348 | |
| | | DEEP | SDB129.02 | 3172878 | |
| | | DEEP | SDB129.03 | 2663214 | |
| | | DEEP | SDB129.04 | 3299711 | |
| | | DEEP | SDB129.05 | 3327844 | |
| | | DEEP | SDB129.06 | 3414427 | |
| | | DEEP | SDB129.07 | 3532934 | |
| | | DEEP | SDB129.08 | 2650174 | |
| | | DEEP | SDB129.09 | 2107612 | |
| | | DEEP | SDB129.10 | 2753104 | |
| | | DEEP | SDB129.11 | 2351910 | |
| | | DEEP | SDB129.12 | 2224703 | |
| | | DEEP | SDB129.13 | 2686079 | |
| | | DEEP | SDB129.14 | 2165956 | |
| | | DEEP | SDB129.15 | 2165470 | |
| | | DEEP | SDB129.16 | 1634686 | |
| | | DEEP | SDB129.17 | 3669575 | |
| | | DEEP | SDB129.18 | 4039922 | |
| | | DEEP | SDB129.19 | 3214719 | |
| | | DEEP | SDB129.20 | 3807298 | |
| | | DEEP | SDB129.21 | 3471007 | |
| | | DEEP | SDB129.22 | 3383931 | |
| | | DEEP | SDB129.24 | 3064796 | |
| | 500 nuclei | DEEP | SDB129.23 | 2704697 | |

**Supplementary Table 3**

| Sample | Gene symbol | GRCh38 location | Transcript ID | DESeq2 normalised counts EARLY | DEEP | LATE |
|---|---|---|---|---|---|---|
| HCA2 | COL3A1 | chr2:188974373 | NM_000090 | 95124.25 | 67341.81 | 4996.514 |
| | MMP3 | chr11:102835797 | NM_002422 | 3209.483 | 2780.931 | 44612.15 |
| | CXCL8 | chr4:73740506 | NM_000584 | 47.43078 | 441.5569 | 57175.15 |
| | STC1 | chr8:23841921 | NM_003155 | 493.7665 | 1784.747 | 48693.57 |
| | TMEM158 | chr3:45224464 | NM_015444 | 526.6032 | 2076.194 | 15364.47 |
| | TM4SF1 | chr3:149369018 | NM_014220 | 498.6312 | 1734.061 | 20028.83 |
| | TOX | chr8:58805418 | NM_014729 | 1011.857 | 668.6711 | 33.55054 |
| | BST2 | chr19:17402939 | NM_004335 | 370.933 | 4151.414 | 255.8228 |
| | MXRA5 | chrX:3308566 | NM_015419 | 8977.795 | 4410.695 | 383.3149 |
| | ALDH1A1 | chr9:72900662 | NM_000689 | 6617.201 | 2177.567 | 130.8471 |
| | CXCL12 | chr10:44377062 | NM_199168 | 6052.897 | 3538.303 | 242.4026 |
| | | | NM_000609 | 447.5519 | 309.9671 | 25.1629 |
| | | | NM_001033886 | 88.78068 | 39.96431 | 2.51629 |
| | IL1B | chr2:112829760 | NM_000576 | 21.89113 | 119.8929 | 17754.11 |
| | CXCL5 | chr4:73995642 | NM_002994 | 27.972 | 122.8171 | 7876.827 |
| | CXCL6 | chr4:73836556 | NM_002993 | 40.13373 | 144.2614 | 7684.751 |
| | THBD | chr20:23045633 | NM_000361 | 91.21303 | 554.6266 | 7570.679 |
| | IL6 | chr7:22727142 | NM_000600 | 288.2332 | 482.4959 | 6924.831 |
| | PTGS2 | chr1:186671812 | NM_000963 | 266.3421 | 511.7381 | 13505.77 |
| | INHBA | chr7:41685114 | NM_002192 | 606.8707 | 797.3367 | 9368.149 |
| | SBSPON | chr8:73064543 | NM_153225 | 324.7184 | 14.62109 | 2.51629 |
| | CLEC3B | chr3:45026267 | NM_003278 | 359.9874 | 97.47392 | 1.677527 |
| | LHX9 | chr1:197917387 | NM_001014434 | 1.216174 | 1.949478 | 0 |
| | | | NM_020204 | 205.5334 | 319.7145 | 0 |
| | DAB1 | chr1:56994778 | NM_021080 | 1.216174 | 0 | 0 |
| | | | NM_001353986 | 383.0947 | 248.5585 | 2.51629 |
| | | | NM_001353980 | 13.37791 | 11.69687 | 0.838763 |
| | BMP6 | chr6:7726099 | NM_001718 | 3.648521 | 6.823174 | 590.4895 |
| | HSPA7 | chr1:161606059 | NR_024151 | 4.864695 | 6.823174 | 306.1487 |
| | MMP9 | chr20:46008908 | NM_004994 | 1.216174 | 13.64635 | 352.2806 |
| | EGR4 | chr2:73290929 | NM_001965 | 0 | 0 | 145.9448 |
| | PI3 | chr20:45174899 | NM_002638 | 0 | 0 | 366.5396 |
| | PDK4 | chr7:95583497 | NM_002612 | 0 | 2.924218 | 197.9482 |
| | HLA−DRB1 | chr6_GL000256v2_alt:3851136 | NM_001243965_3 | 2.432348 | 6.823174 | 205.497 |
| | | | NM_001243965_4 | 0 | 0 | 21.80785 |
| | PNLIPRP3 | chr10:116427912 | NM_001011709 | 1.216174 | 7.797914 | 244.0802 |
| | HSPA6 | chr1:161524540 | NM_002155 | 15.81026 | 21.44426 | 2916.38 |
| | CXCL3 | chr4:74036591 | NM_002090 | 8.513216 | 65.30753 | 4954.576 |
| | CXCL2 | chr4:74097037 | NM_002089 | 26.75582 | 104.2971 | 4355.699 |
| | SCG2 | chr2:223596940 | NM_003469 | 25.53965 | 159.8572 | 884.0567 |
| | COL10A1 | chr6:116118922 | NM_000493 | 32.83669 | 75.05492 | 1650.686 |
| | NR4A2 | chr2:156324432 | NM_006186 | 34.05287 | 102.3476 | 1368.862 |
| | NTSR1 | chr20:62708837 | NM_002531 | 13.37791 | 68.23174 | 706.2388 |

| | | | | | | |
|---|---|---|---|---|---|---|
| | SOD2 | chr6:159690922 | NM_001322820 | 24.32348 | 17.54531 | 23.48538 |
| | | | NM_001322819 | 1.216174 | 0.974739 | 0 |
| | | | NM_001322816 | 21.89113 | 64.33279 | 679.3984 |
| | | | NM_001322817 | 0 | 0 | 1.677527 |
| | LINC02154 | chrX:13266048 | NR_146309 | 7.297043 | 113.0697 | 797.664 |
| | FZD8 | chr10:35638249 | NM_031866 | 19.45878 | 101.3729 | 822.8269 |
| | LCP1 | chr13:46125923 | NM_002298 | 9.72939 | 32.16639 | 737.2731 |
| | BMP2 | chr20:6768098 | NM_001200 | 14.59409 | 19.49478 | 956.1903 |
| | CSF2 | chr5:132073792 | NM_000758 | 0 | 51.66118 | 1206.142 |
| | AREG | chr4:74445098 | NM_001657 | 10.94556 | 46.78748 | 930.1886 |
| | LIF | chr22:30240447 | NM_002309 | 6.080869 | 55.56013 | 967.933 |
| | ARC | chr8:142611044 | NM_015193 | 19.45878 | 51.66118 | 986.3858 |
| | IL11 | chr19:55364382 | NM_000641 | 17.02643 | 47.76222 | 1206.981 |
| | NEFL | chr8:24950956 | NM_006158 | 6.080869 | 66.28227 | 1028.324 |
| | NEFM | chr8:24913761 | NM_005382 | 9.72939 | 80.90335 | 866.4426 |
| | | | NM_001105541 | 1.216174 | 0.974739 | 0.838763 |
| | DNER | chr2:229357629 | NM_139072 | 10.94556 | 73.10544 | 1017.42 |
| **IMR90** | HOXC8 | chr12:54009106 | NM_022658 | 205 | 7.101471 | 0.960396 |
| | CCR1 | chr3:46201709 | NM_001295 | 2 | 2.028992 | 182.4752 |
| | OLFML1 | chr11:7485369 | NM_198474 | 0 | 5.072479 | 190.1583 |
| | MYO1D | chr17:32492522 | NM_015194 | 6 | 8.115967 | 193.0395 |
| | | | NM_001303280 | 0 | 0 | 0.960396 |
| | TMEM178B | chr7:141074232 | NM_001195278 | 8 | 16.23193 | 171.9108 |
| | FGF7 | chr15:49423178 | NM_002009 | 574 | 76.08719 | 115.2475 |
| | TBX2 | chr17:61399896 | NM_005994 | 839 | 246.5225 | 54.74255 |
| | PTGDS | chr9:136977504 | NM_000954 | 855 | 188.6962 | 97.96035 |
| | TRIM58 | chr1:247857199 | NM_015431 | 1112 | 30.43487 | 0.960396 |
| | GAL | chr11:68684475 | NM_015973 | 406 | 184.6382 | 19.20791 |
| | TRPA1 | chr8:72021251 | NM_007332 | 392 | 32.46387 | 20.16831 |
| | KRT7 | chr12:52233170 | NM_005556 | 273 | 67.97122 | 21.1287 |
| | LINC00842 | chr10:46398362 | NR_033957 | 237 | 114.638 | 11.52475 |
| | TTC9 | chr14:70641787 | NM_015351 | 27 | 134.9279 | 523.4156 |
| | PPL | chr16:4882507 | NM_002705 | 50 | 95.36261 | 711.6532 |
| | BEX1 | chrX:103062653 | NM_018476 | 45 | 110.58 | 867.2372 |
| | SLC1A1 | chr9:4490427 | NM_004170 | 46 | 191.7397 | 634.8215 |
| | IFI44L | chr1:78620382 | NM_006820 | 74 | 166.3773 | 587.7621 |
| | PNMA2 | chr8:26504680 | NM_007257 | 10 | 57.82626 | 491.7226 |
| | TBX18 | chr6:84732496 | NM_001080508 | 11 | 83.18866 | 249.7029 |
| | G0S2 | chr1:209675325 | NM_015714 | 7 | 120.725 | 301.5642 |
| | C3 | chr19:6677835 | NM_000064 | 54 | 14.20294 | 250.6633 |
| | ARFGEF3 | chr6:138161916 | NM_020340 | 6 | 36.52185 | 228.5742 |
| | PDK3 | chrX:24465227 | NM_005391 | 1 | 0 | 1.920791 |
| | | | NM_001142386 | 3 | 32.46387 | 192.0791 |
| | GBP4 | chr1:89181148 | NM_052941 | 3 | 36.52185 | 183.4356 |
| | FSTL3 | chr19:676389 | NM_005860 | 4814 | 3318.416 | 830.7422 |
| | CDH6 | chr5:31193655 | NM_004932 | 5854 | 3841.896 | 537.8215 |

| | | | | | | |
|---|---|---|---|---|---|---|
| | ID3 | chr1:23557930 | NM_002167 | 7182 | 6114.366 | 978.6431 |
| | XYLT1 | chr16:17102324 | NM_022166 | 7273 | 7896.835 | 1476.128 |
| | COL5A3 | chr19:9959561 | NM_015719 | 841 | 1107.829 | 30.73266 |
| | HSPA6 | chr1:161524540 | NM_002155 | 1629 | 520.4364 | 46.09899 |
| | FLNC | chr7:128830429 | NM_001458 | 1210 | 366.233 | 151.7425 |
| | CLDN4 | chr7:73830863 | NM_001305 | 976 | 624.9294 | 98.92075 |
| | KCNG1 | chr20:51003656 | NM_002237 | 952 | 734.495 | 118.1287 |
| | VGF | chr7:101162509 | NM_003378 | 3319 | 912.0317 | 326.5345 |
| | IL11 | chr19:55364382 | NM_000641 | 1695 | 1132.177 | 96.99996 |
| | CRLF1 | chr19:18593225 | NM_004750 | 1546 | 1221.453 | 106.6039 |
| | PTGS2 | chr1:186671812 | NM_000963 | 2219 | 750.7269 | 153.6633 |
| | AEBP1 | chr7:44104361 | NM_001129 | 2235 | 799.4227 | 259.3068 |
| | DPP4 | chr2:161992245 | NM_001935 | 551 | 911.0172 | 4052.87 |
| | CHI3L1 | chr1:203178931 | NM_001276 | 127 | 751.7414 | 2475.9 |
| | FOXE1 | chr9:97853255 | NM_004473 | 116 | 790.2922 | 1026.663 |
| | ENDOD1 | chr11:95089810 | NM_015036 | 153 | 575.2191 | 1290.772 |
| | LINC02154 | chrX:13266048 | NR_146309 | 78 | 167.3918 | 2891.751 |
| | ITGB8 | chr7:20331102 | NM_002214 | 181 | 269.8559 | 1177.445 |
| | CYP1B1 | chr2:38067603 | NM_000104 | 59 | 211.0151 | 1609.623 |
| | GDF15 | chr19:18385960 | NM_004864 | 131 | 154.2034 | 1357.999 |
| | ZNF395 | chr8:28345585 | NM_018660 | 45 | 323.6242 | 1072.762 |
| | CLDN11 | chr3:170418865 | NM_005602 | 121 | 185.6527 | 798.0888 |
| | | | NM_001185056 | 0 | 0 | 1.920791 |
| | HAPLN1 | chr5:83638198 | NM_001884 | 83 | 287.1023 | 735.663 |
| **MRC5** | STC1 | chr8:23841921 | NM_003155 | 2618.236 | 2021.701 | 47810.26 |
| | NAMPT | chr7:106248286 | NM_005746 | 3859.097 | 2480.757 | 54828.89 |
| | C3 | chr19:6677835 | NM_000064 | 80.02519 | 42.89159 | 22286.98 |
| | CXCL5 | chr4:73995642 | NM_002994 | 26.36124 | 30.14003 | 57248.38 |
| | CXCL8 | chr4:73740506 | NM_000584 | 117.6841 | 31.29927 | 84209.91 |
| | MMP3 | chr11:102835797 | NM_002422 | 84.73255 | 79.98701 | 47981.98 |
| | PTGS2 | chr1:186671812 | NM_000963 | 220.3046 | 91.57933 | 22886.63 |
| | GDF15 | chr19:18385960 | NM_004864 | 1527.069 | 322.2665 | 6753.329 |
| | PTGES | chr9:129738336 | NM_004878 | 237.2512 | 106.6494 | 4756.313 |
| | CCL2 | chr17:34255277 | NM_002982 | 269.2612 | 217.9356 | 4232.982 |
| | TNFAIP6 | chr2:151357592 | NM_007115 | 349.2864 | 205.1841 | 4507.368 |
| | BST2 | chr19:17402939 | NM_004335 | 295.6225 | 285.1711 | 5429.556 |
| | CHST2 | chr3:143119776 | NM_004267 | 359.6426 | 274.738 | 3834.124 |
| | IFI44L | chr1:78620382 | NM_006820 | 363.4085 | 346.6104 | 3715.103 |
| | GFPT2 | chr5:180300690 | NM_005110 | 393.5356 | 352.4065 | 3849.57 |
| | OXTR | chr3:8750409 | NM_000916 | 303.1543 | 272.4195 | 7.268482 |
| | | | NM_001354656 | 8.473255 | 6.955392 | 0 |
| | PNLIPRP3 | chr10:116427912 | NM_001011709 | 0 | 0 | 131.7412 |
| | CCL7 | chr17:34270216 | NM_006273 | 0 | 0 | 158.9981 |
| | PI3 | chr20:45174899 | NM_002638 | 0 | 0 | 312.5447 |
| | SAA2 | chr11:18245228 | NM_001127380 | 0 | 1.159232 | 622.3638 |
| | | | NM_030754 | 0 | 0 | 403.4008 |

| | | | | | | |
|---|---|---|---|---|---|---|
| | VNN1 | chr6:132680858 | NM_004666 | 2.824418 | 4.636928 | 239.8599 |
| | TCIM | chr8:40153468 | NM_020130 | 0.941473 | 8.114624 | 326.1731 |
| | SAA2-SAA4 | chr11:18238233 | NM_001199744 | 0 | 0 | 8.177043 |
| | MIR3142HG | chr5:160468251 | NR_132748 | 7.531783 | 1.159232 | 438.8346 |
| | ZSWIM5 | chr1:45016404 | NM_020883 | 5.648837 | 16.22925 | 402.4922 |
| | CTSS | chr1:150730196 | NM_004079 | 12.23915 | 6.955392 | 510.6109 |
| | IL1A | chr2:112773915 | NM_000575 | 17.88798 | 13.91078 | 2240.51 |
| | CXCL10 | chr4:76021116 | NM_001565 | 34.83449 | 44.05082 | 935.8171 |
| | CHI3L1 | chr1:203178931 | NM_001276 | 44.24922 | 26.66234 | 1206.568 |
| | RSPO3 | chr6:127118903 | NM_032784 | 48.95659 | 18.54771 | 437.9261 |
| | LINC02154 | chrX:13266048 | NR_146309 | 23.53682 | 20.86618 | 686.8716 |
| | LAMP3 | chr3:183122215 | NM_014398 | 23.53682 | 23.18464 | 550.5875 |
| | C11orf96 | chr11:43942556 | NM_001145033 | 32.95155 | 20.86618 | 530.5992 |
| | CXCL3 | chr4:74036591 | NM_002090 | 17.88798 | 5.79616 | 5018.887 |
| | CXCL6 | chr4:73836556 | NM_002993 | 12.23915 | 11.59232 | 8162.506 |
| | IL6 | chr7:22727142 | NM_000600 | 41.4248 | 16.22925 | 5042.51 |
| | IL1B | chr2:112829760 | NM_000576 | 29.18566 | 9.273857 | 12543.58 |
| | OAS2 | chr12:112978469 | NM_002535 | 140.2795 | 155.3371 | 1610.877 |
| | | | NM_001032731 | 120.5085 | 135.6302 | 475.177 |
| | | | NM_016817 | 128.0403 | 106.6494 | 1505.484 |
| | IL11 | chr19:55364382 | NM_000641 | 80.96666 | 105.4901 | 3215.395 |
| | MX2 | chr21:41362023 | NM_002463 | 96.9717 | 114.764 | 2185.996 |
| | CA9 | chr9:35673918 | NM_001216 | 130.8647 | 95.05703 | 2521.255 |
| | KCNG1 | chr20:51003656 | NM_002237 | 127.0988 | 77.66855 | 1138.426 |
| | BEX1 | chrX:103062653 | NM_018476 | 44.24922 | 54.48391 | 1275.619 |
| | AREG | chr4:74445098 | NM_001657 | 54.60542 | 62.59853 | 1753.521 |
| | RSAD2 | chr2:6877665 | NM_080657 | 42.36628 | 52.16544 | 2703.875 |
| | BMP2 | chr20:6768098 | NM_001200 | 70.61046 | 40.57312 | 3089.105 |
| | DNER | chr2:229357629 | NM_139072 | 27.30271 | 33.61773 | 2325.914 |
| | CSF2 | chr5:132073792 | NM_000758 | 47.07364 | 16.22925 | 2952.821 |
| | CXCL2 | chr4:74097037 | NM_002089 | 62.13721 | 16.22925 | 2628.465 |
| | OAS1 | chr12:112906777 | NM_001320151 | 0 | 0 | 9.085603 |
| | | | NM_002534 | 16.94651 | 25.50311 | 145.3696 |
| | | | NM_016816 | 12.23915 | 10.43309 | 113.57 |
| **WI38** | TOX | chr8:58805418 | NM_014729 | 342.6031 | 41.70968 | 31.96518 |
| | ALDOC | chr17:28573115 | NM_005165 | 397.3244 | 61.61657 | 30.18933 |
| | TXNIP | chr1:145992426 | NM_006472 | 13.08553 | 54.03299 | 309.8846 |
| | GBP4 | chr1:89181148 | NM_001313972 | 0 | 0 | 0.887922 |
| | ANKRD1 | chr10:90912100 | NR_045839 | 153.4576 | 103.3263 | 94.11969 |
| | ASB5 | chr4:176213675 | NM_080874 | 578.1427 | 355.4802 | 33.74102 |
| | NEFH | chr22:29480192 | NM_021076 | 587.6594 | 143.14 | 31.96518 |
| | MBP | chr18:77012783 | NM_001025100 | 506.767 | 168.7346 | 46.17192 |
| | SOD2 | chr6:159690922 | NM_001322820 | 15.46472 | 26.54252 | 27.52557 |
| | | | NM_001322819 | 8.327158 | 3.791789 | 3.551686 |
| | | | NM_001322816 | 59.4797 | 538.434 | 306.333 |
| | CH25H | chr10:89205937 | NM_003956 | 99.92589 | 768.7852 | 246.8422 |

| | | | | | | |
|---|---|---|---|---|---|---|
| | CRHBP | chr5:76952855 | NM_001882 | 44.01498 | 461.6503 | 115.4298 |
| | CCND2 | chr12:4273736 | NM_001759 | 33.30863 | 310.9267 | 524.7617 |
| | BEX1 | chrX:103062653 | NM_018476 | 17.84391 | 262.5814 | 316.988 |
| | LAMP3 | chr3:183122215 | NM_014398 | 29.73985 | 357.3761 | 277.0315 |
| | SCD | chr10:100347015 | NM_005063 | 40961.29 | 6265.931 | 4842.724 |
| | CXCL8 | chr4:73740506 | NM_000584 | 1684.465 | 23565.02 | 6876.065 |
| | PTGS2 | chr1:186671812 | NM_000963 | 1724.911 | 8148.555 | 1588.492 |
| | TNFRSF11B | chr8:118923557 | NM_002546 | 7964.331 | 4676.224 | 1323.003 |
| | PPL | chr16:4882507 | NM_002705 | 1780.822 | 595.3109 | 178.4722 |
| | RCN3 | chr19:49527618 | NM_020650 | 1753.461 | 831.3497 | 271.704 |
| | DIRAS1 | chr19:2714567 | NM_145173 | 1278.813 | 393.3981 | 193.5669 |
| | PIEZO2 | chr18:10670247 | NM_022068 | 1014.724 | 303.3431 | 105.6627 |
| | KIAA1644 | chr22:44243677 | NM_001099294 | 923.1249 | 277.7485 | 148.2829 |
| | DSG2 | chr18:31498004 | NM_001943 | 77.32361 | 327.0418 | 1462.407 |
| | ADAM23 | chr2:206443644 | NM_003812 | 102.3051 | 599.1027 | 857.7323 |
| | FOXE1 | chr9:97853255 | NM_004473 | 59.4797 | 1007.668 | 987.3688 |
| | NRK | chrX:105822543 | NM_198465 | 52.34213 | 1131.849 | 1269.728 |
| | CXCL5 | chr4:73995642 | NM_002994 | 111.8218 | 4065.746 | 461.7192 |
| | IL1A | chr2:112773915 | NM_000575 | 98.7363 | 2545.238 | 1232.435 |
| | DNER | chr2:229357629 | NM_139072 | 115.3906 | 958.3747 | 492.7965 |
| | CSF2 | chr5:132073792 | NM_000758 | 54.72132 | 1184.934 | 634.864 |
| | CXCL3 | chr4:74036591 | NM_002090 | 92.78833 | 1486.381 | 460.8313 |
| | IL6 | chr7:22727142 | NM_000600 | 137.9929 | 1774.557 | 293.9021 |
| | CXCL2 | chr4:74097037 | NM_002089 | 133.2345 | 1532.831 | 648.1828 |
| | CXCL6 | chr4:73836556 | NM_002993 | 121.3386 | 2141.413 | 537.1926 |
| | PITX1 | chr5:135027734 | NM_002653 | 2655.174 | 816.1826 | 424.4265 |
| | LAMA1 | chr18:6941744 | NM_005559 | 4578.747 | 1315.751 | 213.9891 |
| | TMEM119 | chr12:108589846 | NM_181724 | 4771.461 | 1347.981 | 427.0903 |
| | IL1B | chr2:112829760 | NM_000576 | 140.3721 | 4818.416 | 1659.525 |
| | CXCL10 | chr4:76021116 | NM_001565 | 161.7848 | 2757.579 | 3181.423 |
| | HAS2 | chr8:121613031 | NM_005328 | 277.1754 | 2398.307 | 2986.968 |
| | OAS2 | chr12:112978469 | NM_016817 | 480.596 | 2714.921 | 3042.019 |
| | | | NM_001032731 | 129.6657 | 544.1217 | 700.5701 |
| | | | NM_002535 | 536.5069 | 3290.325 | 3806.52 |
| | CYP1B1 | chr2:38067603 | NM_000104 | 460.3729 | 2829.623 | 1740.326 |
| | BST2 | chr19:17402939 | NM_004335 | 498.4399 | 2912.094 | 2131.012 |
| | C3 | chr19:6677835 | NM_000064 | 229.5916 | 10010.32 | 3421.162 |
| | RSAD2 | chr2:6877665 | NM_080657 | 339.0343 | 4981.463 | 8081.862 |
| | INHBA | chr7:41685114 | NM_002192 | 1006.396 | 5311.348 | 4097.758 |
| | MMP3 | chr11:102835797 | NM_002422 | 472.2688 | 4945.441 | 3629.824 |
| | MX2 | chr21:41362023 | NM_002463 | 452.0457 | 3301.7 | 4825.854 |
| | OAS1 | chr12:112906777 | NM_001320151 | 0 | 4.739736 | 5.32753 |
| | | | NM_002534 | 98.7363 | 276.8006 | 374.7029 |
| | | | NM_016816 | 36.87741 | 250.2581 | 289.4624 |
