# Supplementary material for: Tracking telomere fusions through crisis reveals conflict between DNA transcription and the DNA damage response
Source: NAR Cancer. 2021 Jan 6;3(1):zcaa044. doi: 10.1093/narcan/zcaa044 (PMC7787266; doi:10.1093/narcan/zcaa044)
Supplement: zcaa044_Supplemental_Files [file zcaa044_supplemental_files.zip › List of Supplementary Files with captions.docx]

**Supplementary Figure 1: Extended growth and telomere fusions detected in HPV E6E7-transformed fibroblasts**

(A) Growth curves for four human fibroblast cell lines retrovirally-transduced with HPV E6E7 (squares) or NEO empty-vector controls (circles) plotted as Population Doubling against Days in culture. Cell counts were performed at each passage until cultures ceased expanding. Growth curves were used to define Early (E), Deep (D) and Late (L) time points for each line, as indicated in the accompanying table. (B) DNA extracted at these time points was subjected to chr17p telomere length analysis (measured in kb) by STELA long-range PCR. Each data point represents a single telomere length measurement. Error bars showing means with SD are displayed for each sample point. Statistical significance was determined by Kruskal-Wallis one-way ANOVA of the E, D and L samples of each model with Dunn’s post-Test analysis. (C) Telomere fusions amplified from fibroblasts transduced with E6E7 or NEO control vectors harvested at the Late growth curve time-point and visualised with Chr21q subtelomere probe.

**Supplementary Figure 2: Profiles of telomere fusions in crisis fibroblasts**

For segregated genomic (A), intra-chromosomal (B) and inter-chromosomal (C) fusions, (i) stacked bar charts depicting proportions of all established fusions for each sample that are sequence-verified as linked with chr17p (black), chr21q (white), chrXpYp (light grey), ambiguous telomere (mid-grey), other secondary genomic linkages (hatched bar) at the Early (E), Deep (D) and Late (L) time points are shown. For each fusion category, summaries of proportions of specified fusions for all samples displayed with error bars showing 95% CI in (ii). Statistical significance was assessed by Repeated Measures one-way ANOVA with Tukey’s Multiple Comparison post-Test.

**Supplementary Figure 3: Microhomology usage and insertions at telomere fusion junctions are hallmarks of ANHEJ repair**

For genomic (A), intra-chromosomal (B) and inter-chromosomal (C) fusions, (i) stacked bar charts depicting proportions of all established fusion junctions that have microhomology (MH) or insertions (INS) or are blunt-ended

at the Early (E), Deep (D) and Late (L) time points. Summaries of the proportions of these junction features in all samples are displayed in (ii) with error bars showing 95% CI. Statistical significance was assessed by Repeated Measures one-way ANOVA with Tukey’s Multiple Comparison post-Test. (D)(i) The numbers of genomic, intra-chromosomal and inter-chromosomal fusions with INS templated from flanking (+/- 100 bp from junction) sequence or not (untemplated) are plotted for each sample and time point. A summary for all the samples is illustrated in (ii) with error bars showing 95% CI. Statistical significance was determined by Kruskal-Wallis one-way ANOVA of the % templated INS for all genomic, intra- and inter-chromosomal fusions with Dunn’s post-Test analysis. (E) Vertical scatter plots displaying the bp of MH at each junction of (i) genomic, (ii) intra-chromosomal and (iii) inter-chromosomal fusions for each sample and time point with error bars showing means with 95% CI. Statistical significance was evaluated by Kruskal-Wallis one-way ANOVA with Dunn’s post-Test analysis. (F) Vertical scatter plots displaying the bp of INS at each junction of (i) genomic, (ii) intra-chromosomal and (iii) inter-chromosomal fusions for each sample and time point with error bars showing means with 95% CI. Statistical significance was assessed by Kruskal-Wallis one-way ANOVA with Dunn’s post-Test analysis. (G) Growth curves for DNA ligase 4-deficient (GM16088, GM17523; LIG4mt) and DNA ligase 1-deficient (GM16096; LIG1mt) human fibroblast cell lines retrovirally-transduced with HPV E6E7 (squares) or NEO empty-vector controls (circles) plotted as Population Doubling against Days in culture. Cell counts were performed at each passage until cultures ceased expanding. Growth curves were used to define Early (E), Deep (D) and Late (L) time points for each line. (H) Southern blot depicting telomere fusion amplicons generated using unidirectional primers specific for chr17p, chr21q and chrXpYp subtelomere sequences with LIG4mt and LIG1mt fibroblast genomic DNA samples extracted at the crisis time points defined in (G). Triplicate reaction lanes are presented for each sample time point, with MW markers (in kb) on the left. Blots were hybridised with a radiolabelled probe consisting of chr17p subtelomere sequence. (I) Bar chart exhibiting the proportions of total genomic/intra-chromosomal fusion events for the DNA ligase-deficient fibroblasts compared with the Normal fibroblasts (HCA2, IMR90, MRC5 and WI38). Bar charts depicting proportions of all genomic fusion junctions in HCA2, IMR90, MRC5 and WI38 fibroblasts (Normal; black bars) that have MH (J(i)) or INS (K(i)) in comparison with genomic fusions identified in two different DNA ligase 4-deficient fibroblast lines (LIG4mt; dark grey bars) and one DNA ligase 1-deficient fibroblast line (LIG1mt; light grey bars) at comparable Early, Deep and Late crisis stages are shown. The mean bp of each feature is presented in scatter charts with 95% CI in J(ii) (MH) and K(ii) (INS). Statistical significance was evaluated using chi-squared analyses (i) and Mann-Whitney two-tailed U-tests between the samples indicated (ii). Violin plot representations of the bp MH (L(i)) or INS (M(i)) recorded at genomic, intra-chromosomal and inter-chromosomal fusions for all Normal and DNA ligase-deficient fibroblast lines. Median values are indicated as white circles, box limits indicate the 25th and 75th percentiles and whiskers extend 1.5 times the interquartile range. Mean bp junction MH (L(ii)) or INS (M(ii)) with 95% CI are displayed in accompanying bar charts. Statistical significance was determined by Kruskal-Wallis one-way ANOVA tests with Dunn’s post-Test analysis. Significant differences between paired samples are indicated by connecting lines annotated with * *P*<0.05, ** *P*<0.001, *** *P*<0.0001.

**Supplementary Figure 4: Asymmetric deletion of fused sister chromatids in intra-chromosomal fusions**

(A) The kb deletion from the start of the canonical telomere repeat sequence of (i) chr17p (ii) chr21q and (iii) chrXpYp to the fusion junction was calculated for each chromatid involved in an intra-chromosomal telomere fusion and displayed as a vertical scatter plot for the Early (E), Deep (D) and Late (L) crisis points. Error bars with means and 95% CI are shown and statistical significance was assessed by Kruskal-Wallis one-way ANOVA with Dunn’s post-Test analysis. (B) Difference (Δ) in kb deletion calculated for paired chromatids contributing to intra-chromosomal fusions at (i) chr17p (ii) chr21q and (iii) chrXpYp telomeres. Error bars with means and 95% CI are shown and statistical significance was evaluated by Kruskal-Wallis one-way ANOVA with Dunn’s post-Test analysis. (C) Positions of all (i) chr17p (ii) chr21q and (iii) chrXpYp intra-chromosomal fusion junctions for each crisis fibroblast model, as well as our simulated fusion dataset, were mapped to the GRCh38 human reference sequence and displayed as horizontal plots with centromere-telomere oriented left-right. Simulant junction locations are presented as frequency distributions with bins of 200 bp (chr17p (i) and chr21q (ii)) or 100 bp (chrX (iii)). The start of the telomere repeat arrays and locations of the primers used for telomere fusion amplification are indicated with arrows and the relative size and positions of long-terminal repeat (LTR) sequences within each subtelomere are represented by filled orange rectangles. (D) Subtelomere positions (GRCh38) of all (i) chr17p (ii) chr21q and (iii) chrXpYp intra-chromosomal fusion junctions for pooled HCA2, IMR90, MRC5 and WI38 (Normal; black) crisis fibroblasts in comparison with intra-chromosomal fusion junctions for two different DNA ligase 4-deficient fibroblast lines (LIG4mt; dark grey) and one DNA ligase 1-deficient fibroblast line (LIG1mt; light grey) at comparable Early, Deep and Late crisis stages are displayed as horizontal plots with centromere-telomere oriented left-right. The start of the telomere repeat arrays and locations of the primers used for telomere fusion amplification are indicated with arrows and the relative size and positions of long-terminal repeat (LTR) sequences within each subtelomere are represented by filled orange rectangles. (E)(i) The frequencies of intra-chromosomal fusion junctions within 200bp bins along the chr17p subtelomere are plotted as histograms for the pooled Normal (black), LIG4mt (dark grey) and LIG1mt (light grey) crisis fibroblasts. (ii) The proportions of intra-chromosomal fusion junctions for each sample group mapping to the central under-represented region of chr17p (17:114400-115600) marked by red dotted lines in (i) are shown in a bar chart with statistical significance determined by a Fisher’s exact test.

**Supplementary** **Figure 5: Elevated incidence of telomere fusions with human chromosome 12**

(A) The proportions (%) of all established (black bars), predicted (grey bars) and simulated (white bars) genomic fusions localised to each chromosome are shown as a bar chart. ChrY was omitted since IMR90 and WI38 are homogametic XX. ChrMT was excluded on the basis of unknown copy number. A chi-squared analysis was used to compare simulant frequencies with actual established fusion frequencies, revealing statistically-significant (*P*<0.05) variation at chromosomes 1, 3, 9, 10, 12, 16, 17, 19 and 22 (marked with *). (B) The numbers of all (i) HCA2, (ii) IMR90, (iii) MRC5 and (iv) WI38 sample genomic fusions localised to each chromosome and adjusted for chromosome size (in kb) are shown as a bar chart. ChrY was omitted since IMR90 and WI38 are homogametic XX. ChrMT was excluded on the basis of unknown copy number. Predicted (darker toned bars) fusion frequencies (events/chromosome) were generated using the mean overall fusion frequency (events/kb) for each individual lineage multiplied by the specific chromosome size for comparison with the actual (lighter toned bars) normalised chromosome frequencies. A chi-squared analysis was used to compare predicted with actual frequencies, revealing statistically-significant (*P*<0.05) sample-specific variation at distinct chromosomes, marked with *. (C)(i) Gene density (open bars) and cancer gene density (black bars) were calculated as genes/bp for each chromosome using Ensembl and the Cancer Genetics*Web* resources. Mean genomic values are recorded as red dashed lines (gene density, tight dashes; cancer gene density, wide dashes). Chromosomes are ranked according to their mean (ii) gene density or (iii) cancer gene density.

**Supplementary Figure 6: Genomic telomere fusions are associated with long transcribed genes and not fragile sites**

(A)(i) The proportions of all genomic fusion junctions co-localising with genes identified by Refgene, Ensembl and GeneBase 1.1 are shown for each sample at the Early (E), Deep (D) and Late (L) crisis points and partitioned into intron (light grey) versus exon (dark grey) coincidence. Comparable characteristics for the whole genome and the totality of sample genomic fusions (ALL FUSIONS) are displayed as separate bars. A chi-squared analysis was employed to evaluate differences between individual samples or ALL FUSIONS and the overall genome proportions (significant differences marked with *; *P*<0.05). (ii) The proportions of all telomere fusions with genes for which the junction coincides with exons, promoters, promoter-flanking sequence, enhancers, CTCF or other transcription factor (TF) binding sites or uncharacterised (other) intronic sequence are displayed as a pie chart. The proportions of genes (iii) or exons (iv) captured within telomere fusions that were concurrently expressed (black) or not (white) in crisis fibroblasts (detected by RNA-Seq) are depicted in pie charts. Where reference information or overlapping gene sequences produced ambiguity, the events were recorded as N/A (grey). (B)(i) The mean RNA-Seq DESeq2 normalised read counts for all transcripts pertaining to fused genes on chromosome 12 (black bar) is juxtaposed with that of fused genes on all other chromosomes (white bar; excluding chr12 fused) in IMR90 crisis samples and a comparison of means performed. (ii) The mean RNA-Seq DESeq2 normalised read counts for all transcripts on defined chromosomes (black bars) is compared with mean read counts for all other chromosomes excluding the defined chromosome (white bars). Statistical significance was determined by comparison of means tests. (C) The length in Mbp of each gene disrupted by a telomere fusion event in fibroblast E, D and L crisis phases is plotted as a vertical scatter plot. Error bars with means and 95% CI are shown and statistical significance was assessed by Kruskal-Wallis one-way ANOVA with Dunn’s post-Test analysis. (C) The proportions of all established (central bars) and simulated (right) genomic fusion junctions coincident with (i) genes, (ii) repeats (Repeatmasker) and (iii) fragile sites (HUMCFS) is displayed in comparison with the human genome (left) content of these features. Statistical significance of enrichments in defined datasets was determined by chi-squared analysis.

**Supplementary Figure 7: Intersection of copy number alterations with telomere fusion loci increases with crisis progression**

(A)(i) The proportions of statistically-significant (*P*<0.05) CNA intersections with genomic telomere fusions separated by crisis phase (Early, Deep, Late) are plotted as a stacked bar chart for each crisis fibroblast sample. (ii) Statistical significance of all intersections displayed in (i) presented by crisis stage was assessed by Kruskal-Wallis one-way ANOVA. Error bars show 95% CI. (iii) The mean rate of intersection (CNA/genomic fusions) was calculated and used to generate predicted numbers of overlaps between CNA and genomic telomere fusions at each crisis stage (grey bars). These values were statistically indistinguishable from actual frequencies of overlaps at each stage, as assessed by chi-squared analysis. (B) The statistically-significant (*P*<0.05) CNA intersections with genomic telomere fusions are displayed per chromosome in (i), per chromosome normalised to chromosome size (Mbp) in (ii) and adjusted to fusion frequency for the same chromosome in (iii). The red dotted line on each plot marks the mean value for all samples.

**Supplementary Figure 8: Genes differentially-expressed with crisis progression and incorporated into telomere fusions are associated with DDR**

Heatmaps representing the top 50 differentially-expressed genes measured by RNA-Seq during crisis transit in HCA2 (A), IMR90 (B), MRC5 (C) and WI38 (D) fibroblasts. Samples from the 3 crisis time points are displayed as a dendrogram with the Refgene gene identities and genome locations are detailed on the right. (E) The STRING protein-protein interaction network is exhibited for the overlapping gene entries in the top 50 differentially-expressed crisis gene lists for each fibroblast lineage. (F) The incidence of top 50 differentially-regulated genes for all fibroblasts (white bars) is displayed by chromosome (i) in comparison with the chromosome frequencies of genomic telomere fusions (black bars) and as adjusted to the specific gene density (determined using GeneBase 1.1) for each chromosome (ii). (G) RNA-Seq DESeq2 normalised read counts for 15 inflammatory and senescence-associated genes is displayed for each fibroblast sample and crisis time point, Early (E), Deep (D) and Late (L). (H) Horizontal bar charts illustrating the statistical significance (x axis) of enriched motifs associated with each transcription factors listed in the colour key within (i) all fibroblast top 50 differentially-regulated genes and (ii) all genes disrupted by genomic telomere fusions, as identified using the X2K Expression2Kinases webtool. Only statistically-significant enrichments are presented (hypergeometric *P*<0.05). (I) Cartoon depicting senescence and DDR signalling, reproduced courtesy of Cell Signaling Technology, Inc. Potential interactions between PTSG2 and TP53 in effecting the senescence-associated secretory phenotype (SASP) have been appended.

**Supplementary Figure 9: Telomere fusions do not impact population-level chr12 gene expression**

(A) Locations of all established genomic telomere fusion junctions along human chromosome 12 in comparison with the simulated fusion dataset (B). (C) Comparison of *DTX*1 (i) and *DTX4* (ii) mRNA expression measured by RT-PCR (normalised to *YWHAZ* gene expression) in NEO versus E6E7 Early and Late crisis HCA2, IMR90, MRC5 and WI38 fibroblast cells. Data are plotted as means with 95% CI and were assessed by Repeated Measures one-way ANOVA with Tukey’s Multiple Comparison post-Test, annotated as * *P*<0.05, ** *P*<0.001, *** *P*<0.0001. (D)(i) The sequences of telomere fusions with chr12 genes, *DENR*, *NCOR2* and *CHFR,* that were also identified within CNA intersections revealed by WGA-Seq are displayed. Genomic sequence is in red (and dark red where more than one genomic site is involved) to the left and telomeric sequence in blue to the right. MH at the fusion junction is displayed in purple and underlined; INS are boxed in green. GRCh38 human reference locations are noted above and below the fusion sequences. (ii) The locations of telomere fusions with genes harboured on chr12 are depicted with arrowheads on the chromosome cartoon to the left. RNA-Seq and RT-PCR data (as generated in (A), but with a single analysis of each of the four fibroblast lines) for fused genes also intersecting with mapped CNA is illustrated in bar charts with SD to the right. Statistical significance was assessed by Repeated Measures one-way ANOVA with Tukey’s Multiple Comparison post-Test.

**Supplementary** **Table 1:** Enumeration of chr17p telomere length data presented in Supplementary Figure 1B. For each crisis sampling time point, the mean chr17p telomere length (in kb) and 95% CI is listed, as well as the mean length with 95% CI for the shorter chr17p allele. The shortest and longest telomere lengths measured for each sample are also listed.

**Supplementary Table 2:** Summary of samples and associated read-counts pertaining to all fusion amplicon (Fusion-Seq), RNA (RNA-Seq) and single nuclei whole genome amplification (WGA-Seq) Illumina HiSeq paired-end sequencing described in this study. Fusion-Seq data was obtained using two flow-cell lanes with each sample featuring in both lanes.

**Supplementary Table 3: Top 50 differentially-expressed genes for each fibroblast sample transiting crisis**

For each fibroblast line, the top 50 genes found to be differentially expressed over time in crisis by RNA-Seq are listed, with details of GRCh38 human reference location, Refgene transcript identities and DESeq2 normalised read counts provided.

**Supplementary Table 4: GO ontology analysis of the top 50 differentially-regulated genes for each fibroblast line**

Each PANTHER Over-representation Test with associated *P* and FDR values is displayed as an individual tab.

**Supplementary Table 5: Gene Set Enrichment Analysis (GSEA) of the top 50 differentially-regulated genes for each fibroblast line**

Overlaps with specified gene signatures alongside the appropriate *P* and FDR values are recorded.

**Supplementary Table 6: GO ontology analysis of the genes disrupted by telomere fusions for each fibroblast line**

Each PANTHER Over-representation Test with associated *P* and FDR values is displayed as an individual tab.

**Supplementary Table 7: Gene Set Enrichment Analysis (GSEA) of the genes disrupted by telomere fusions for each fibroblast line**

Overlaps with specified gene signatures alongside the appropriate *P* and FDR values are recorded.

**Supplementary Table 8: X2K Expression2Kinases analysis of the top 50 differentially-regulated genes for each fibroblast line**

Transcription factors with binding motifs enriched within this gene set are listed, along with the hypergeometric *P* values and the relevant genes containing the motif. Transcription factors for which an enrichment with *P*<0.05 was returned are highlighted in yellow.

**Supplementary Table 9: X2K Expression2Kinases analysis of the genes disrupted by telomere fusions for each fibroblast line**

Transcription factors with binding motifs enriched within this gene set are listed, along with the hypergeometric *P* values and the relevant genes containing the motif. Transcription factors for which an enrichment with *P*<0.05 was returned are highlighted in yellow**.**
